# An improved synthesis of adefovir and related analogues

**DOI:** 10.3762/bjoc.15.77

**Published:** 2019-03-29

**Authors:** David J Jones, Eileen M O’Leary, Timothy P O’Sullivan

**Affiliations:** 1 School of Chemistry, University College Cork, Cork, Ireland; 2 Analytical and Biological Chemistry Research Facility, University College Cork, Cork, Ireland; 3 School of Pharmacy, University College Cork, Cork, Ireland,; 4 Department of Physical Sciences, Cork Institute of Technology, Cork, Ireland

**Keywords:** acyclic nucleoside phosphonate, adefovir, alkylation, antiviral, *N*-alkylation, purine

## Abstract

An improved synthesis of the antiviral drug adefovir is presented. Problems associated with current routes to adefovir include capricious yields and a reliance on problematic reagents and solvents, such as magnesium *tert*-butoxide and DMF, to achieve high conversions to the target. A systematic study within our laboratory led to the identification of an iodide reagent which affords higher yields than previous approaches and allows for reactions to be conducted up to 10 g in scale under milder conditions. The use of a novel tetrabutylammonium salt of adenine facilitates alkylations in solvents other than DMF. Additionally, we have investigated how regioselectivity is affected by the substitution pattern of the nucleobase. Finally, this chemistry was successfully applied to the synthesis of several new adefovir analogues, highlighting the versatility of our approach.

## Introduction

**Figure 1:**
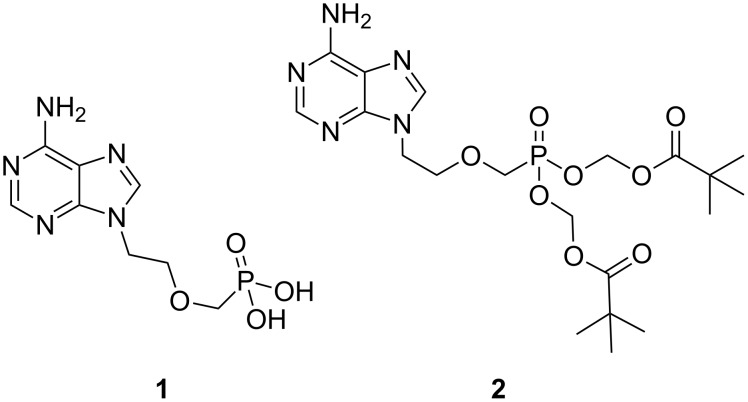
Adefovir (**1**) and its prodrug **2**.

The acyclic nucleoside phosphonate adefovir (**1**) [1], administered as its dipivoxil prodrug form (**2**) [2], is used clinically for the treatment of infections caused by the hepatitis B virus (HBV) [3–5] and the herpes simplex virus (HSV, Figure 1) [5–6]. While **1** possesses inhibitory activity against the human immunodeficiency virus (HIV) [1,5], it has not been approved by the FDA for the treatment of HIV as the dose required to elicit inhibition can cause nephrotoxicity [7].

The mode of action of adefovir has been widely studied and involves the inhibition of viral replication by the termination of DNA synthesis [8–10]. Although adefovir was described in 1986 for the first time by Holý, De Clercq and co-workers, it is still actively employed in research today [11–13]. Adefovir is often used as a benchmark against which the relative activity of other antiretroviral drugs is measured [14–17]. In addition, the development of new prodrugs of current antiretrovirals remains an important field of study [18]. The efficacy of investigational prodrug strategies is typically measured by derivatising proven therapeutics such as adefovir and comparing the properties of the resulting compounds to the parent substrate [15,19–30]. Additionally, the solid state and spectroscopic properties of adefovir have been the subject of several recent studies [31–34].

**Scheme 1:**
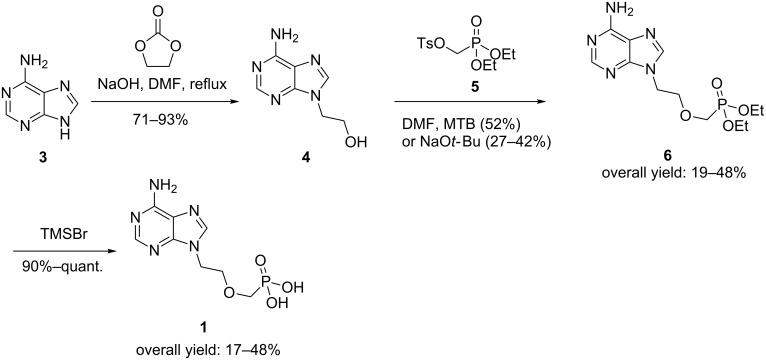
Literature syntheses of **1**.

It is clear, therefore, that there is still demand for a robust synthetic route to adefovir. The most widely employed method for accessing adefovir is shown in Scheme 1 [17,35–36]. Alkylation of commercially available adenine (**3**) to form alcohol **4** and further base-mediated alkylation with tosylate **5** affords phosphonate ester intermediate **6**. Subsequent dealkylation of **6** using trimethylsilyl bromide (TMSBr) gives adefovir (**1**). The related analogue tenofovir, developed as an anti-HIV agent, may be prepared in a similar manner [37–38].

The poor solubility of adenine and its derivatives in most organic solvents restricts the choice of solvent for this and subsequent reactions to polar aprotic solvents such as DMF, NMP and DMSO [38].

The choice of base for the alkylation of alcohol **4** with tosylate **5** has been the subject of recent studies [35,37–39]. While it was demonstrated that magnesium *tert*-butoxide (MTB) is the optimum base for this transformation (Scheme 1), it is not without drawbacks. MTB is expensive and hydrolyses on storage when exposed to moisture. The yield for the reaction is often inconsistent, being highly dependent on the quality of the MTB employed. The reaction does not proceed to completion when a stoichiometric amount of the base is used and up to 3.0 equivalents may be required in order to ensure complete consumption of the starting alcohol. The resultant magnesium salts are highly deliquescent and form a sticky resin on exposure to atmospheric moisture. This resin hinders the isolation of phosphonate **6** and stymies scale-up. Finally, several byproducts, including the ethyl ether of **4** and the hemi-dealkylated ester of **6**, are produced during this reaction, further reducing the atom economy of this approach [35,38]. In an attempt to overcome these difficulties, some groups have explored telescoping the MTB-mediated alkylation of **4** with the subsequent dealkylation of **6** [38,40]. However, as trimethylsilyl bromide (TMSBr) is moisture sensitive, telescoping the two reactions is difficult due to the hygroscopic nature of both the polar aprotic solvents employed and MTB itself. An improved synthetic route to adefovir (**1**), which avoids at least some of these difficulties and especially the use of MTB, would be highly desirable.

## Results and Discussion

We initially investigated the use of phosphonate reagents bearing alternative leaving groups in an effort to improve the in situ conversion from **4** to **6**, thereby improving the yield of this reaction. We selected iodide **7** and triflate **8** for this study and compared the conversion of **4** to **6** by ^1^H NMR (Table 1, entries 1–3). Unfortunately, we found that tosylate **5** remained the superior alkylating agent under these conditions. The reaction of **4** with iodide **7** afforded only a trace amount of the phosphonate, with mostly unreacted starting material evident in the ^1^H and ^31^P NMR spectra of the crude reaction mixture. The reaction with triflate **8** resulted in a significantly lower conversion to **6** than when tosylate **5** was employed. Optimisation of other aspects of this reaction (e.g., choice of base, solvent, temperature and the role of additives) has already been investigated by Brown Ripin [37,40] and Riley [38] which suggested that further investigation had limited potential.

**Table 1:** Impact of the leaving group on in situ conversion of **4** to **6**.

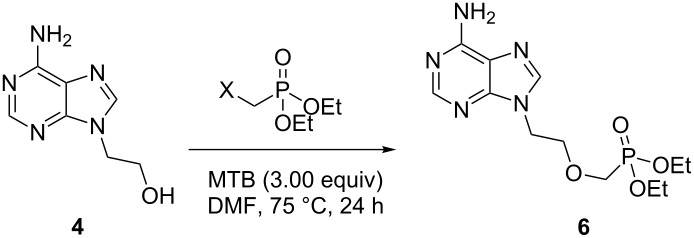				

Entry	Electrophile^a^	Leaving group (X)	Conversion^b^	Yield^c^

1	**5**	OTs	85%	43%
2	**7**	I	56%	24%
3	**8**	OTf	trace	–

^a^Reactions were carried out on a 3 mmol scale, 0.1 M concentration using 1.50 equiv of the electrophile. ^b^Measured by analysis of the ^1^H NMR spectrum of the crude reaction mixture after removal of the solvent in vacuo, comparing the NC*H*_2_ signals of **4** and **6** at 4.19 ppm and 4.33 ppm, respectively. ^c^Yield following purification by column chromatography.

**Figure 2:**
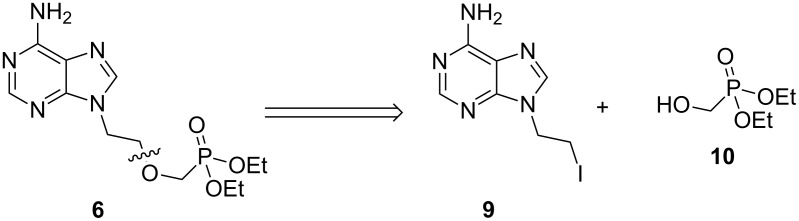
Retrosynthetic analysis of **6** to synthons **9** and **10**.

Given the issues encountered in employing MTB, we next investigated the introduction of the phosphonate ester as the nucleophile rather than as the electrophile (Figure 2).

**Scheme 2:**
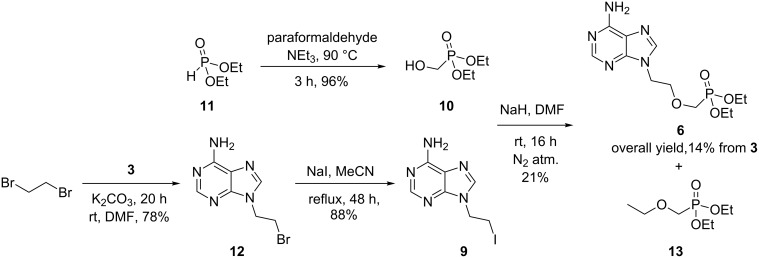
Forward synthesis of **6** from **9** and **10**.

Commercially available alcohol **10** was prepared by adapting a previously reported literature procedure where diethyl phosphonate (**11**) was condensed with paraformaldehyde in the presence of catalytic triethylamine (Scheme 2) [41]. Iodide **9** was accessed in two steps from adenine (**3**). The highly regioselective alkylation of adenine (**3**) with an excess (4.0 equiv) of 1,2-dibromoethane furnished bromide **12** in 78% yield. Despite the poor solubility of **12** in acetonitrile, the compound was successfully converted to iodide **9** via a Finkelstein reaction. Unfortunately, subsequent alkylation of **10** with iodide **9** in the presence of sodium hydride furnished **6** in a poor yield of 21%. Formation of self-alkylation product **13** constituted the major reaction pathway when the reaction was stirred for prolonged periods of time or heated to higher temperatures. Consequently, the overall yield of this process, at 14%, is lower than that of the process outlined in Scheme 1.

**Figure 3:**
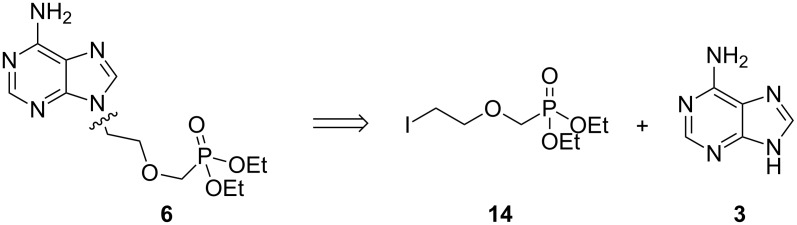
Retrosynthesis of **6** to synthons **14** and **3**.

Given the ease of alkylation of **3** with 1,2-dibromoethane, we wondered if alkylation of adenine with advanced intermediate **14** might represent a facile route to **6** (Figure 3).

**Scheme 3:**
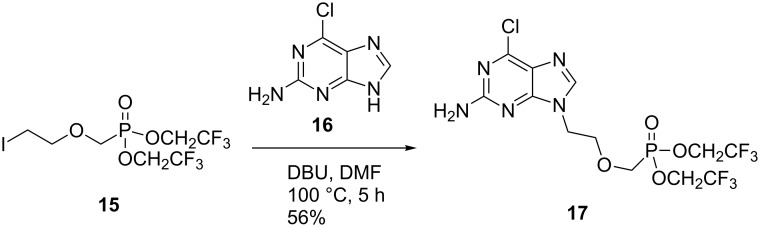
Application of related alkyl iodide **15** [52].

Holý and co-workers have previously utilised a similar alkyl chloride reagent to access adefovir analogues, but found that recourse to an isopropyl ester intermediate was necessary as the corresponding ethyl ester was prone to unwanted dealkylation under their harsh reaction conditions [13]. We reasoned that iodide **14** would facilitate alkylation under much milder conditions, and that the ethyl esters would, therefore, remain intact. Other comparable alkylating agents have also been employed in the synthesis of acyclic nucleoside phosphonates including mesylates [42], tosylates [16,43–44] and alkyl chlorides [45–51]. Alkylation reactions conducted with these electrophiles generally require higher temperatures. Furthermore, these reagents typically afford products in low to moderate yields as the reactions fail to reach completion or else furnish multiple side-products. The successful application of such iodide-based electrophiles is precedented, as demonstrated by the work of Ubasawa et al. in their preparation of purine analogue **17** from **15** (Scheme 3) [52]. A similar approach is outlined in a 1998 patent by the same group [53].

**Scheme 4:**
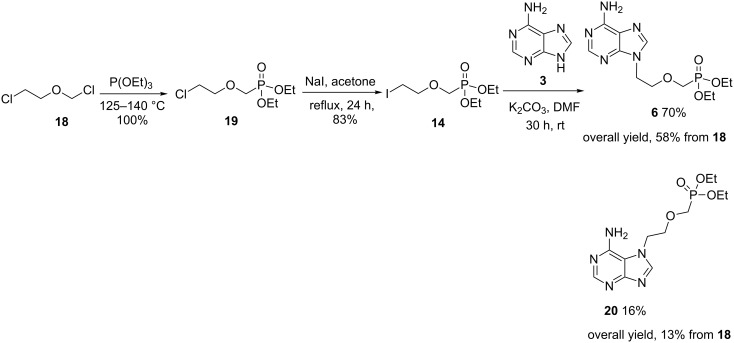
Synthesis of **6** and **20** via iodide **14**.

Iodide **14** was prepared over two steps from commercially available 2-chloroethyl chloromethyl ether (**18**, Scheme 4). Firstly, **18** was subjected to an Arbuzov rearrangement to furnish phosphonate **19** in quantitative yield. A subsequent Finkelstein reaction afforded iodide **14**. During the optimisation of this transformation, we observed that 2.0 equivalents of sodium iodide were required in order to achieve complete consumption of **19**. Iodide **14** was stored under a nitrogen atmosphere over copper wire in an amber container and found to be stable for over one year. Gratifyingly, alkylation of adenine with iodide **14** proceeded smoothly under mild conditions to furnish phosphonate **6** in 70% yield. The overall yield of **6** from **18** was 58% which compared very favourably with that reported in Scheme 1. Interestingly, the unnatural *N*7-regioisomer **20** was also isolated in 16% yield, which was readily separable from **6** by column chromatography. The synthesis of **20** has been reported only once before and the resulting phosphonic acid also possesses antiviral activity [54]. The authors report that **20** was prepared in four steps while our three step route affords **20** in a comparable overall yield of 13%.

We conducted a temperature, solvent and base screen to determine what impact these parameters have on the regioselectivity of the alkylation (Table 2). The overall regioselectivity appeared insensitive to the reaction conditions with only a slight decrease in selectivity for **6** observed when hydroxylic bases were employed in the reaction (Table 2, entries 1 and 2). Temperature was observed to have no effect on the regioselectivity (Table 2, entries 7, 9 and 10). Similarly, no improvement was observed when other aprotic solvents were used in place of DMF (Table 2, entries 8, 12, 13, 15 and 16). The same regioselectivity was observed when the reaction was performed in either NMP or DMF, even though adenine (**3**) was more soluble in NMP. No reaction was observed in acetonitrile, acetone or ethyl acetate, presumably due to the insolubility of adenine (**3**) at room temperature. When wet DMF was employed (Table 2, entry 11), no change in the regioselectivity occurred, although the reaction was found to proceed more slowly, having not reached completion after 30 hours. Conducting the reaction in ethanol (Table 2, entry 17) resulted in degradation of the iodide starting material and the formation of only trace amounts of product. No reaction was observed in cyrene (Table 2, entry 18), often employed as a green alternative to DMF [55].

**Table 2:** Impact of base, solvent and temperature on reaction performance and regioselectivity.

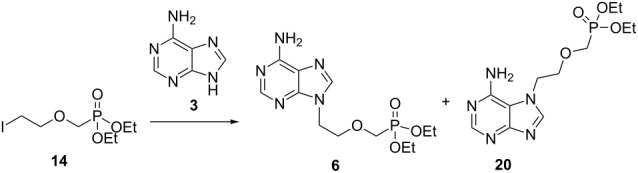					

Entry	Base	Solvent	Temperature (°C)	*N*9:*N*7^a^	Conversion^b^

**1**	NaOH	DMF	rt	3:1	100%
**2**	KOH	DMF	rt	3:1	100%
**3**	NEt_3_	DMF	rt	–^c^	trace
**4**	DBU	DMF	rt	4:1^d^	33%
**5**	DMAP	DMF	rt	–^c^	N.P.
**6**	Cs_2_CO_3_	DMF	rt	4:1	100%
**7**	K_2_CO_3_	DMF	rt	4:1	100%
**8**	K_2_CO_3_	NMP	rt	4:1	100%
**9**	K_2_CO_3_	DMF	0	4:1	100%
**10**	K_2_CO_3_	DMF	50	4:1	100%
**11**	K_2_CO_3_	water:DMF (30% v/v)	rt	4:1	59%
**12**	K_2_CO_3_	MeCN	rt	–^c^	N.P.
**13**	DBU	MeCN	rt	–^c^	N.P.
**14**	NaH	DMF	rt	4:1	100%
**15**	K_2_CO_3_	EtOAc	rt	–^c^	N.P.
**16**	K_2_CO_3_	acetone	rt	–^c^	N.P.
**17**	K_2_CO_3_	EtOH	rt	–^e^	trace
**18**	K_2_CO_3_	cyrene	rt	–^c^	N.P.

^a^Determined by integration of the NC*H*_2_ signals from **6** and **20** at 4.33 ppm and 4.50 ppm, respectively, in the ^1^H NMR spectra of the crude reaction mixture after removal of the solvent in vacuo. ^b^Determined by integration of the C(8)*H* in **3**, **6** and **20** in the ^1^H NMR spectra of the crude reaction mixture. ^c^No reaction observed, only starting material evident in the ^1^H NMR spectra of the crude reaction mixtures. ^d^Reaction did not reach completion. ^e^Degradation of iodide **14** evident in ^1^H NMR and ^31^P NMR spectra of crude reaction mixture. Only traces of product formed. N.P. = no product formed.

**Scheme 5:**
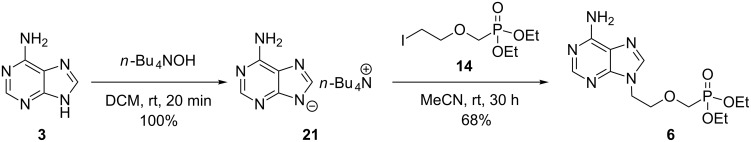
Synthesis of phosphonate **6** using novel salt **21**.

In a bid to replace DMF as the solvent, we turned our attention to more soluble adenine analogues and investigated the formation of **21**, a novel tetrabutylammonium salt of adenine (Scheme 5). Preparation of **21** was facile and the salt was isolated quantitatively as a colourless powder following work-up. Gratifyingly, the subsequent alkylation with iodide **14** in acetonitrile proceeded in comparable yields to those in DMF, constituting an attractive alternative to current literature methods for accessing **6**.

**Scheme 6:**
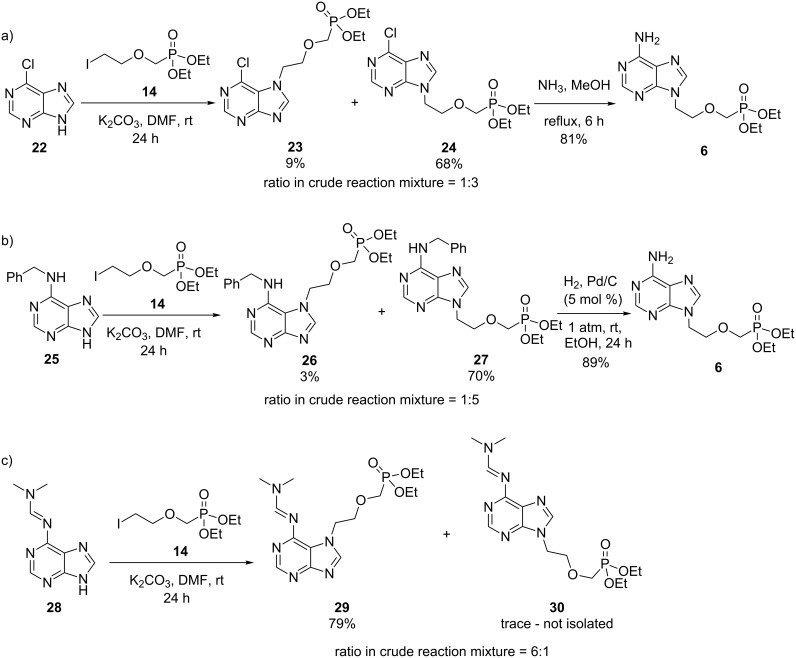
Application of iodide **14** in the synthesis of adefovir analogues.

To explore the utility of iodide **14** in the synthesis of novel antivirals, we examined its reactivity towards other 6-substituted purine nucleobase analogues (Scheme 6). Alkylation of both 6-chloropurine (**22**) and *N*6-benzyladenine (**25**) afforded the corresponding *N*9-regioisomers as the major product as determined by ^1^H NMR spectroscopy (Scheme 6a,b). The major isomers were then isolated and subsequently converted to phosphonate **6** in order to confirm the degree of regioselectivity. Interestingly, the amidine moiety in **28** favoured alkylation at *N*7 and **29** was isolated in 79% yield following purification by column chromatography (Scheme 6c).

**Figure 4:**
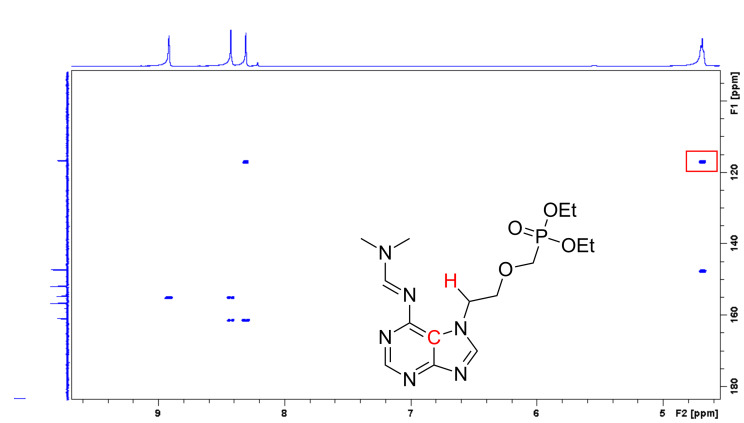
HMBC spectrum confirms *N*7-selectivity for the major product **29**.

Pal and co-workers previously reported the *N*7-selective alkylation of **28** using other electrophiles [56]. They confirmed that the reaction had occurred at *N*7 via HMBC analysis and the identification of a correlation between the methylene protons adjacent to the purine ring with C-5. A similar analysis by us revealed the same correlation, confirming **29** as the major product (Figure 4). While the amidine is itself an important moiety in medicinal chemistry [57], it can also be readily converted to other functional groups including nitrogen-containing heterocycles [58]. Consequently, the use of the amidine moiety to direct the *N*7-selective alkylation with iodide **14** may likewise facilitate the preparation of other *N*7-functionalised adefovir analogues for evaluation.

**Scheme 7:**
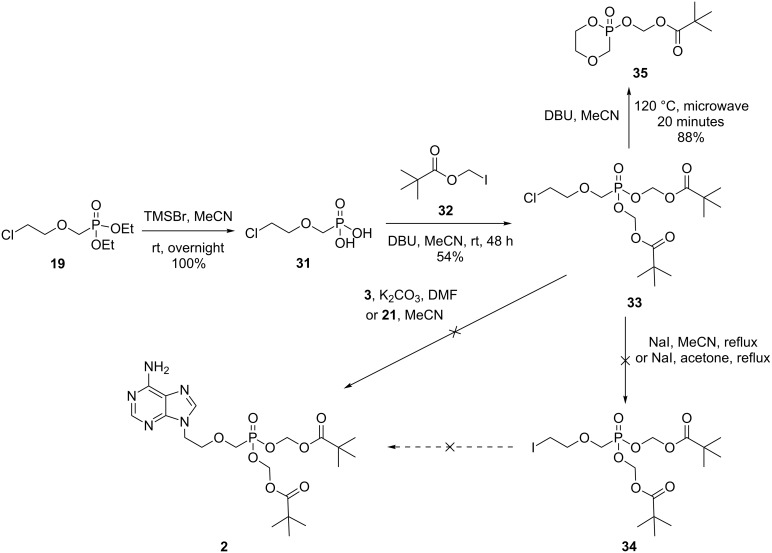
Attempted synthesis of adefovir dipivoxil (**2**) exploiting phosphonate **33**.

Finally, we investigated whether a similar strategy could be exploited to access adefovir dipivoxil (**2**) directly. Although we were able to successfully prepare novel phosphonate **33** via phosphonic acid **31**, subsequent attempts at converting **33** to the corresponding iodide **34**, or alkylation of salt **21** were unsuccessful and hence we were unable to access **2** (Scheme 7). Instead, the major product isolated in both cases was novel heterocycle **35**. It is likely that cleavage of one of the pivaloxymethyl groups, followed by intramolecular cyclisation, results in the formation of **35**. Microwave heating of **33** in the presence of DBU also furnishes cyclic phosphonate **35** in excellent yields.

## Conclusion

We have described a synthetic route to phosphonate intermediate **6** which has a number of distinct advantages when compared to previous approaches. While the standard approach to adefovir relies heavily on the use of magnesium *tert*-butoxide, an expensive, troublesome and capricious reagent, our improved synthesis of adefovir avoids this. As adenine is introduced later in our synthetic sequence, fewer steps are carried out in hazardous, environmentally harmful solvents such as DMF or NMP. Furthermore, the introduction of a more reactive electrophile in **14** means that the critical alkylation step is conducted at room temperature. Additionally, the preparation of chloride **19** is a solventless reaction and the subsequent conversion of **19** to iodide **14** takes place in acetone, a green solvent. Our route also produces fewer byproducts and is higher yielding than the standard synthesis of adefovir, making it a highly attractive alternative for those interested in the study of this molecule and its analogues, particularly on a laboratory scale. In addition, through the use of salt **21**, the preparation of **6** can be achieved without the use of DMF as a solvent. Furthermore, this approach gives synthetically useful quantities of *N*7-substituted analogue **20**, via a more concise route than the current literature procedure. The preparation of a number of novel adefovir analogues using iodide **14** highlights the utility of this reagent. Finally, strategic incorporation of an amidine moiety allows for regioselective alkylations with **14** and facilitates the synthesis of novel *N*7-substituted adefovir analogues.

## Supporting Information

File 1 Experimental part and NMR spectra.
