# Supplementary material for: An improved synthesis of adefovir and related analogues
Source: Beilstein J Org Chem. 2019 Mar 29;15:801–10. doi: 10.3762/bjoc.15.77 (PMC6444443; doi:10.3762/bjoc.15.77)
Supplement: File 1 — Experimental part and NMR spectra. [file Beilstein_J_Org_Chem-15-801-s001.pdf]

BEILSTEIN JOURNAL OF ORGANIC CHEMISTRY

Supporting Information

for

An improved synthesis of adefovir and related analogues

David J. Jones, Eileen M. O'Leary and Timothy P. O'Sullivan

Beilstein J. Org. Chem. **2019,** *15,* 801–810. doi:10.3762/bjoc.15.77

Experimental part and NMR spectra

License and Terms: This is a supporting information file under the terms of the Creative Commons Attribution License (http://creativecommons.org/licenses/by/4.0). Please note that the reuse, redistribution and reproduction in particular requires that the authors and source are credited.
The license is subject to the *Beilstein Journal of Organic Chemistry* terms and conditions: (https://www.beilstein-journals.org/bjoc)

Experimental

General procedures

N,N-Dimethylformamide (DMF) was pre-dried over 4 Å molecular sieves prior to distillation. Triethylamine was distilled from calcium hydride and stored under nitrogen in an amber bottle. ^{1}H, ^{13}C and ^{31}P NMR spectra were recorded on a Bruker Avance 400 NMR spectrometer at 20 °C in $CDCl_3$ or DMSO-d_6 using tetramethylsilane (TMS) as an internal standard and 85% aq. phosphoric acid as an external standard for ^{31}P NMR. Chemical shifts are expressed in parts per million (ppm) relative to the reference peak.

*N*9-(2-Hydroxyethyl)adenine (4)[1]

To a stirred solution of adenine (14.9 g, 110 mmol, 1.00 equiv) in distilled *N,N*-dimethylformamide (80 mL) at room temperature under nitrogen was added ethylene carbonate (10.0 g, 113 mmol, 1.13 equiv) and sodium hydroxide (0.50 g, 12.5 mmol). The reaction mixture heated to reflux and allowed to stir until it was evident that the starting material, adenine, had been consumed by TLC (R_f = 0.05 – eluent: 10% MeOH in CH_2Cl_2). On cooling, the solvent was removed in vacuo and the off-white/yellow solid residues, containing a 9:1 mixture of the *N*9 and *N*7 regioisomers by ^{1}H NMR analysis, were recrystallized from methanol yielding the title compound as an off-white powder (14.20 g, 75 mmol, 68%). Spectroscopic data are consistent with those reported in the literature.

Melting Point (MeOH): 238-239 °C (lit: 238-239 °C) [1]

^{1}H NMR[†] (400 MHz, DMSO-d$_6$): 3.40 (1H, bs, O**H**), 3.74 (2H, t, J = 5.45 Hz, C**H$_2$**OH), 4.19 (2H, t, J = 5.45 Hz, N-C**H$_2$**), 7.22 (2H, bs, N**H$_2$**), 8.08 (1H, s, Ar-**H**), 8.14 (1H, s, Ar-**H**). ^{13}C NMR[†] (75 MHz, DMSO-d$_6$): 45.68 (N-**C**H$_2$), 59.21 (**C**H$_2$-O), 118.62 (ArC), 141.32 (ArC), 149.49 (ArC), 152.22 (ArC), 155.87 (ArC). IR (KBr, cm^{-1}): 3311, 3246, 3056, 2930, 2865, 1687, 1607, 1574, 1067, 1018.

***O,O*-Diethyl hydroxymethylphosphonate (10) [2]**

Adapted from Phillion et al. To a stirred suspension of paraformaldehyde (2.22 g, 72 mmol, 1.00 equiv) in diethyl phosphite (**11**) (10.00 g, 72 mmol, 1.00 equiv) was added triethylamine (732 mg, 1.00 mL, 7 mmol, 0.10 equiv). The resulting suspension was heated to 90 °C and the suspension became clear and the mixture began reflux. The reaction was maintained at this temperature for three hours. The product was then purified by flash column chromatography (R_f **10** = 0.60, eluent – 100% EtOAC) and was isolated as a colourless oil (11.62 g, 70 mmol, 96%).

^{1}H NMR (400 MHz, $CDCl_3$): 1.35 (6H, t, *J* = 7.19 Hz, $OCH_2C\underline{\mathbf{H_3}}$), 3.91 (2H, dd appears as a triplet, J_{H-H} = J_{H-P} = 6.30 Hz, $HOC\underline{\mathbf{H_2}}P$), 4.13-4.24 (4H, m, $OC\underline{\mathbf{H_2}}CH_3$), 4.81 – 4.86 (1H, m, $\underline{\mathbf{H}}OCH_2P$). ^{13}C NMR (100 MHz, $CDCl_3$): 16.35 (d, *J* = 5.68 Hz, $OCH_2\underline{\mathbf{C}}H_3$), 56.87 (d, *J* = 162.21 Hz, $HO\underline{\mathbf{C}}H_2P$), 62.51 (d, *J* = 6.76 Hz, $O\underline{\mathbf{C}}H_2CH_3$) ^{31}P NMR (175 MHz, $CDCl_3$): 24.52. HRMS (ESI$^+$): Exact mass calc. $C_5H_{14}O_4P$ [M+H] = 169.0627; Found = 169.0630.

***O,O*-Diethyl iodomethylphosphonate (7)**

To a stirred solution of tosylate **5** (644 mg, 2 mmol, 1.00 equiv) in acetone (10 mL) was added sodium iodide (600 mg, 4 mmol, 2.00 equiv) in a single portion. The resulting suspension was stirred at reflux overnight and subsequently allowed to cool. Water (20 mL) was added to the reaction mixture and the product was extracted into dichloromethane (20 mL) and washed with 2% sodium thiosulfate (10 mL). Drying of the solvent over magnesium sulfate and removal of the solvent in vacuo furnished the title compound as a pale yellow oil (485 mg, 1.82 mmol, 91%).

^{1}H NMR (400 MHz, $CDCl_3$): 1.30 (6H, t, *J* = 7.20 Hz, $OCH_2C\underline{\mathbf{H_3}}$), 2.99 (2H, d, *J* = 10.37 Hz, $IC\underline{\mathbf{H_2}}$), 4.12 (4H, overlapping doublet of quartets appearing as a quintet, J_{H-P} = J_{H-H} = 7.40 Hz, $OC\underline{\mathbf{H_2}}$). ^{13}C NMR (100 MHz, $CDCl_3$): -14.32 (d, J_{C-P} = 155.00 Hz, $\underline{\mathbf{C}}H_2I$), 16.33 (d, J_{C-P} = 5.98 Hz, $\underline{\mathbf{C}}H_3$), 63.34 (d, J_{C-P} = 6.44 Hz, $O\underline{\mathbf{C}}H_2$) ^{31}P

NMR (175 MHz, $CDCl_3$): 20.63. HRMS (ESI^+): Exact mass calc. $C_7H_{17}IO_4P$ [M+H] = 322.9904; Found = 322.9909.

***O,O*-Diethyl (trifluoromethanesulfonyl)methylphosphonate (8) [3]**

To a stirred solution of diethyl (hydroxyethyl)phosphonate (**10**, 5.04 g, 30.0 mmol, 1.00 equiv) in dichloromethane (100 mL) was added triethylamine (3.60 g, 36.0 mmol, 1.20 equiv). The mixture was cooled to −50 °C using a liquid nitrogen/methanol slush bath under nitrogen. Triflic anhydride (10.5 g, 6.4 mL, 36.0 mmol, 1.20 equiv) was added dropwise to the stirring solution and the reaction mixture was allowed to warm slowly to room temperature and was kept at this temperature for two hours. Dichloromethane (40 mL) was then added to the reaction mixture before washing with 2 M HCl (20 mL), water (20 mL) and brine (20 mL). The organic phase was dried over magnesium sulfate and the solvent removed in vacuo to furnish the title compound as a yellow oil (712 mg, 2.40 mmol, 79%).

^1H NMR (400 MHz, $CDCl_3$): 1.39 (6H, t, J = 7.08 Hz, $OCH_2C\underline{\mathbf{H_3}}$), 4.25 (H, overlapping dq appearing as a quintet, $J_{H\text{-}H} = J_{H\text{-}P}$ = 7.10 Hz, $OC\underline{\mathbf{H_2}}H_3$), 4.63 (2H, d, J = 8.85 Hz, $OC\underline{\mathbf{H_2}}P$). ^{13}C NMR (100 MHz, $CDCl_3$): 16.23 (d, $J_{C\text{-}P}$ = 5.65 Hz, $OCH_2\underline{\mathbf{C}}H_3$), 63.86 (d, $J_{C\text{-}P}$ = 6.45 Hz, $O\underline{\mathbf{C}}H_2CH_3$), 66.42 (d, $J_{C\text{-}P}$ = 168.63 Hz, $O\underline{\mathbf{C}}H_2P$), 118.58 (quartet, $J_{C\text{-}F}$ = 321.10 Hz, $F_3\underline{\mathbf{C}}\text{-}SO_2R$). ^{19}F NMR (376 MHz, $CDCl_3$): -74.13. ^{31}P NMR (175 MHz, $CDCl_3$): 12.19.

General procedure: Determination of conversion of alcohol 4 to phosphonate 6 using electrophiles 5, 7 and 8.

To a stirred suspension of magnesium *tert*-butoxide (510 mg, 3.00 mmol, 3.00 equiv) in anhydrous *N,N*-dimethylformamide (10 mL) was added alcohol **4** (179 mg, 1.00 mmol, 1.00 equiv). The resulting slurry was stirred under a nitrogen atmosphere for 45 minutes. Electrophile **5**, **7** or **8** (1.50 mmol, 1.50 equiv) was then added and the reaction mixture was heated to an internal temperature of ca. 75 °C. The reaction was allowed to proceed for 24 hours after which time it was allowed to cool to room temperature and the magnesium *tert*-butoxide was quenched with acetic acid (1.00 mL). Removal of the

solvent in vacuo followed by co-distillation with toluene (3 × 30 mL) to remove excess DMF afforded the crude reaction mixture as a sticky orange resin which was analysed by ^{1}H and ^{31}P NMR in order to determine the conversion to **6**. **NOTE:** Care should be taken at this point to minimise yield loss due to losses on transfer. The crude reaction mixture is very sticky and viscous and can be difficult to transfer manually, however it is soluble in methanol.

The crude reaction mixture was then subjected to column chromatography on silica gel (gradient elution: 0–10% MeOH in CH_2Cl_2) in order to determine the yield of **6**. **NOTE:** A wide column should be used here as the crude reaction mixture tends to precipitate, blocking the flow of solvent through the column, which can lead to a build up of pressure within the column.

*Spectral data for **6** are given below.*

***N9*-(2-Bromoethyl)adenine (12)**

To a stirred suspension of adenine (**3**, 1.35 g, 10 mmol, 1.00 equiv) and potassium carbonate (3.21 g, 23 mmol, 2.30 equiv) in *N,N*-dimethylformamide (30 mL) was added 1,2-dibromoethane (7.40 g, 3.41 mL, 40 mmol, 4.00 equiv) in a single portion. The mixture was stirred under a nitrogen atmosphere for 20 hours after which time the starting material was no longer detectable by TLC and the emergence of a new spot (R_f **12** = 0.40; eluent – 10% MeOH in CH_2Cl_2) was evident. The solvent was removed in vacuo and the residue suspended in water before isolation by vacuum filtration. Washing with cold water (20 mL) and further drying afforded 1.88 g (78%, 7.8 mmol) of the title compound as an orange solid.

Melting point: 297-299 °C. ^{1}H NMR (400 MHz, DMSO-d$_6$): 3.95 (2H, t, *J* = 5.97, C**H$_2$**Br), 4.57 (2H, t, *J* = 5.97, C**H$_2$**N), 7.27 (2H, bs, N**H$_2$**), 8.15 (1H, s, Ar**H**), 8.19 (1H, s, Ar**H**). ^{13}C NMR[†] (100 MHz, DMSO-d$_6$): 31.54 (**C**H$_2$Br), 44.61 (**C**H$_2$N), 118.65 (qAr**C**-NH$_2$), 140.87 (Ar**C**H), 149.44 (qAr**C**), 152.48 (Ar**C**H), 155.96 (qAr**C**). IR (KBr, cm^{-1}): 1385, 1357, 1326, 1305, 1246, 1072, 887, 796, 709, 646. HRMS (ESI^{+}): Exact mass calc. for

$C_7H_9N_5{}^{79}Br$ [M+H]) = 242.0041. Found = 242.0023. Exact mass calc. for $C_7H_9N_5{}^{81}Br$ [M+H] = 244.0290. Found = 244.0292.

***N9*-(2-Iodoethyl)adenine (9)**

To a stirred suspension of sodium iodide (900 mg, 6 mmol, 2.00 equiv) in acetonitrile (20 mL) was added bromide **12** (750 mg, 3 mmol, 1.00 equiv). The reaction mixture was heated to reflux and stirred for 48 hours after which time the starting material was no longer evident by TLC. The solvent was removed in vacuo and the resulting slurry was suspended in water (15 mL) and filtered. The filter cake was washed with water (2 × 10 mL) and recrystallized from methanol to furnish the title compound (766 mg, 2.44 mmol, 88%) as an off-white solid. Melting point (MeOH): 220-223 °C (Decomposition).

^{1}H NMR (400 MHz, DMSO-d_6): 3.68 (2H, t, *J* = 6.48 Hz, $CH_2C\underline{\mathbf{H_2}}I$), 4.51 (2H, t, *J* = 6.48 Hz, $C\underline{\mathbf{H_2}}CH_2I$), 7.29 (2H, bs, $N\underline{\mathbf{H_2}}$), 8.15-8.17 (2 x 1H overlapping singlets, Ar**H**). ^{13}C NMR (100 MHz, DMSO-d_6): 4.30 ($CH_2\underline{\mathbf{C}}H_2I$), 45.13 ($\underline{\mathbf{C}}H_2CH_2I$), 118.67 (Ar**C**($NH_2$)), 140.60 (Ar**C**H), 149.32 (Ar**C**), 152.45 (Ar**C**H), 155.94 (Ar**C**). IR (KBr, cm^{-1}): 3332, 3282, 2980, 2912, 1385, 1357, 1326, 1305, 1246, 1072, 512. HRMS (ESI^+): Exact mass calc. $C_7H_9N_5I$ [M+H] = 289.9903; Found = 289.9897. Anal. Calc.: C, 29.08; H, 2.79; N, 24.23. Found: C, 29.34; H, 2.81; N, 24.40.

Reaction of iodide 9 with alcohol 10 to give 6

To a stirred solution of alcohol **10** (510 mg, 3.00 mmol, 1.00 equiv) in anhydrous DMF (30 mL) was added sodium hydride (76 mg, 3.30 mmol, 1.10 equiv) portionwise under a nitrogen atmosphere at room temperature. The resulting suspension was allowed to stir for an hour before iodide **9** (870 mg, 3.00 mmol, 1.00 equiv) was added in a single portion. The reaction was allowed stir overnight for ca. 16 h before the addition of ice-water (2 mL). The solvent was removed in vacuo and the yellow residue was purified by column chromatography (0–10% MeOH in CH_2Cl_2) to afford **6** as a foamy white residue (231 mg, 0.70 mmol, 21%).

Melting point (EtOAc): 134-136 °C. (Lit: 135-136 °C) [4]

^{1}H NMR (400 MHz, DMSO-d_6): 1.14 (6H, t, *J* = 7.08 Hz, $OCH_2C\underline{\mathbf{H_3}}$ x 2), 3.84-3.95 (8H, m, $PC\underline{\mathbf{H_2}}O$, $OC\underline{\mathbf{H_2}}CH_3$ x 2, $ArC\underline{\mathbf{H_2}}CH_2O$), 4.33 (2H, t, *J* = 5.11 Hz, $ArCH_2C\underline{\mathbf{H_2}}O$), 7.22 (2H, bs, $N\underline{\mathbf{H_2}}$), 8.08 (1H, s, ArC(8)**H**), 8.14 (1H, s, ArC(2)**H**). ^{13}C NMR (100 MHz DMSO-d_6): 16.13 (d, *J* = 16.13 Hz, $OCH_2\underline{\mathbf{C}}H_3$), 42.33 ($Ar\underline{\mathbf{C}}H_2CH_2O$), 61.66 (d, *J* = 6.31 Hz, $O\underline{\mathbf{C}}H_2CH_3$), 63.67 (d, *J* = 160.69 Hz, $O\underline{\mathbf{C}}H_2P$), 70.24 (d, *J* = 11.75 Hz, $ArCH_2\underline{\mathbf{C}}H_2O$), 118.53 (Ar**C**(5)), 141.01 (Ar**C**(8)H), 149.47 (Ar**C**(4)), 152.32 (Ar**C**(2)H), 155.89 (Ar**C**(6)). ^{31}P NMR (100 MHz, DMSO-d_6): 20.88. IR (KBr, cm^{-1}): 3382, 3322, 2980, 2912, 1385, 1357, 1326, 1305, 1246, 1072. HRMS (ESI$^+$): Exact mass calc. for $C_{12}H_{21}N_5O_4P^+$ [M+H$^+$] 330.1331; Found 330.1322.

Also isolated was *O,O*-diethyl ethoxymethylphosphonate (**13**) as a pale yellow oil (108 mg, 0.55 mmol, 37%) as the least polar fraction after column chromatography.

^{1}H NMR (400 MHz, $CDCl_3$): 1.16 (3H, t, *J* = 6.84 Hz, $C\underline{\mathbf{H_3}}CH_2OCH_2P$), 1.28 (6H, t, *J* = 7.08 Hz, $OCH_2C\underline{\mathbf{H_3}}$ x 2), 3.56 (2H, d, *J* = 6.84 Hz, $CH_3CH_2OC\underline{\mathbf{H_2}}P$), 3.71 (2H, d, *J* = 8.71 Hz, $CH_3C\underline{\mathbf{H_2}}OCH_2$), 4.11 (4H, overlapping doublet of quartets appearing as a quintet, $J_{H\text{-}H} = J_{H\text{-}P}$ = 7.26 Hz, $OC\underline{\mathbf{H_2}}CH_3$ x 2). ^{13}C NMR (100 MHz, $CDCl_3$): 14.90 ($\underline{\mathbf{C}}H_3CH_2OCH_2P$), 16.57 (d, *J* = 5.57 Hz, $OCH_2\underline{\mathbf{C}}H_3$), 62.40 (d, *J* = 6.43 Hz, $CH_3\underline{\mathbf{C}}H_2OCH_2$), 64.64 (d, *J* = 167.23 Hz, $CH_3CH_2O\underline{\mathbf{C}}H_2P$), 69.11 (d, *J* = 12.47 Hz, $O\underline{\mathbf{C}}H_2CH_3$). ^{31}P NMR (175 MHz, $CDCl_3$): 22.23.

***O,O*-Diethyl 2-Chloroethoxymethylphosphonate (19) [5]**

To a two-necked 25 mL round bottom flask, equipped with a distillation condenser and a receiving vessel, was added neat triethyl phosphite (7.6 g, 8.00 mL, 48 mmol, 1.00 equiv). The triethyl phosphite was heated to an internal temperature of ca. 125–140 °C with stirring. Ether **18** (6.00 g, 48 mmol, 1.00 equiv) was added dropwise to the flask over approximately 10 minutes. The reaction was allowed to stir at this temperature for a further 4 hours, washing the sides of the flask with hexane to ensure complete consumption of both starting materials, after which time the reaction was found to be complete by ^{31}P NMR analysis. The title compound was used without further purification (11.03 g, 48 mmol, 100%).

^1H NMR (400 MHz, $CDCl_3$): 1.29 (6H, t, J = 7.33 Hz, $OCH_2C\underline{\mathbf{H_3}}$), 3.58 (2H, t, J = 5.76 Hz, $ClC\underline{\mathbf{H_2}}CH_2O$), 3.78-3.81 (4H, multiplet, $ClCH_2C\underline{\mathbf{H_2}}O$ and $OC\underline{\mathbf{H_2}}P$), 4.12 (4H, overlapping doublet of quartets appearing as a quintet, $J_{H\text{-}H}$ = $J_{H\text{-}P}$ = 7.33 Hz). ^{13}C NMR (100 MHz, $CDCl_3$): 15.47 (d, $J_{C\text{-}P}$ = 5.68 Hz, $OCH_2\underline{\mathbf{C}}H_3$), 41.44 ($Cl\underline{\mathbf{C}}H_2CH_2O$), 61.57 (d, $J_{C\text{-}P}$ = 6.51 Hz, $O\underline{\mathbf{C}}H_2CH_3$), 64.65 (d, $J_{C\text{-}P}$ = 164.00 Hz, $O\underline{\mathbf{C}}H_2P$), 72.08 (d, $J_{C\text{-}P}$ = 10.43 Hz, $ClCH_2\underline{\mathbf{C}}H_2O$). ^{31}P NMR (175 MHz, $CDCl_3$): 20.43.

***O,O*-Diethyl 2-Iodoethoxymethylphosphonate (14)**

To a stirred suspension of sodium iodide (21.00 g, 140 mmol, 2.00 equiv) in acetone (20 mL) was added chloride **19** (16.40 g, 70 mmol, 1.00 equiv) in a single portion. The resulting suspension was heated to reflux resulting in a clear yellow solution. The reaction mixture was stirred for 24 hours at this temperature during which time the reaction mixture became cloudy. After cooling the resulting suspension was filtered and the mother liquor was concentrated in vacuo. The yellow oily residue was reconstituted in chloroform (40 mL), washed with 5% sodium thiosulfate (0 mL), dried over magnesium sulfate and concentrated in vacuo to furnish the title compound (18.71 g, 58.1 mmol, 83%) as a colourless oil which turned slightly yellow on storage.

^1H NMR (400 MHz, $CDCl_3$): 1.20 (6H, t, J = 6.80 Hz, $OCH_2C\underline{\mathbf{H_3}}$), 3.14 (2H, t, J = 5.76 Hz, $IC\underline{\mathbf{H_2}}CH_2O$), 3.78-3.81 (4H, multiplet, $ClCH_2C\underline{\mathbf{H_2}}O$ and $OC\underline{\mathbf{H_2}}P$), 4.12 (4H, overlapping doublet of quartets appearing as a quintet, $J_{H\text{-}H}$ = $J_{H\text{-}P}$ = 7.33 Hz). ^{13}C NMR (100 MHz, $CDCl_3$): 0.04 ($I\underline{\mathbf{C}}H_2CH_2O$), 14.65 (d, $J_{C\text{-}P}$ = 5.74 Hz, $OCH_2\underline{\mathbf{C}}H_3$), 60.71 (d, $J_{C\text{-}P}$ = 6.50 Hz, $O\underline{\mathbf{C}}H_2CH_3$), 63.07 (d, $J_{C\text{-}P}$ = 166.37 Hz, $O\underline{\mathbf{C}}H_2P$), 71.76 (d, $J_{C\text{-}P}$ = 11.24 Hz, $ICH_2C\underline{\mathbf{H_2}}O$). ^{31}P NMR (175 MHz, $CDCl_3$): 20.44. HRMS (ESI^+): Exact mass calc. $C_7H_{17}O_4PI$ [M+H] = 322.9909; Found = 322.9915.

Alkylation of adenine (3) with 14

To a stirred solution of iodide **14** (10.00 g, 31.0 mmol, 1.05 equiv) in DMF (100 mL) was added adenine (**3**, 3.05 g, 30 mmol, 1.00 equiv) and potassium carbonate (6.00 g, 45 mmol, 1.50 equiv) in a single portion. The reaction was allowed to stir at room temperature for 30 hours until complete consumption of adenine was evident by TLC (R_f = 0.00, 10% MeOH in CH_2Cl_2). The reaction mixture was filtered by gravity and the filtrate was concentrated in vacuo to yield a yellow oily residue which was then codistilled with toluene (3 × 30 mL) to remove the remaining traces of DMF. Purification of the resulting residue by column chromatography (R_f (**6**) = 0.48; Gradient 0–10% MeOH in CH_2Cl_2) furnished (6.90 g, 22.00 mmol, 70%) of the title compound as a colourless foam.

Spectral data consistent with those reported above.

Also present in the crude reaction mixture was the *N*7-regioisomer (**17**). Based on integration of the NC**H₂** signals in the ^{1}H NMR spectrum of the crude reaction mixture at 4.33 ppm (**6**) and 4.50 ppm (**17**), it is formed in a ratio of 4:1 (*N*9:*N*7). Phosphonate **17** was isolated as a colourless solid (1.34 g, 5 mmol, 16%) after column chromatography (R_f (**19**) = 0.22; Gradient 0–10% MeOH in CH_2Cl_2).

Melting point (MeOH): > 250 °C.

^{1}H NMR (400 MHz, DMSO-d_6): 1.12 (6H, t, *J* = 7.04 Hz, $OCH_2C\underline{\mathbf{H_3}}$ x 2), 3.79-3.92 (6H, m, containing $O\underline{\mathbf{CH_2}}$... $OC\underline{\mathbf{H_2}}CH_3$ x 2 and $OC\underline{\mathbf{H_2}}P$), 3.96 (2H, t, *J* = 4.61 Hz, $ArCH_2C\underline{\mathbf{H_2}}O$), 4.50 (2H, t, *J* = 4.61 Hz, $ArC\underline{\mathbf{H_2}}CH_2O$) 7.78 (1H, s, ArC(2)$\underline{\mathbf{H}}$), 7.88 (bs, one of $N\underline{\mathbf{H_2}}$), 7.93 (bs, one of $N\underline{\mathbf{H_2}}$), 8.17 (1H, s, ArC(8)$\underline{\mathbf{H}}$). ^{13}C NMR (100 MHz, DMSO-d_6): 16.13 (d, *J* = 5.56 Hz, $OCH_2\underline{\mathbf{C}}H_3$), 48.56 ($Ar\underline{\mathbf{C}}H_2CH_3O$), 61.65 (d, *J* = 6.72 Hz, $O\underline{\mathbf{C}}H_2CH_3$), 63.72 (d, *J* = 162.03 Hz, $O\underline{\mathbf{C}}H_2P$), 69.13 (d, *J* = 11.61 Hz, $ArCH_2\underline{\mathbf{C}}H_2O$), 120.36 (Ar$\underline{\mathbf{C}}$(5)), 143.75 (Ar$\underline{\mathbf{C}}$(8)H), 149.48 (Ar$\underline{\mathbf{C}}$(4)), 152.34 (Ar$\underline{\mathbf{C}}$(2)H), 155.00 (Ar$\underline{\mathbf{C}}$(6)). ^{31}P NMR (175 MHz, DMSO-d_6): 20.79 IR (KBr, cm^{-1}): 3382, 3322, 2980, 2912, 1385, 1357, 1326, 1305, 1246, 1072. HRMS (ESI$^+$): Exact mass calc. for $C_{12}H_{20}N_5O_4P^+$

$[M+H]^+$ = 330.1331; Found 330.1328. Anal. Calc.: C, 43.77; H, 6.12; N, 21.27; Found C, 44.08; H, 6.23; N, 21.47.

General procedure: Determination of the regioselectivity of the alkylation of adenine with 14

To a stirred solution of iodide **14** (330 mg, 1.05 mmol, 1.05 equiv) in *N,N*-dimethylformamide (10 mL) was added adenine (135 mg, 1.00 mmol, 1.00 equiv) and the base (1.50 mmol, 1.50 equiv). The reaction mixture was stirred at room temperature or heated/cooled to the required temperature for 30 hours. The reaction mixture was filtered by gravity and the filtrate was concentrated in vacuo to yield a yellow oily residue which was then codistilled with toluene (3 × 20 mL) to remove the remaining traces of DMF. The crude reaction mixture was then analysed by ^{1}H and ^{31}P NMR. Based on integration of the NC$\underline{\mathbf{H_2}}$ signals in the ^{1}H NMR spectrum of the crude reaction mixture at 4.33 ppm (**6**) and 4.50 ppm (**20**) the ratio of the two regioisomers was determined.

Salt 21

To a stirred suspension of adenine (**3**, 1.38 g, 10 mmol, 1.00 equiv) in dichloromethane (30 mL) was added aqueous tetrabutylammonium hydroxide (6.48 g of 1.51 M solution, equivalent to 2.59 g tetrabutylammonium hydroxide in 3.89 g water, 10 mmol, 1.00 equiv). The resulting cloudy mixture was allowed to stir for 20 minutes. Removal of the solvent in vacuo afforded an off-white residue which was azeotropically dried with toluene to afford the title compound as an off-white powder (3.76 g, 10 mmol, 100%).

Melting point (Toluene): 163-167 °C

^{1}H NMR (400 MHz, $CDCl_3$): 0.92 (12H, t, *J* = 7.11 Hz, $NCH_2CH_2CH_2C\underline{\mathbf{H_3}}$), 1.21-1.38 (24H, m, containing $NCH_2C\underline{\mathbf{H_2}}CH_2CH_3$ and $NCH_2CH_2C\underline{\mathbf{H_2}}CH_3$), 2.92 (8H, m, $NC\underline{\mathbf{H_2}}CH_2CH_2CH_3$), 7.30 (2H, s, ArN$\underline{\mathbf{H_2}}$), 7.91 (1H, s, ArC(8)$\underline{\mathbf{H}}$), 8.19 (1H, s, ArC(2)$\underline{\mathbf{H}}$). ^{13}C NMR (100 MHz, $CDCl_3$): 13.61 ($NCH_2CH_2CH_2\underline{\mathbf{C}}H_3$), 19.57 ($NCH_2CH_2\underline{\mathbf{C}}H_2CH_3$), 23.80 ($NCH_2\underline{\mathbf{C}}H_2CH_2CH_3$), 58.40 ($N\underline{\mathbf{C}}H_2CH_2CH_2CH_3$), 118.12 (Ar$\underline{\mathbf{C}}$(5)), 149.67 (Ar$\underline{\mathbf{C}}$(8)H),

149.98 (Ar**C**(4)), 153.27 (Ar**C**(2)H), 160.57 (Ar**C**(6)). IR (ATR, cm^{-1}): 3340, 3240, 2958, 2873, 1626, 1590, 1526, 1473, 1372, 1189, 1066, 879, 803, 701, 658, 581, 546. Elemental analysis: **20** requires C (66.98%), H (10.71%) N (22.32%); Found = C (67.31%), H (10.99%) and N (22.04%).

Preparation of 6 via alkylation of salt 21 with iodide 14

To a stirred solution of **21** (376 mg, 1.00 mmol, 1.00 equiv) in acetonitrile (10 mL) was added iodide **14** (332 mg, 1.00 mmol, 1.00 equiv) in a single portion. The resulting solution was stirred at room temperature for 30 hours after which time the solvent was removed in vacuo. The resulting yellow residue was purified by column chromatography (0–10% MeOH in DCM) affording compound **6** as a colourless solid (211 mg, 0.68 mmol, 68%).

*Spectroscopic data for **6** were consistent with those reported above.*

Synthesis of *O,O*-diethyl ((2-(6-chloro-9*H*-purin-9-yl)ethoxy)methyl)phosphonate (24) and *O,O*-diethyl ((2-(6-chloro-7*H*-purin-7-yl)ethoxy)methyl)phosphonate (23) from iodide 14.

To a stirred solution of iodide **14** (177 mg, 0.55 mmol, 1.05 equiv) in DMF (1 mL) was added 6-chloropurine (**22**, 77 mg, 0.50 mmol, 1.00 equiv) and potassium carbonate (100 mg, 0.75 mmol, 1.50 equiv) in a single portion. The reaction was allowed to stir at room temperature for 24 hours until complete consumption of **19** was evident by TLC (R_f = 0.00; 10% MeOH in CH_2Cl_2). The reaction mixture was filtered by gravity and the filtrate was concentrated in vacuo to yield a yellow oily residue which was then co-distilled with toluene (3 × 30 mL) to remove the remaining traces of DMF. Purification by column chromatography afforded **23** and **24**.

Major product 24

Isolated as the less polar product fraction (R_f = 0.41; 10% MeOH in DCM) (118 mg, 0.34 mmol, 68%) as a colourless foam.

^{1}H NMR (400 MHz, DMSO-d$_6$): 1.11 (6H, t, *J* = 7.10 Hz, OCH$_2$C**H$_3$** x 2), 3.85-3.96 (8H, m, containing OC**H$_2$**P, OC**H$_2$**CH$_3$ x 2, ArCH$_2$C**H$_2$**O), 4.51 (2H, t, *J* = 4.90 Hz, ArC**H$_2$**CH$_2$O), 8.67 (1H, s, ArC(8)**H**), 8.80 (1H, s, ArC(2)**H**). ^{13}C NMR (100 MHz, DMSO-d$_6$): 16.07 (d, *J* = 5.74 Hz, OCH$_2$**C**H$_3$), 43.27 (Ar**C**H$_2$CH$_2$O), 61.59 (d, *J* = 6.30 Hz, O**C**H$_2$CH$_3$), 63.61 (d, *J* = 161.53 Hz, O**C**H$_2$P), 69.80 (d, *J* = 11.31 Hz, ArCH$_2$**C**H$_2$O), 130.67 (Ar**C**(5)), 147.70 (Ar**C**(8)H), 148.88 (Ar**C**(4)), 151.45 (Ar**C**(2)H), 151.96 (Ar**C**(6)). ^{31}P NMR (175 MHz, DMSO-d$_6$): 20.88. IR (ATR, cm^{-1}): 2984, 2907, 1633, 1590, 1562, 1425, 1395, 1335, 1240, 1121, 1025, 971, 875, 830, 857, 637, 475. HRMS (ESI$^+$): Exact mass calc. for $C_{12}H_{19}ClN_4O_4P^+$ [M+H]$^+$ = 349.0827; Found = 349.0811.

Minor product 23:

Isolated as the more polar product fraction (R_f = 0.36; 10% MeOH in DCM) (15 mg, 0.045 mmol, 9%) as a colourless foam.

^{1}H NMR (400 MHz, DMSO-d$_6$): 1.10 (6H, t, *J* = 7.04 Hz, OCH$_2$C**H$_3$** x 2), 3.70-3.95 (8H, m, containing OC**H$_2$**P, OC**H$_2$**CH$_3$ x 2, ArCH$_2$C**H$_2$**O), 4.69 (2H, t, *J* = 5.00 Hz, ArC**H$_2$**CH$_2$O), 8.75 (1H, s, ArC(8)**H**), 8.81 (1H, s, ArC(2)**H**). ^{13}C NMR (100 MHz, DMSO-d$_6$): 16.11 (d, *J* = 5.72 Hz, OCH$_2$**C**H$_3$), 46.14 (Ar**C**H$_2$CH$_2$O), 61.55 (d, *J* = 6.19 Hz, O**C**H$_2$CH$_3$), 63.74 (d, *J* = 159.73 Hz, O**C**H$_2$P), 70.84 (d, *J* = 10.59 Hz, Ar**C**H$_2$CH$_2$O), 122.03 (Ar**C**(5)), 142.19 (Ar**C**(4)), 151.38 (Ar**C**H), 151.46 (Ar**C**H), 161.58 (Ar**C**(6)). ^{31}P (175 MHz, DMSO-d$_6$): 20.91. IR (ATR, cm^{-1}): 2982, 2929, 1634, 1587, 1564, 1544, 1454, 1422, 1392, 1355, 1236, 1122, 1049, 1025, 97, 829, 643, 466. HRMS (ESI$^+$): Exact mass calc. for $C_{12}H_{19}ClN_4O_4P^+$ [M+H]$^+$ = 349.0827; Found = 349.0815.

Synthesis of *N*9-[2-(diethoxyphosphorylmethoxy)ethyl]adenine (6) from *O,O*-diethyl ((2-(6-chloro-9*H*-purin-9-yl)ethoxy)methyl)phosphonate (24)

A stirred solution of phosphonate **24** (100 mg, 0.28 mmol, 1.00 equiv) in 7 M methanolic ammonia (1 mL) was heated to reflux for six hours after which time, the starting material was no longer evident by TLC (R_f = 0.41; 10% MeOH in DCM). Removal of the solvent in vacuo yielded a yellow residue which was purified by column chromatography to give **6** as a colourless solid (75 mg, 0.23 mmol, 81%).

Synthesis of *O,O*-diethyl ((2-(6-(benzylamino)-9*H*-purin-9-yl)ethoxy)methyl)phosphonate (27) and *O,O*-diethyl ((2-(6-(benzylamino)-7*H*-purin-9-yl)ethoxy)methyl)phosphonate (26) from iodide 14.

To a stirred solution of iodide **14** (177 mg, 0.55 mmol, 1.05 equiv) in DMF (1 mL) was added *N*6-benzyladenine (**25**) [6] (112 mg, 0.50 mmol, 1.00 equiv) and potassium carbonate (100 mg, 0.75 mmol, 1.50 equiv) in a single portion. The reaction was allowed to stir at room temperature for 24 hours until complete consumption of **25** was evident by TLC (R_f = 0.19, 10% MeOH in CH_2Cl_2). The reaction mixture was filtered by gravity and the filtrate was concentrated in vacuo to yield a yellow oily residue which was then co-distilled with toluene (3 × 30 mL) to remove the remaining traces of DMF. Purification by column chromatography (0–10% MeOH in DCM) afforded **26** and **27**.

Major product 27:

Isolated as the less polar product following column chromatography (147 mg, 35 mmol, 70%)

^{1}H NMR (400 MHz, DMSO-d$_6$): 1.12 (6H, t, *J* = 6.91 Hz, OCH$_2$C**H$_3$** x 2), 3.84-3.93 (8H, m, containing ArCH$_2$C**H$_2$**O, OC**H$_2$**P and OC**H$_2$**CH$_3$ x 2), 4.35 (2H, t, *J* = 4.80 Hz, ArC**H$_2$**CH$_2$O), 4.70 (2H, bs, NHC**H$_2$**Ph), 7.20 (2H, t, *J* = 7.37 Hz, *m*-ArC**H** x 2), 7.26-7.35 (3H, m, containing *o*-ArC**H** x 2 and *p*-ArC**H**), 8.12 ppm (1H, s, ArC(8)**H**), 8.20 (1H, s, ArC(2)**H**), 8.35 (1H, bs, N**H**CH$_2$Ph).^{13}C NMR (100 MHz, DMSO-d$_6$): 16.16 (d, *J* = 5.71 Hz, OCH$_2$**C**H$_3$), 42.36 (Ar**C**H$_2$CH$_2$O), 42.78 (NH**C**H$_2$Ph), 61.64 (d, *J* = 6.25 Hz, O**C**H$_2$CH$_3$), 63.64 (d, *J* = 164.62 Hz, O**C**H$_2$P), 70.27 (d, *J* = 12.00 Hz, ArCH$_2$**C**H$_2$O), 118.88 (Ar**C**(5)), 126.52 (*p*-Ar**C**H), 127.06 (*m*-Ar**C**H), 128.12 (*o*-Ar**C**H), 140.46 (Ar**C**(4)), 140.99 (Ar**C**(8)H), 148.89 (Ar**C**(2)H), 152.26 (Ar**C**(6)), 154.28 (*i*-Ar**C**). ^{31}P NMR (175 MHz, DMSO-d$_6$): 21.38. IR (ATR, cm^{-1}): 3278, 2982, 2915, 1617, 1581, 1482, 1452, 1243, 1120, 1026, 970, 648. HRMS (ESI$^+$): Exact mass calc. for $C_{19}H_{27}N_5O_4P^+$ = 420.1795; Found = 420.1799.

Minor product 26:

Isolated as the more polar product following column chromatography (6 mg, 0.015 mmol, 3%). Due to restricted rotation, two rotameric forms are observable by ^{1}H NMR in a ratio of 0.3:0.7 – designated as minor and major respectively.

^{1}H NMR (600 MHz, DMSO-d$_6$): 1.07-1.11 (6H, m, overlapping $OCH_2C\underline{\mathbf{H_3}}$ x 2 (major) and $OCH_2C\underline{\mathbf{H_3}}$ x 2 (minor)), 3.82-3.87 (4H, m, overlapping $OC\underline{\mathbf{H_2}}CH_3$ x 2(major) and $OC\underline{\mathbf{H_2}}CH_3$ x 2(minor)), 3.95-3.98 (2H, m, overlapping $ArCH_2C\underline{\mathbf{H_2}}O$ (major) and $ArCH_2C\underline{\mathbf{H_2}}O$ (minor)), 4.48-4.51 (2H, m overlapping $ArC\underline{\mathbf{H_2}}CH_2O$ (major) and $ArC\underline{\mathbf{H_2}}CH_2O$ (minor)), 4.75 (bs, $NHC\underline{\mathbf{H_2}}Ph$ (major)), 5.32 (d, *J* = 7.17 Hz, $NHC\underline{\mathbf{H_2}}Ph$ (minor)), 7.22 (1H, t, *J* = 7.25 Hz, overlapping *p*-ArC<u>**H**</u> (major) and (minor)), 7.30 (2H, t, *J* = 7.80 Hz, overlapping *m*-ArC<u>**H**</u> (major) and (minor)), 7.35 (d, *J* = 7.80 Hz, *o*-ArC<u>**H**</u> (major)), 7.38 (d, *J* = 7.48 Hz, *o*-ArC<u>**H**</u> (minor)), 7.78 (1H, s, ArC(2)<u>**H**</u> (major) and (minor)), 8.23 (1H, s, ArC(8)<u>**H**</u> (minor)), 8.33 (1H. s. ArC(8)<u>**H**</u> (major)), 8.73 (t, *J* = 7.17 Hz, $N\underline{\mathbf{H}}CH_2Ph$ (minor)), 8.98 (1H, bs, $N\underline{\mathbf{H}}CH_2Ph$ (major)). ^{13}C NMR (125 MHz, DMSO-d$_6$): 16.52 (d, *J* = 5.08 Hz, $OCH_2\underline{\mathbf{C}}H_3$ (major) and (minor)), 45.83 ($Ar\underline{\mathbf{C}}H_2CH_2O$ (major)), 46.06 ($Ar\underline{\mathbf{C}}H_2CH_2O$ (minor)), 48.95 ($NH\underline{\mathbf{C}}H_2Ph$ (minor)), 49.16 ($NH\underline{\mathbf{C}}H_2Ph$ (major)), 62.16 (d, *J* = 6.32 Hz, $O\underline{\mathbf{C}}H_2CH_3$ (major) and (minor)), 64.26 (d, *J* = 162.03 Hz, $O\underline{\mathbf{C}}H_2P$ (major) and (minor)) 69.61 (d, *J* = 11.85 Hz, $ArCH_2\underline{\mathbf{C}}H_2O$ (major) and (minor)), 121.48 (Ar<u>**C**</u> (major)), 127.25 (Ar<u>**C**</u> (minor)), 127.65 (ArC (major)), 127.84 (Ar<u>**C**</u> (minor)), 128.35 (Ar<u>**C**</u> (major)), 128.56 (Ar<u>**C**</u> (minor)), 128.72 (Ar<u>**C**</u> (major) and (minor)), 139.97 (Ar<u>**C**</u> (major)), 140.32 (Ar<u>**C**</u> (minor)), 143.88 (Ar<u>**C**</u> (major)), 144.35 (Ar<u>**C**</u> (minor)), 149.36 (Ar<u>**C**</u> (major)), 151.29 (Ar<u>**C**</u> (minor)), 152.75 (Ar<u>**C**</u> (major)), 152.94 (Ar<u>**C**</u> (minor)), 153.66 (Ar<u>**C**</u> (major)), 154.21 (Ar<u>**C**</u> (minor)). ^{31}P NMR (225 MHz, DMSO-d$_6$): 21.74. IR (ATR, cm^{-1}): 3278, 2982, 2915, 1617, 1581, 1482, 1452, 1243, 1120, 1026, 970, 648. HRMS (ESI$^+$): Exact mass calc. for $C_{19}H_{27}N_5O_4P^+$ $[M+H]^+$ = 420.1795; Found = 420.1798.

Synthesis of *N*9-[2-(diethoxyphosphorylmethoxy)ethyl]adenine (6) from *O,O*-diethyl ((2-(6-(benzylamino)-9*H*-purin-9-yl)ethoxy)methyl)phosphonate (27)

Phosphonate **27** (105 mg, 0.25 mmol, 1.00 equiv), 5% Pd/C (10 mg) and a magnetic stirring bar were added to a 25 mL round-bottomed flask. The flask was evacuated a refilled three times with hydrogen from a latex balloon before ethanol (3 mL) was added. The resulting black suspension was stirred at room temperature for 24 hours before being filtered through a pad of Celite© and concentrated in vacuo. The resulting colourless solid was purified by column chromatography giving **6** as a colourless solid (72 mg, 0.22 mmol, 89%).

Synthesis of *O,O*-diethyl ((2-(6-(((dimethylamino)methylene)amino)-7*H*-purin-7-yl)ethoxy)methyl)phosphonate (29)

To a stirred solution of iodide **14** (177 mg, 0.55 mmol, 1.05 equiv) in DMF (1 mL) was added purine [7] **28** (95 mg, 0.50 mmol, 1.00 equiv) and potassium carbonate (100 mg, 0.75 mmol, 1.50 equiv) in a single portion. The reaction was allowed to stir at room temperature for 24 hours until complete consumption of **28** was evident by TLC (R_f = 0.00, 10% MeOH in CH_2Cl_2). The reaction mixture was filtered by gravity and the filtrate was concentrated in vacuo to yield a yellow oily residue which was then co-distilled with toluene (3 × 30 mL) to remove the remaining traces of DMF. Purification by column chromatography (R_f = 0.44, 10% MeOH in DCM) afforded **29** (151 mg, 0.39 mmol, 79%) as a colourless solid.

^{1}H NMR (400 MHz, DMSO-d$_6$): 1.12 (6H, t, J = 6.95 Hz, $OCH_2C\underline{\mathbf{H_3}}$ x 2), 3.08 (3H, s, one of $N(C\underline{\mathbf{H_3}})_2$), 3.21 (3H, s, one of $N(C\underline{\mathbf{H_3}})_2$), 3.82-3.95 (8H, m, containing $OC\underline{\mathbf{H_2}}CH_3$ x 2, $OC\underline{\mathbf{H_2}}P$ and $ArCH_2C\underline{\mathbf{H_2}}O$), 4.68 (2H, t, J = 4.72 Hz, $ArC\underline{\mathbf{H_2}}CH_2O$), 8.31 (1H, s, ArC(8)$\underline{\mathbf{H}}$), 8.42 (1H, s, ArC(2)$\underline{\mathbf{H}}$), 8.90 (1H, s, N=C-$\underline{\mathbf{H}}$). ^{13}C NMR (100 MHz, DMSO-d$_6$): 16.13 (d, J = $OCH_2\underline{\mathbf{C}}H_3$), 34.67 (one of $N(\underline{\mathbf{C}}H_3)_2$), 40.64 (one of $N(\underline{\mathbf{C}}H_3)_2$), 46.12 ($Ar\underline{\mathbf{C}}H_2CH_2O$), 61.63 (d, J = 6.29 Hz, $O\underline{\mathbf{C}}H_2CH_3$), 63.64 (d, J = 162.75 Hz, $O\underline{\mathbf{C}}H_2P$), 71.52 (d, J = 11.52 Hz, $ArCH_2\underline{\mathbf{C}}H_2O$), 116.55 (Ar$\underline{\mathbf{C}}$(5)), 147.11 (Ar$\underline{\mathbf{C}}$(8)H), 151.76 (Ar$\underline{\mathbf{C}}$(2)H), 154.49 (Ar$\underline{\mathbf{C}}$(4)), 156.46 (N=$\underline{\mathbf{C}}$-H), 160.79 (Ar$\underline{\mathbf{C}}$(6)). ^{31}P

(175 MHz, DMSO-d_6): 20.92. IR (ATR, cm^{-1}): 2983, 2930, 1633, 1584, 1563, 1543, 1498, 1454, 1421, 1353, 1235, 1118, 1047, 1022, 970, 874, 807, 637, 622. HRMS (ESI$^+$): Exact mass calc. for $C_{15}H_{26}N_6O_4P^+$ $[M+H]^+$ = 385.1748; Found = 385.1755.

Synthesis of 2-chloroethoxymethylphosphonic acid (31)

To a stirred solution of phosphonate ester **19** (5.52 g, 24 mmol, 1.00 equiv) in acetonitrile (110 mL) was added trimethylsilyl bromide (15.10 g, 96 mmol, 4.00 equiv) in a single portion. The reaction was stirred under a blanket of nitrogen overnight, after which time the solution became pale brown. The reaction mixture was then concentrated in vacuo and co-distillation with acetonitrile (50 mL) and methanol (4 × 30 mL) furnished the title compound (4.18 g, 24 mmol, 100%) as a colourless oil, which was used without further purification. Note the colourless oil developed a slight brown colour on standing.

^{1}H NMR (400 MHz, DMSO-d_6): 3.60 (2H, d, J = 8.59 Hz, OC**<u>H</u>**$_2$P), 3.72-3.81 (4H, m, containing ClCH$_2$C**<u>H</u>**$_2$O and ClC**<u>H</u>**$_2$CH$_2$O). ^{13}C NMR (100 MHz, DMSO-d_6): 43.15 (Cl**<u>C</u>**H$_2$CH$_2$O), 66.28 (d, J = 160.87 Hz, O**<u>C</u>**H$_2$P), 72.09 (d, J = 10.59 Hz, ClCH$_2$**<u>C</u>**H$_2$O). ^{31}P NMR (175 MHz, DMSO-d_6): 16.60. IR (ATR, cm^{-1}): 3388, 2885, 2300, 1634, 1113, 1005, 945, 666, 527. HRMS (ESI$^-$): Exact mass calc. for $C_3H_7ClO_4P^-$$[M-H]^-$ = 172.9776; Found = 172.9743.

Synthesis of iodomethyl pivalate (32)

To a stirred solution of chloromethyl pivalate (2.50 g, 17 mmol, 1.00 equiv) in acetonitrile (5 mL) was added sodium iodide (4.50 g, 30 mmol, 1.90 equiv) in a single portion. The reaction mixture was allowed to stir under a nitrogen atmosphere overnight at 30 °C, during which time a strong brown colour had formed. Reaction progress was followed by ^{1}H NMR by taking aliquots from the reaction and removing the solvent in vacuo. Dichloromethane (25 mL) and water (25 mL) were subsequently added to the reaction mixture. The dichloromethane layer was separated from the aqueous layer and then washed

with 2% sodium thiosulfate solution (20 mL) before drying over anhydrous magnesium sulfate. Removal of the solvent in vacuo afforded the title compound as a yellow-pink oil (3.46 g, 14.5 mmol, 85%).

Spectroscopic data were consistent with those reported previously in the literature [8]

^{1}H NMR (300 MHz, $CDCl_3$): 1.19 (9H, s, C(C**H_3**)$_3$), 5.93 (2H, s, OC**H_2**I). ^{13}C NMR (75 MHz, $CDCl_3$): 26.74 (C(**C**H_3)$_3$), 31.37 (O**C**H_2I), 38.9 (**C**(CH_3)$_3$), 176.27 (**C**=O).

Synthesis of *O,O*-di(pivaloxymethyl) 2-chloroethoxymethylphosphonate (33)

To a stirred solution of phosphonic acid **31** (860 mg, 5.00 mmol, 1.00 equiv) in acetonitrile (50 mL) was added DBU (1.90 g, 12.50 mmol, 2.50 equiv) in a single portion. The resulting solution was stirred at room temperature for 30 minutes. Pivalate **32** (3.02 g, 12.50 mmol, 2.50 equiv) was then added and the resulting solution was stirred for 48 h at room temperature. Removal of the solvent in vacuo afforded a yellow residue which was purified by column chromatography (eluent: 100% diethyl ether) giving the title compound as a yellow oil (1.08 g, 2.7 mmol, 54%).

^{1}H NMR (400 MHz, $CDCl_3$): 1.24 (18H, s, 2 x C(C**H_3**)$_3$), 3.63 (2H, t, *J* = 6.98 Hz, ClC**H_2**CH_2O), 3.86 (2H, t, *J* = 6.98 Hz, ClCH_2C**H_2**O), 3.95 (2H, d, *J* = 8.99 Hz, OC**H_2**P), 5.69-5.75 (4H, m, OC**H_2**O). ^{13}C NMR (100 MHz, $CDCl_3$): 26.81 (C(**C**H_3)$_3$). 38.71 (Cl**C**H_2CH_2O), 42.27 (**C**(CH_3)$_3$), 65.53 (d, *J* = 165.61 Hz, O**C**H_2P), 73.11 (d, *J* = 9.63 Hz, ClCH_2**C**H_2O), 81.69 (d, *J* = 6.31 Hz, O**C**H_2O), 176.80 (**C**=O). ^{31}P NMR (175 MHz, $CDCl_3$): 20.70. IR (ATR, cm^{-1}): 2974, 2936, 2912, 2875, 1750, 1481, 1462, 1429, 1275, 1130, 1055, 998, 958, 890, 767, 564. HRMS (ESI$^+$): Exact mass calc. for $C_{15}H_{29}ClNO_8P^+$ [M+H]$^+$ = 403.1283; Found = 403.1289.

((2-Oxido-1,4,2-dioxaphosphinan-2-yl)oxy)methyl pivalate (35)

To a stirred solution of phosphonate **33** (201 mg, 0.50 mmol, 1.00 equiv) in MeCN (5 mL) in a microwave vial was added DBU (153 mg, 1.00 mmol, 2.00 equiv) and a magnetic stirrer bar. The vial was sealed with a Teflon cap and heated with stirring to 120 °C in a CEM Discovery SP Microwave Synthesizer. The

reaction mixture was held at this temperature for 20 minutes and then allowed to cool gradually. The contents were then diluted with DCM (20 mL) and washed with 2 M HCl (2 × 10 mL) and brine (1 × 10 mL) before being dried over magnesium sulfate and concentrated in vacuo affording the title compound as a pale yellow oil (110 mg, 0.44 mmol, 88%).

^{1}H NMR (400 MHz, $CDCl_3$): 1.25 (9H, s, C(C**H₃**)₃), 3.70-3.74 (1H, m, one of C(6)**H**), 3.80-3.83 (1H, m, one of C(6)**H**), 3.95 (1H, d, *J* = 14.74 Hz, one of C(2)**H**), 4.11-4.17 (1H, m one of C(2)**H**), 4.30-4.38 (1H, m, one of C(5)**H**), 4.52-4.58 (1H, m, one of C(5)**H**), 5.70-5.78 (2H, m, OC**H₂**O). ^{13}C NMR (100 MHz, $CDCl_3$): 26.85 (C(**C**H₃)₃), 38.77 (**C**(CH₃)₃), 65.09 (d, *J* = 145.73 Hz, **C**(4)), 66.62 (d, *J* = 6.36 Hz, **C**(5)), 71.44 (d, *J* = 8.86 Hz, **C**(6)H), 81.45 (d, *J* = 6.05 Hz, O**C**H₂O), 176.57 (**C**=O). ^{31}P NMR (175 MHz, $CDCl_3$): 11.75. IR (ATR, cm^{-1}): 2972, 2932, 2873, 1748, 1691, 1611, 1481, 1256, 1135, 1120, 1094, 1049, 989, 931, 852, 593. HRMS (ESI^+): Exact mass calc. for $C_9H_{18}O_6P^+$ $[M+H]^+$ = 253.0836; Found = 253.0839.

O,O-Diethyl Iodomethylphosphonate

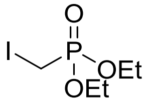

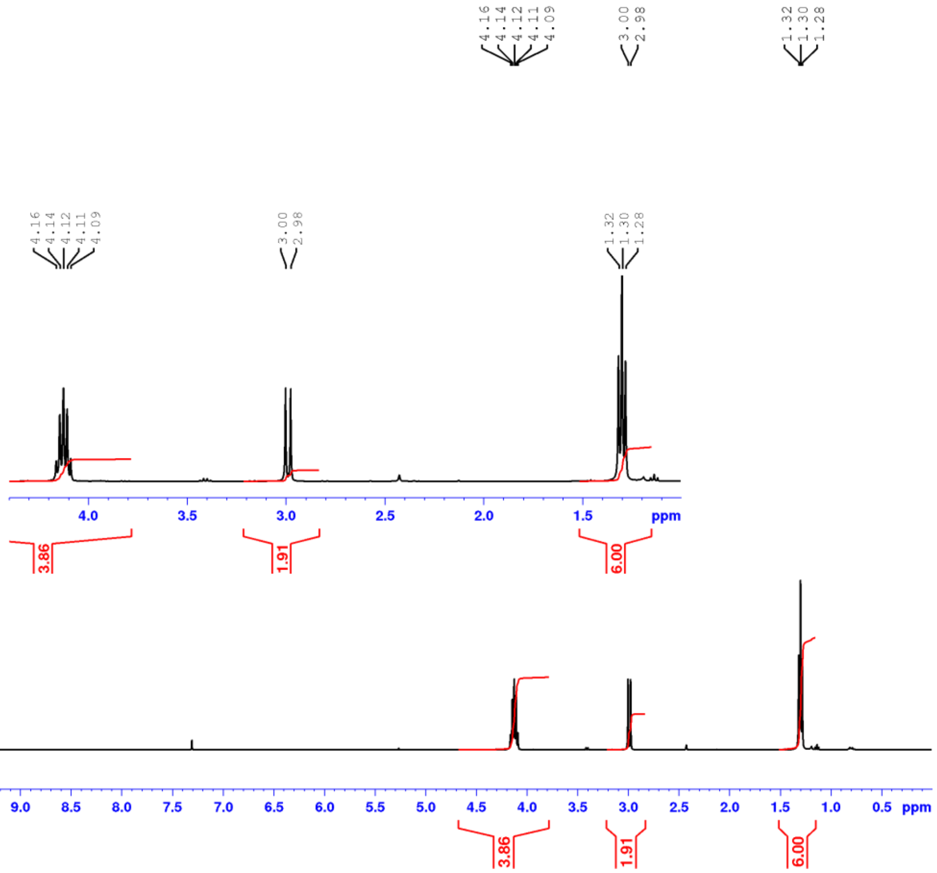

O,O-Diethyl Iodomethylphosphonate

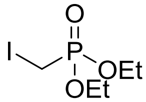

O,O-diethyl iodomethylphosphonate

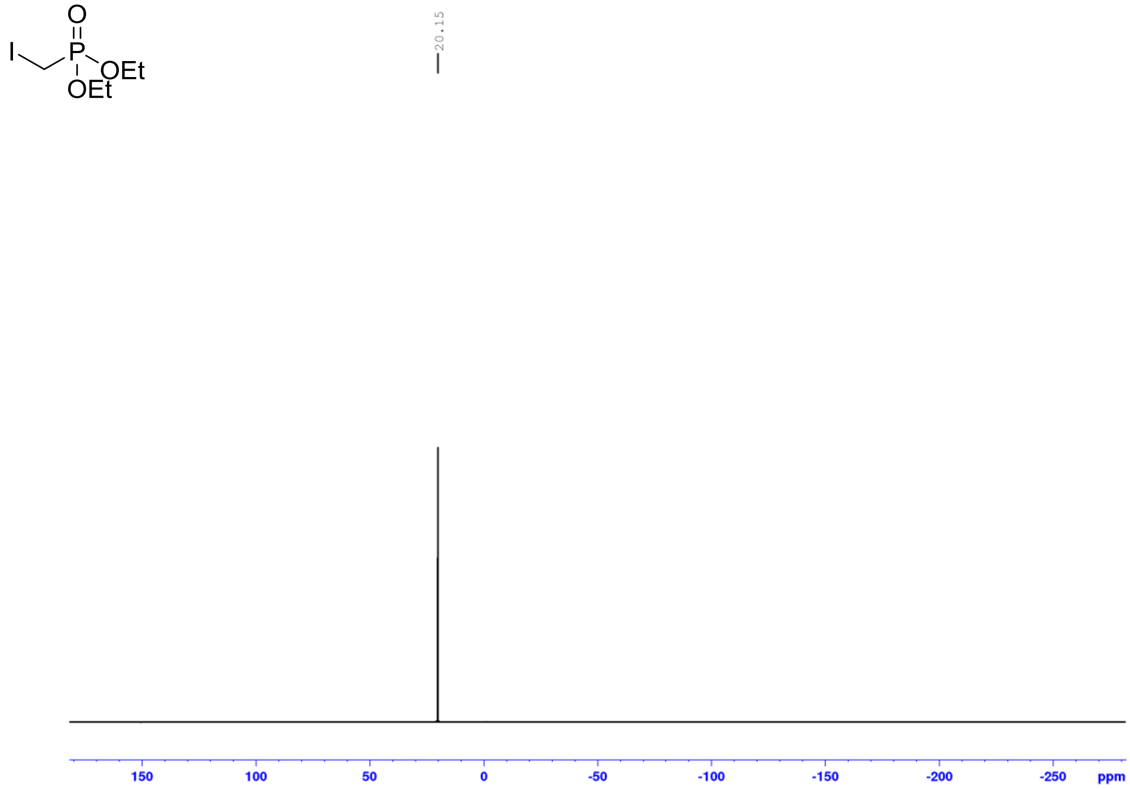

O,O-diethyl hydroxymethylphosphonate

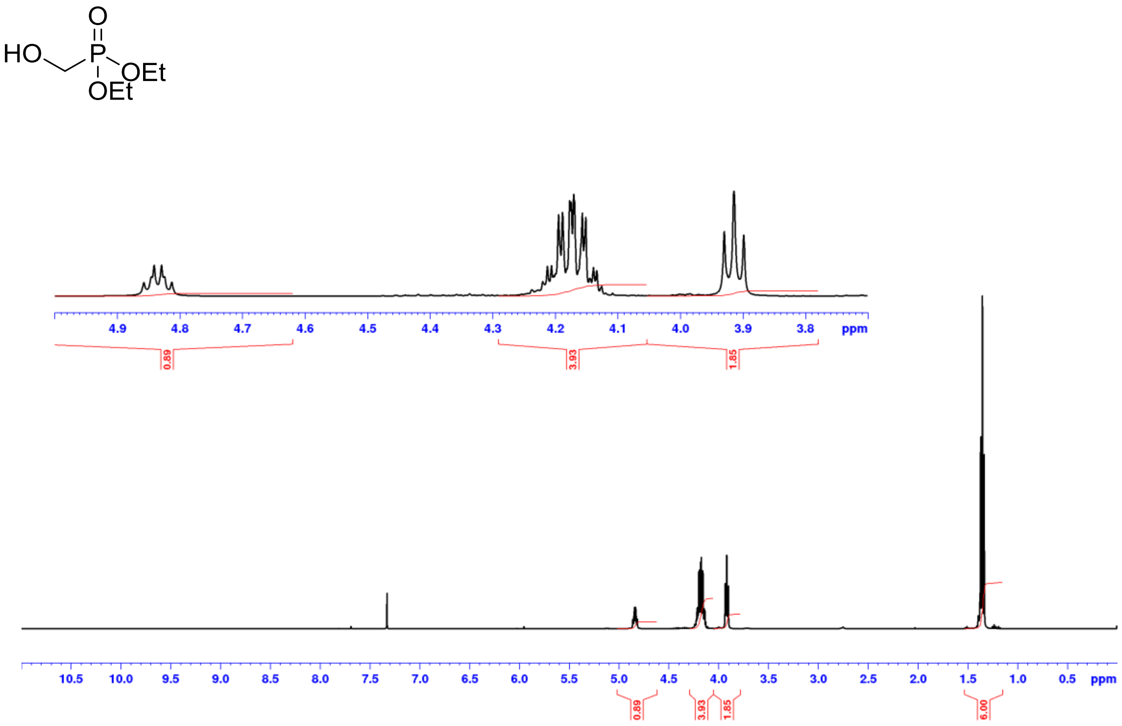

O,O-Diethyl Hydroxymethylphosphonate

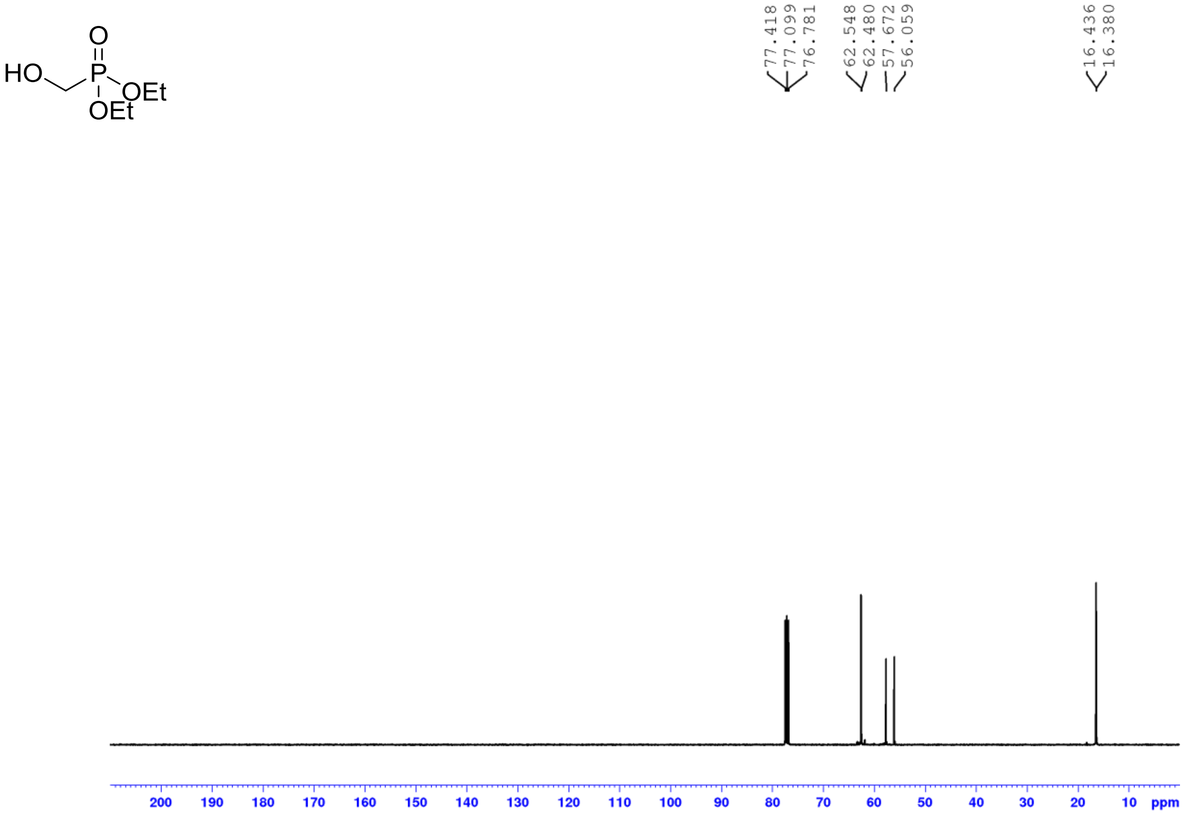

O,O-diethyl hydroxymethylphosphonate

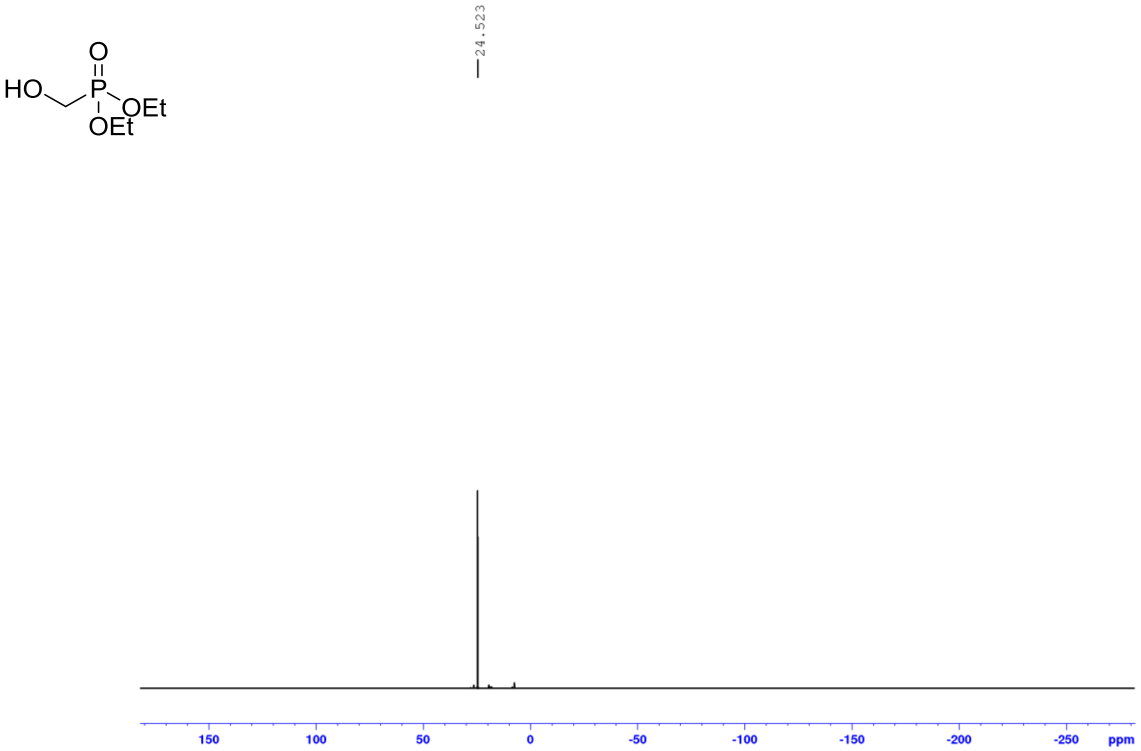

N9-(2-bromoethyl)adenine

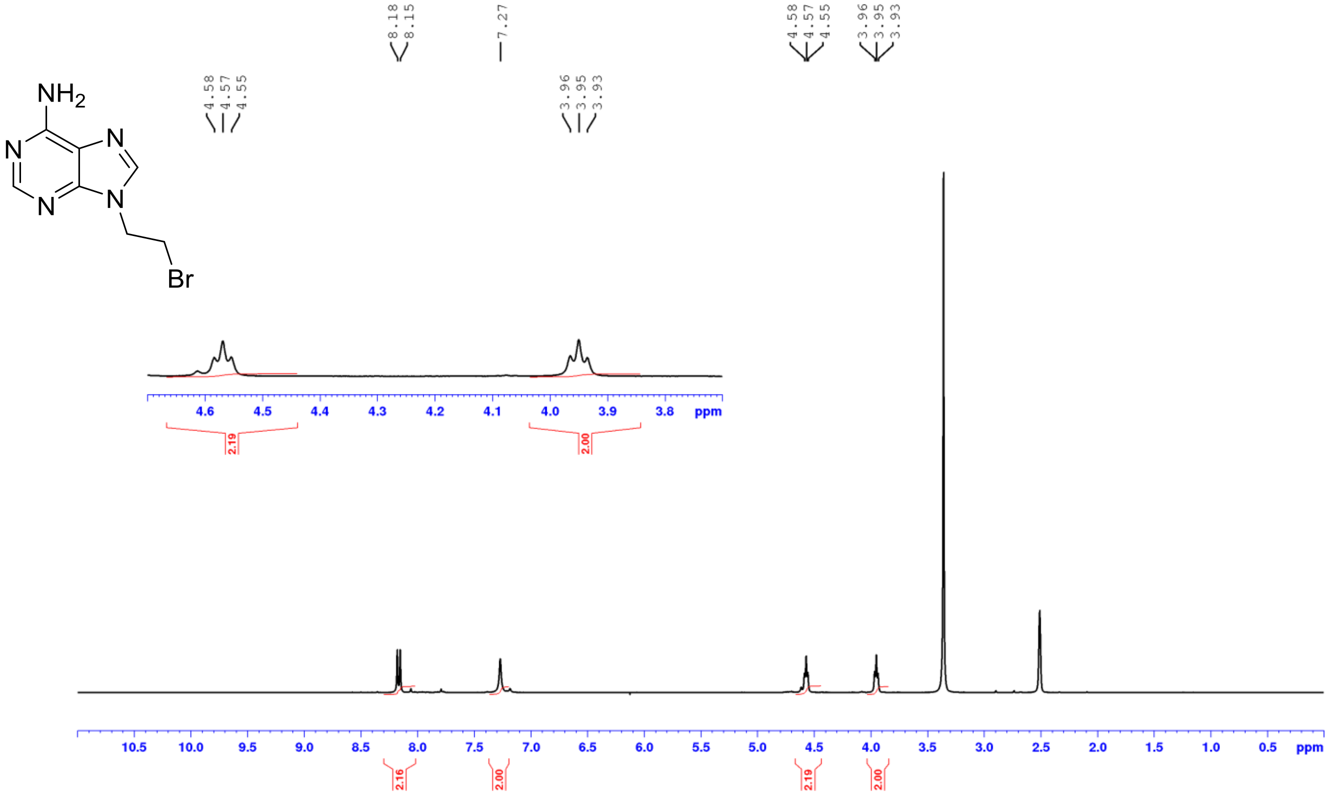

N9-(2-Bromoethyl)adenine

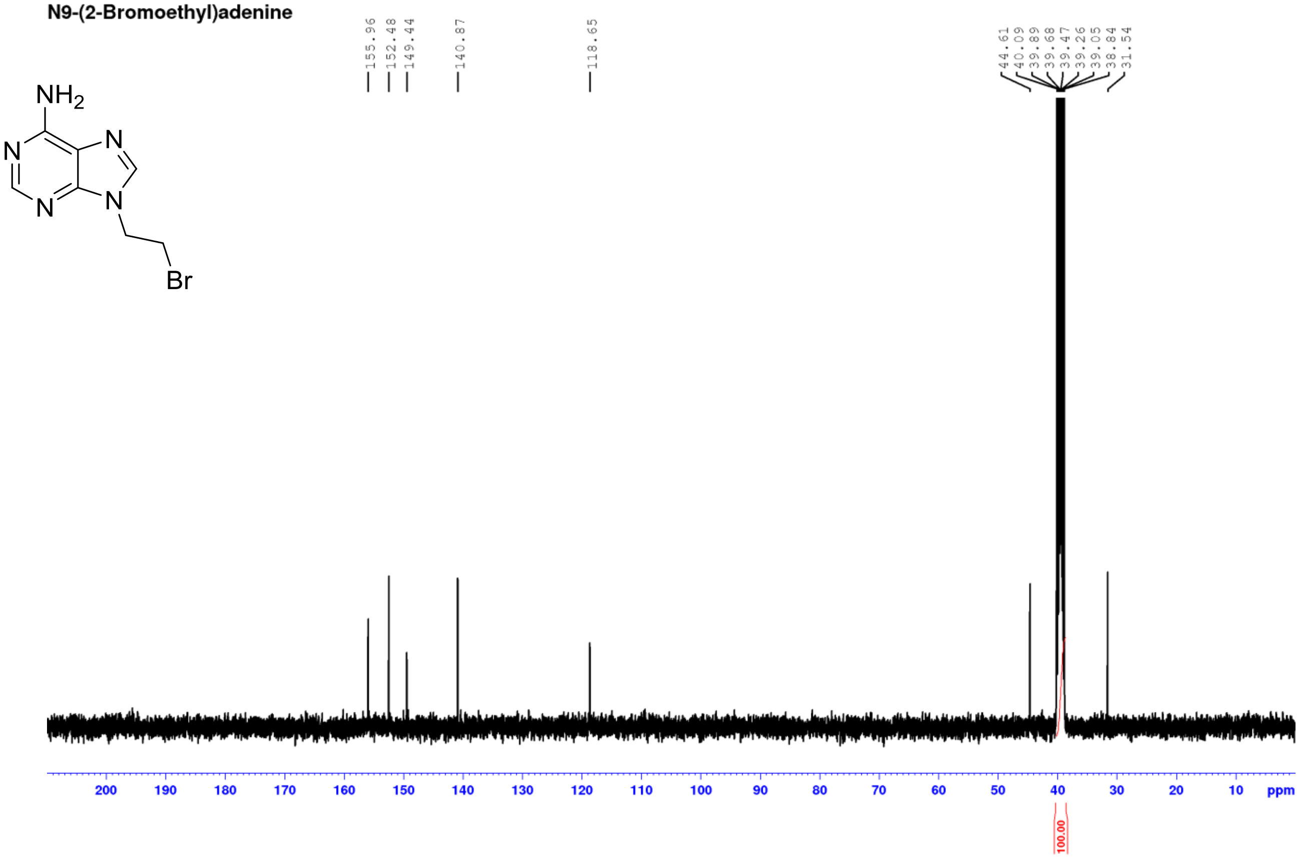

N9-(2-iodoethyl)adenine

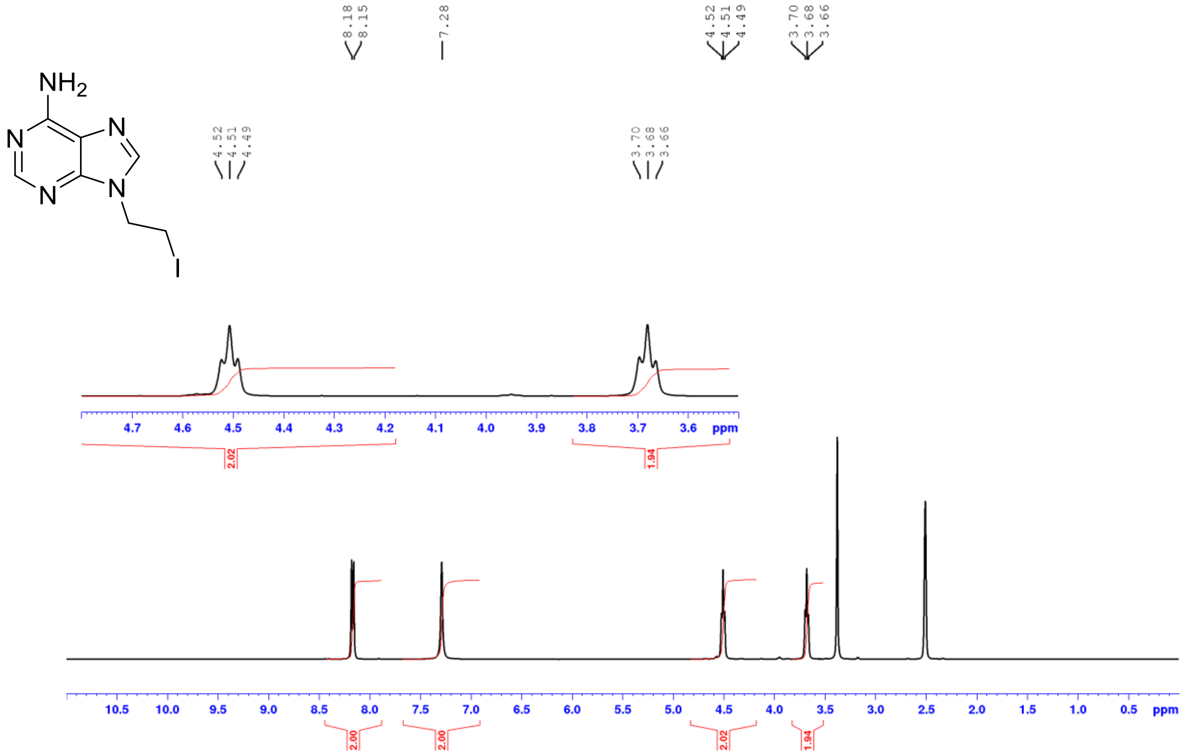

N9-(2-iodoethyl)adenine

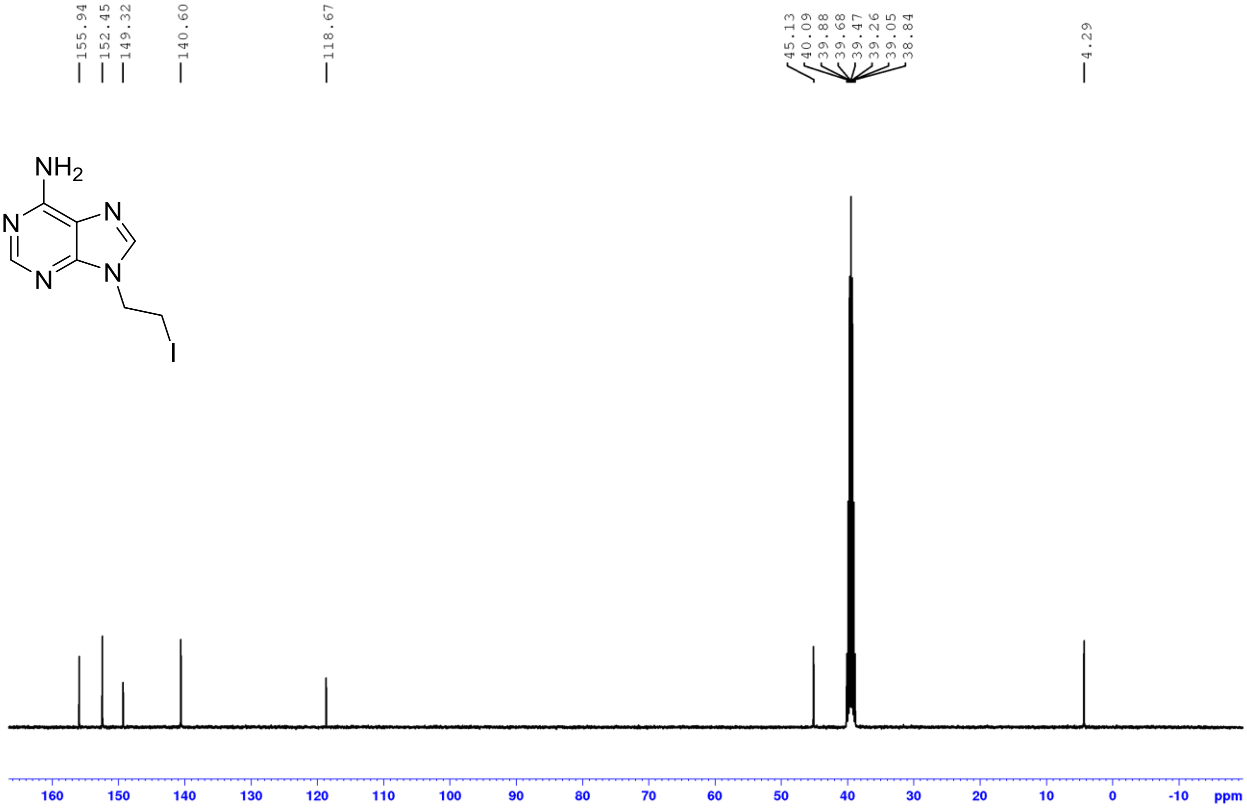

O,O-Diethyl Ethoxymethylphosphonate
(By product)

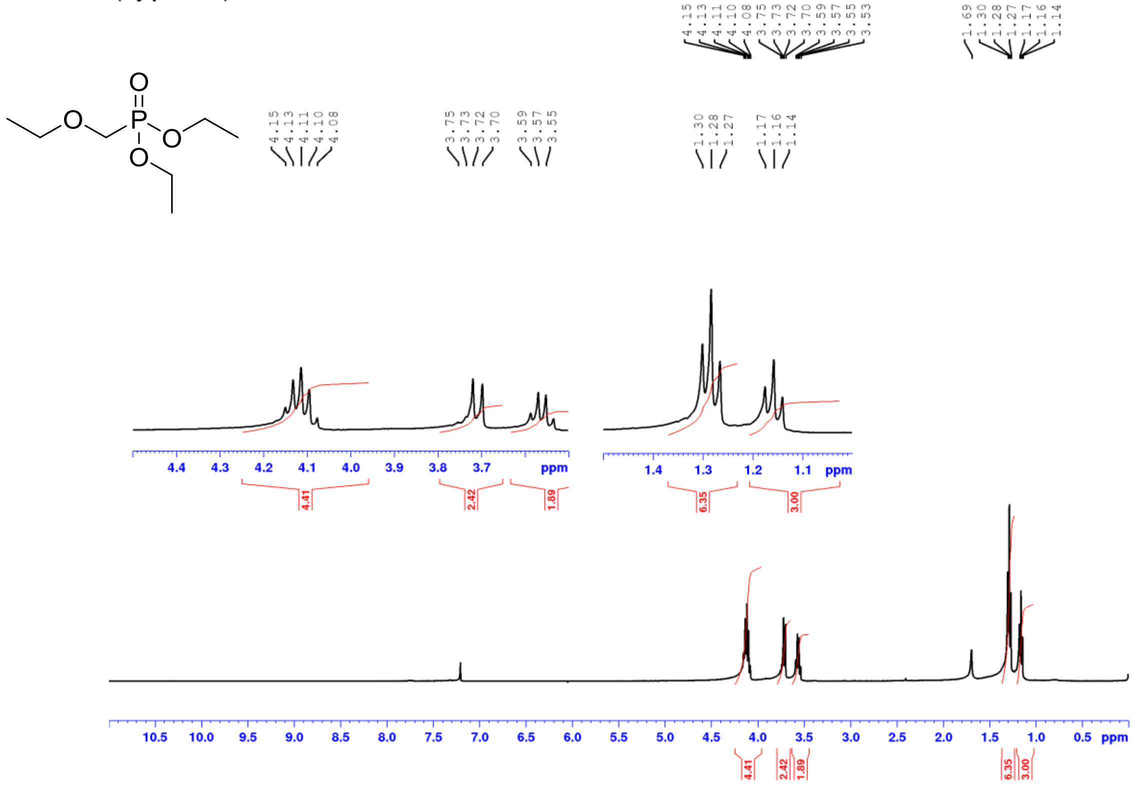

O,O-Diethyl Ethoxymethylphosphonate
(By Product)

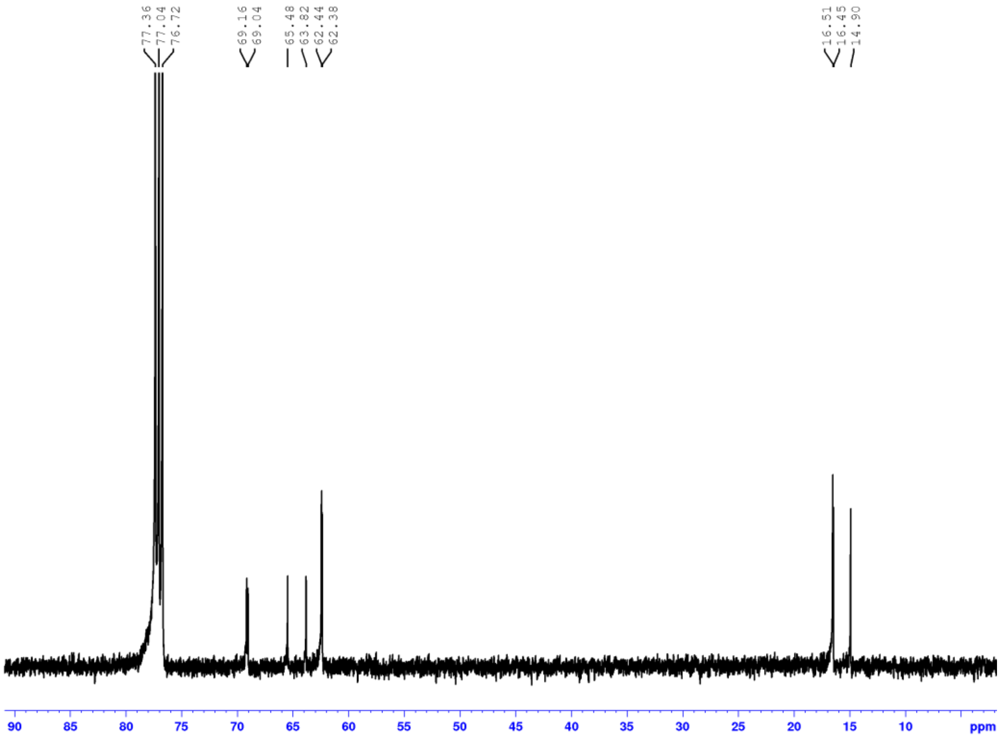

O,O-Diethyl Ethoxymethylphosphonate
(By Product)

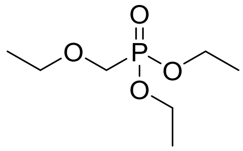

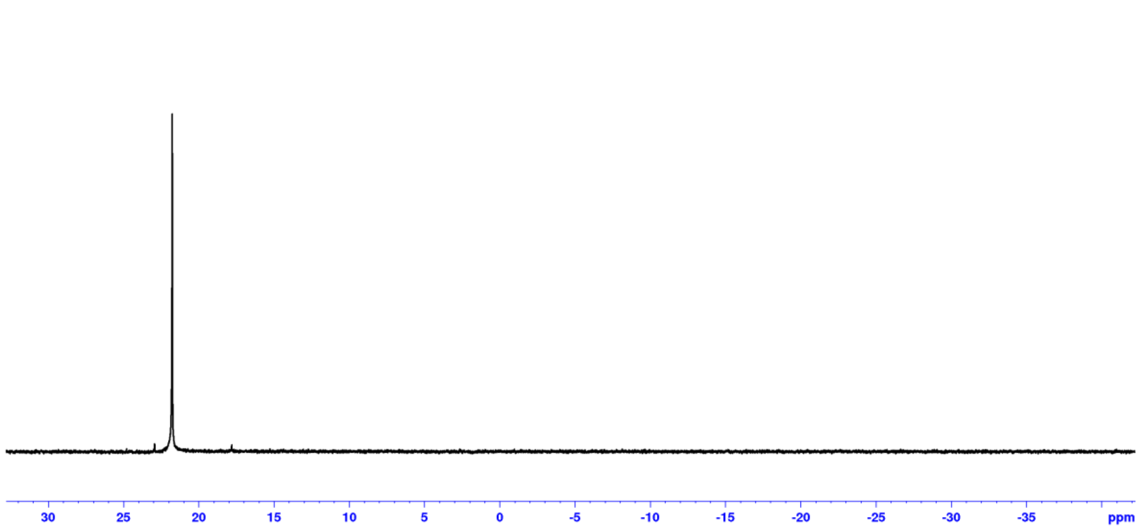

O,O-Diethyl (2-Chloroethoxy)methylphosphonate

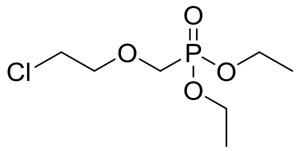

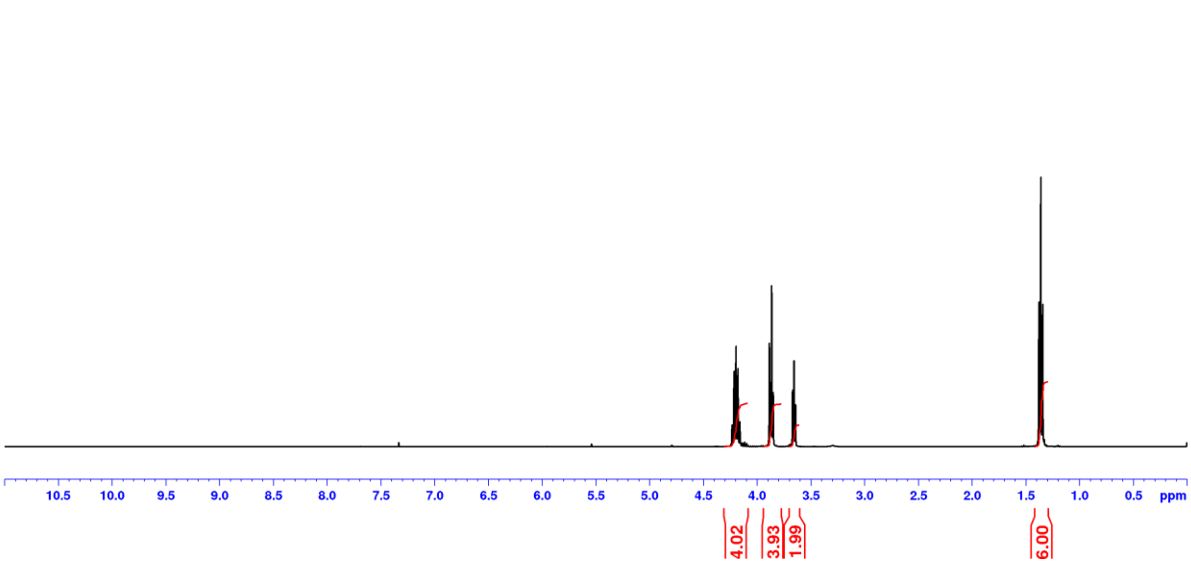

O,O-Diethyl (2-Chloroethoxy)methylphosphonate

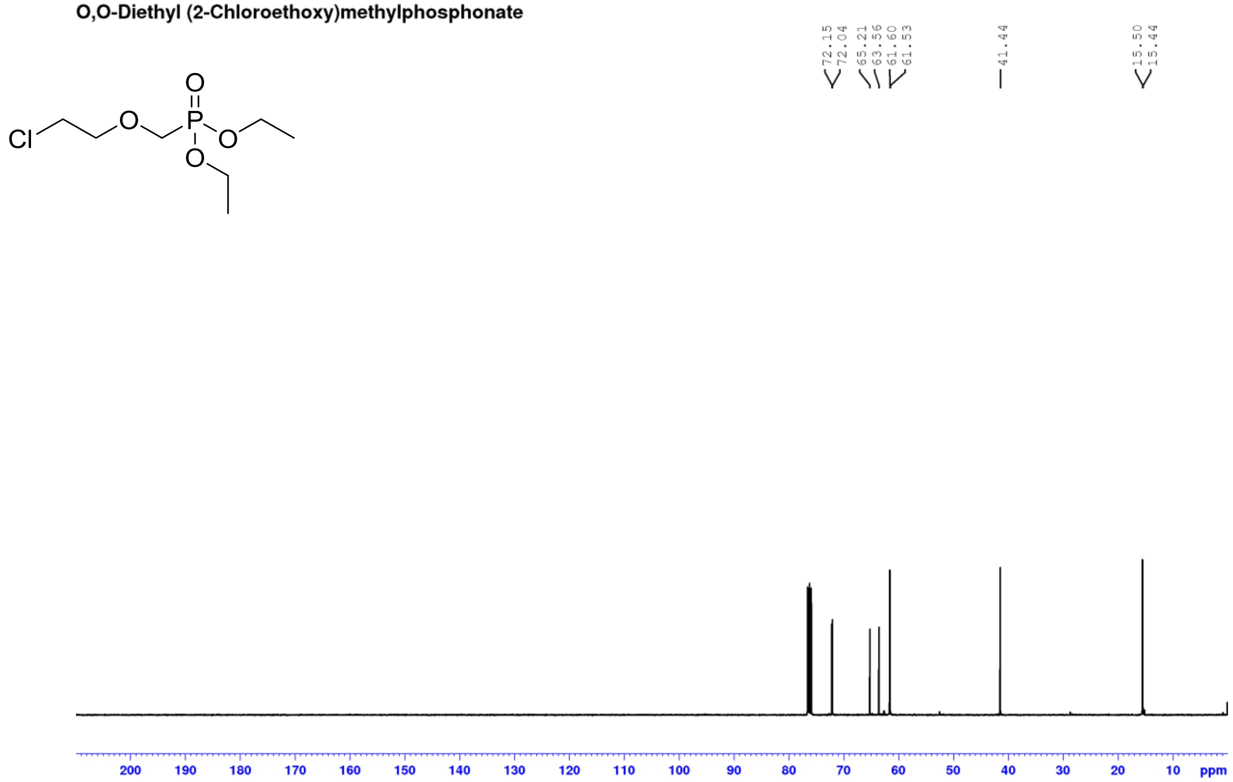

O,O-Diethyl (2-chloroethoxy)methylphosphonate

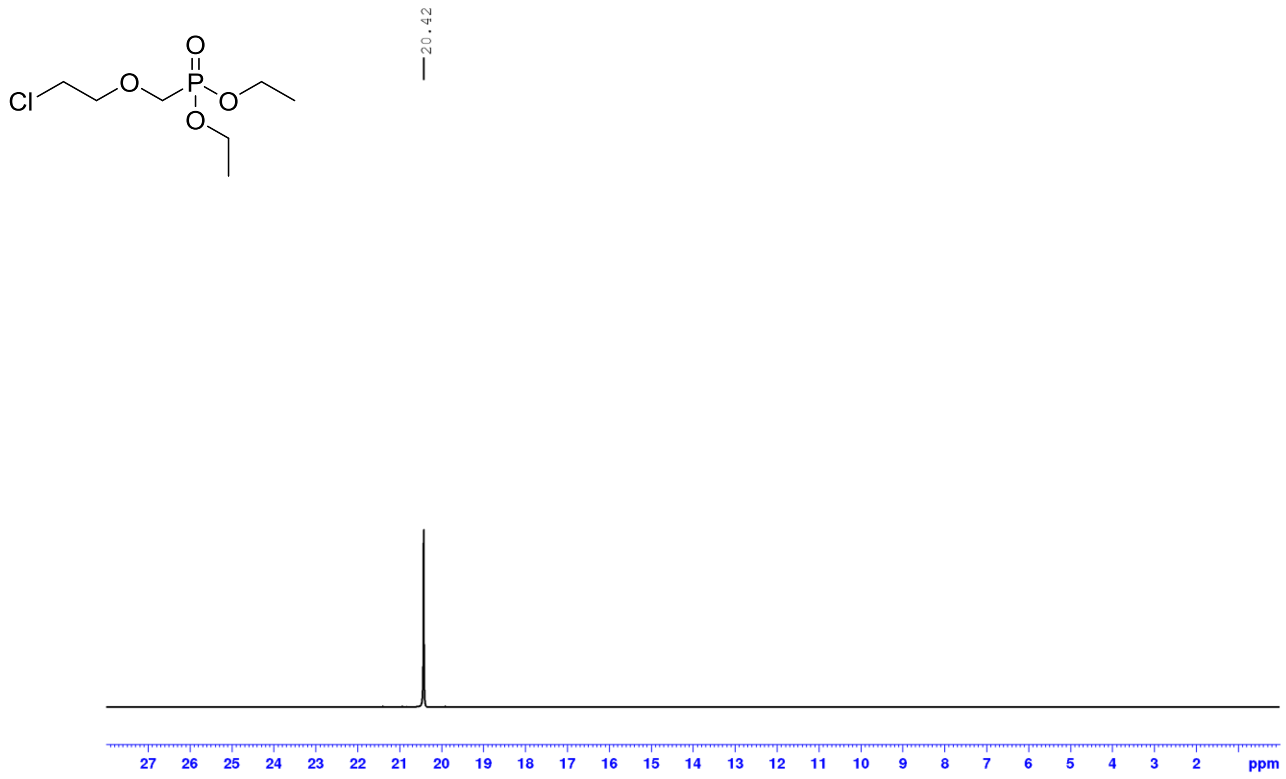

O,O-diethyl (2-iodoethoxy)methylphosphonate

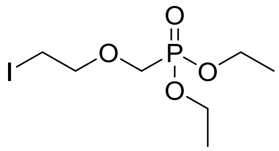

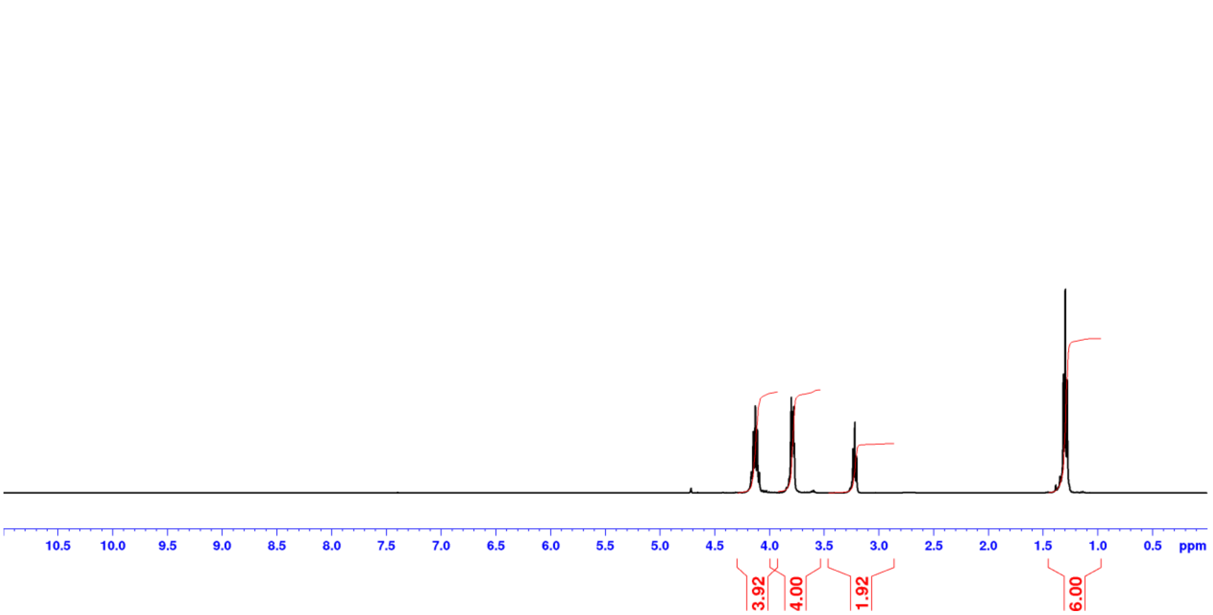

O,O-diethyl (2-iodoethoxy)methylphosphonate

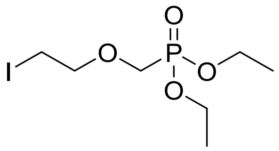

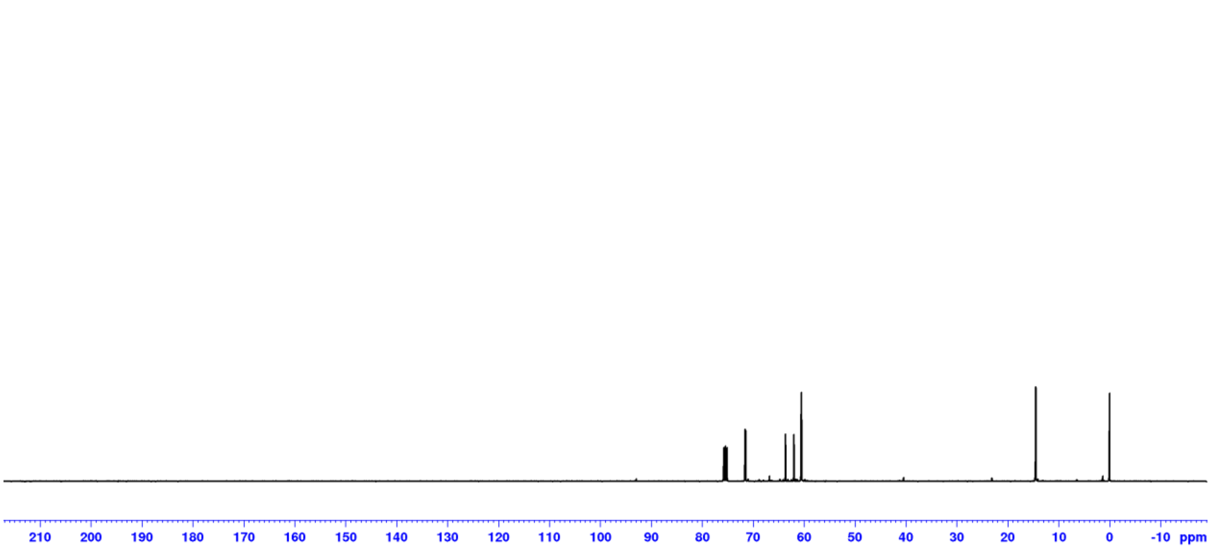

O,O-Diethyl (2-Iodoethoxy)methylphosphonate

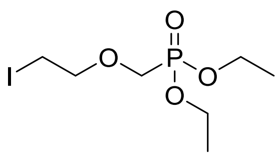

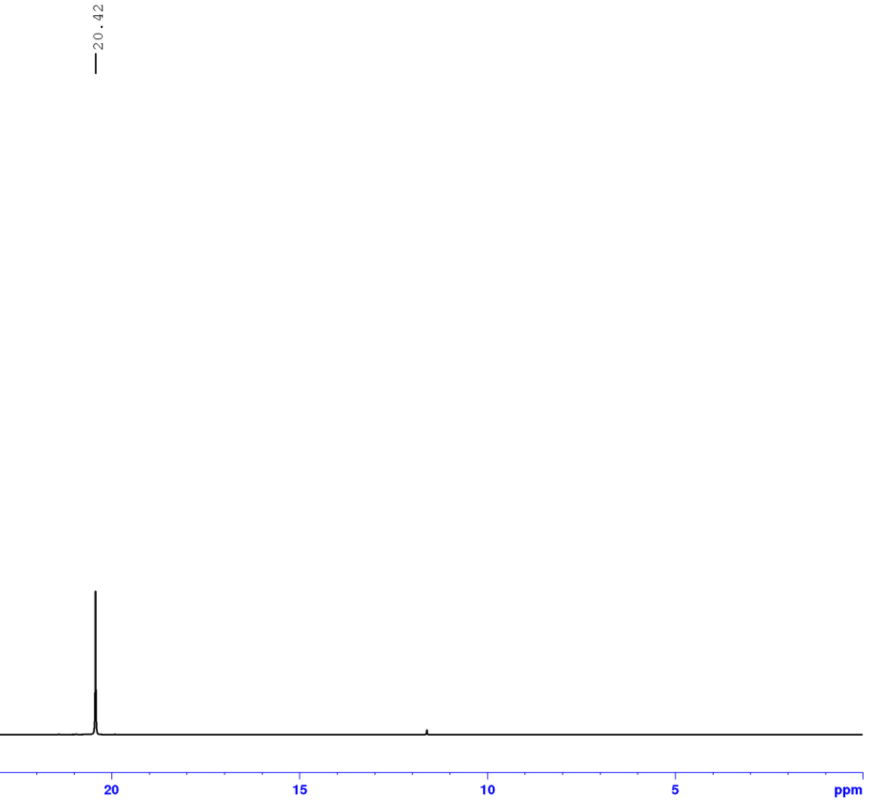

O,O-Diethyl N9-PMEA

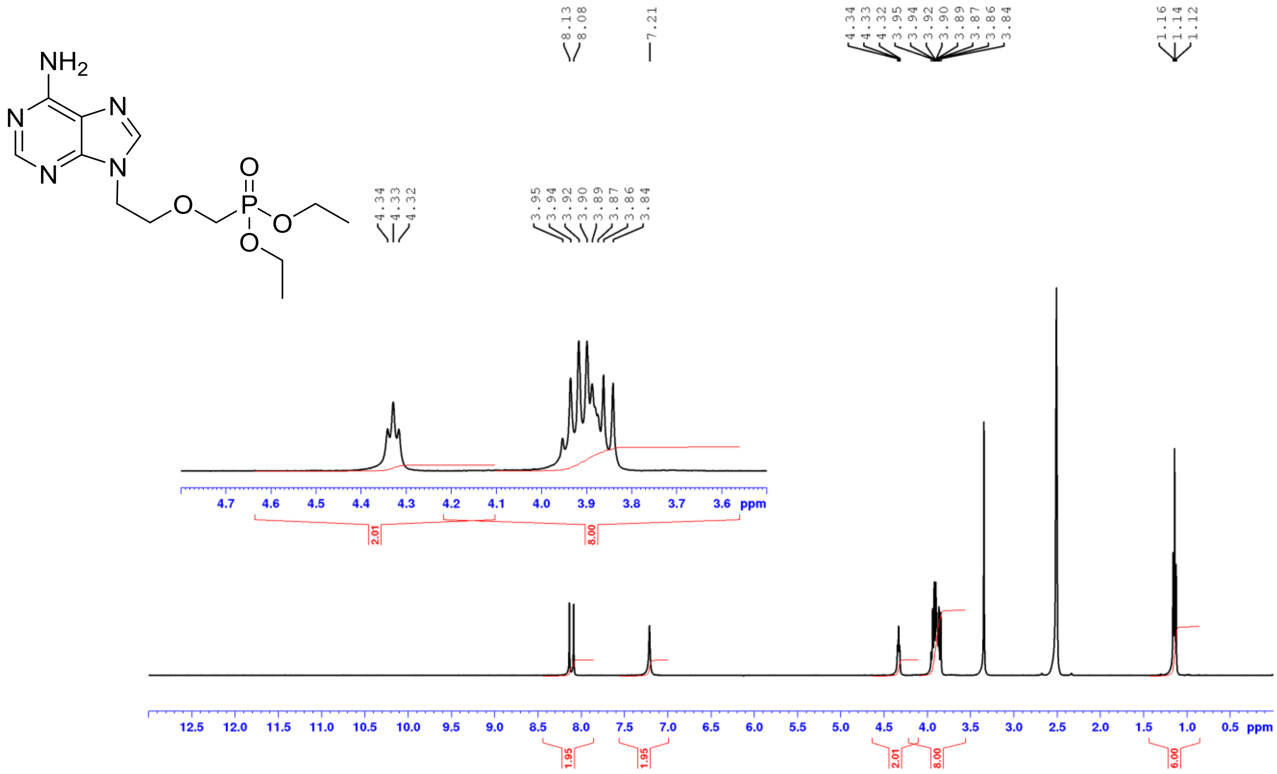

O,O-Diethyl N9-PMEA

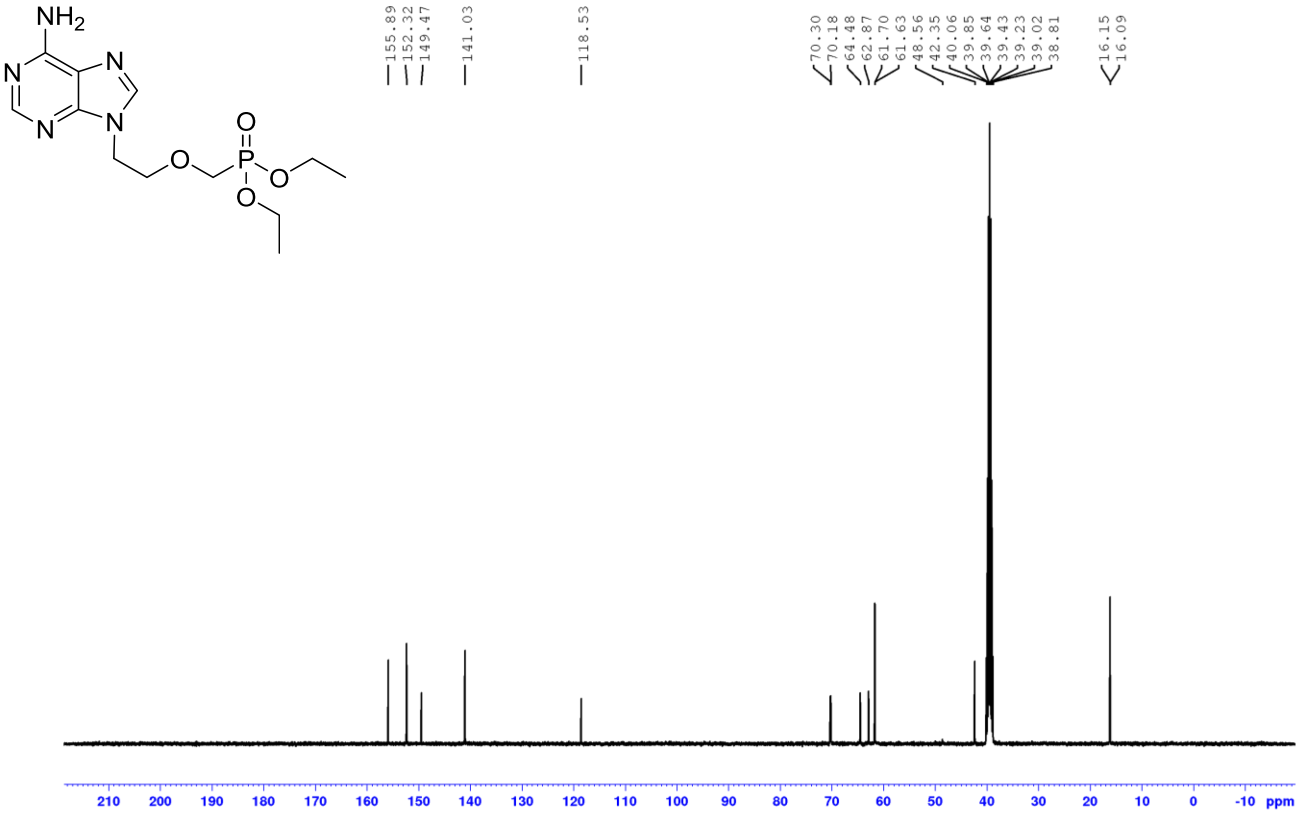

O,O-Diethyl N9-PMEA

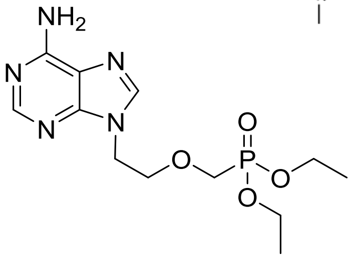

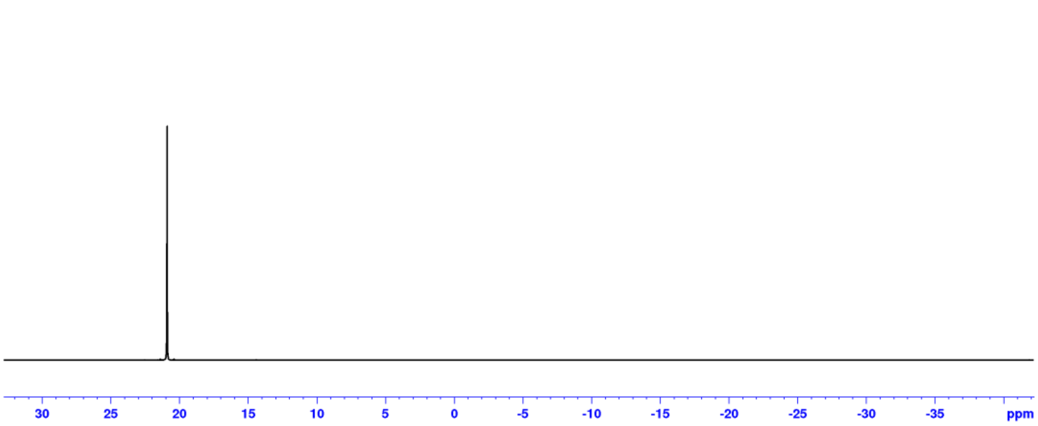

O,O-Diethyl N9-PMEA HMBC Spectrum (DEPT Q v 1H NMR)

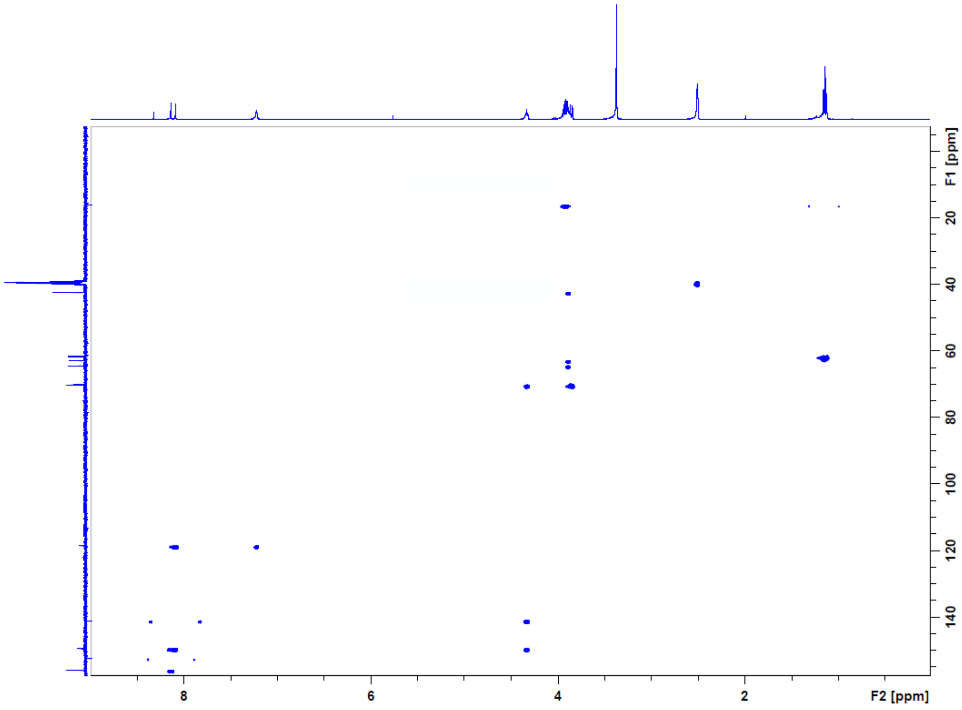

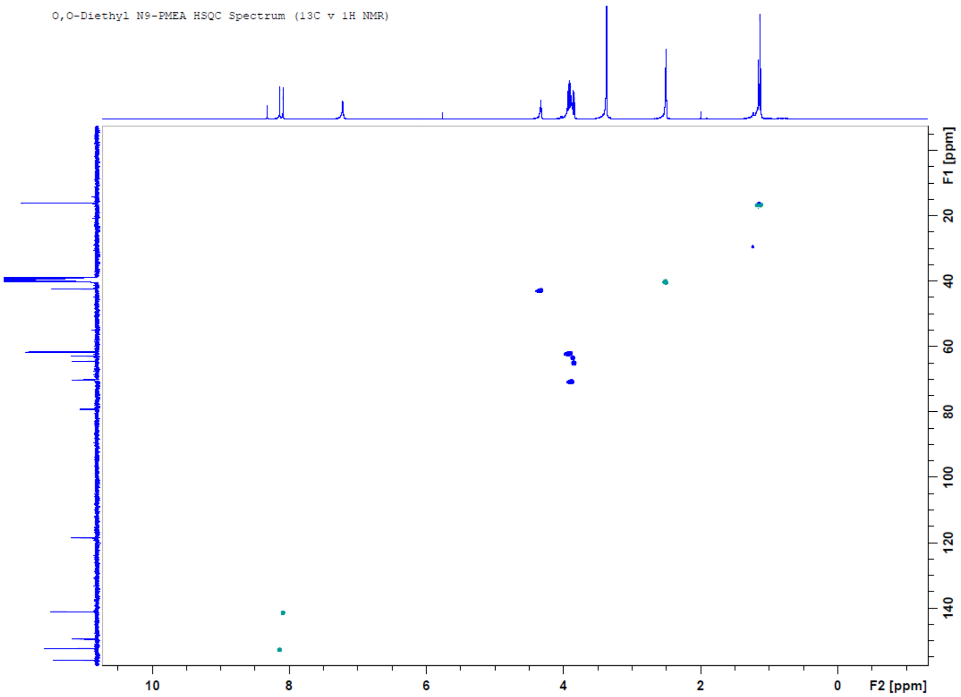

O,O-Diethyl N9-PMEA HSQC Spectrum (13C v 1H NMR)
F1 [ppm]
20
40
60
80
100
120
140
10
8
6
4
2
0
F2 [ppm]

O,O-Diethyl N7-PMEA
(minor regioisomer)

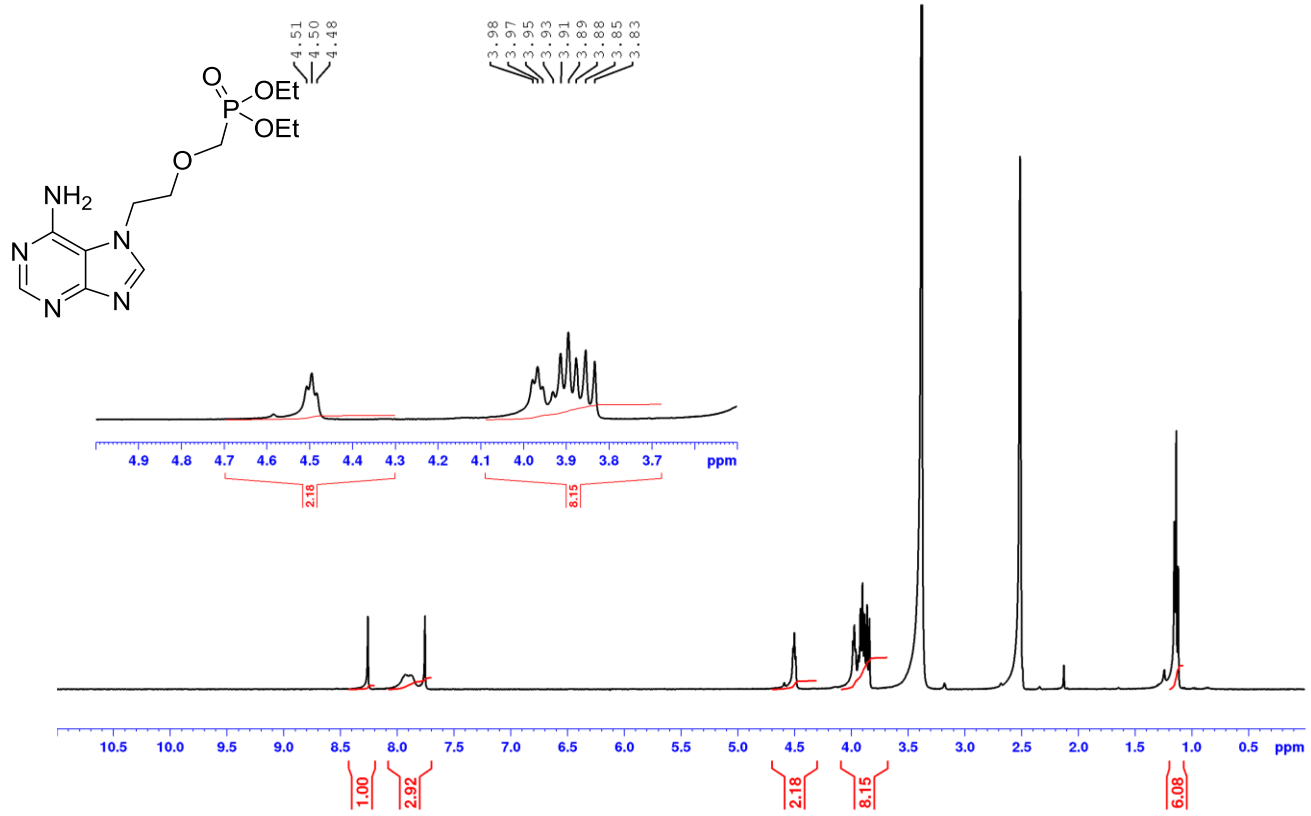

O,O-Diethyl N7-PMEA

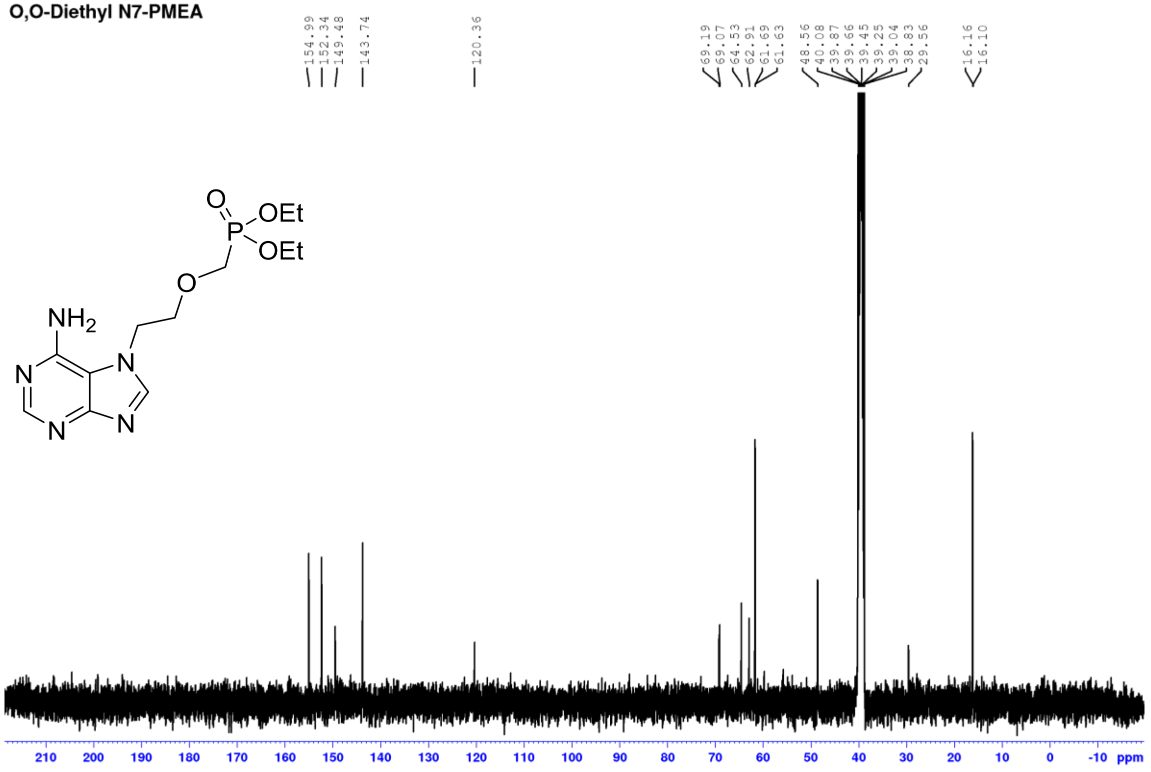

O,O-Diethyl N7-PMEA

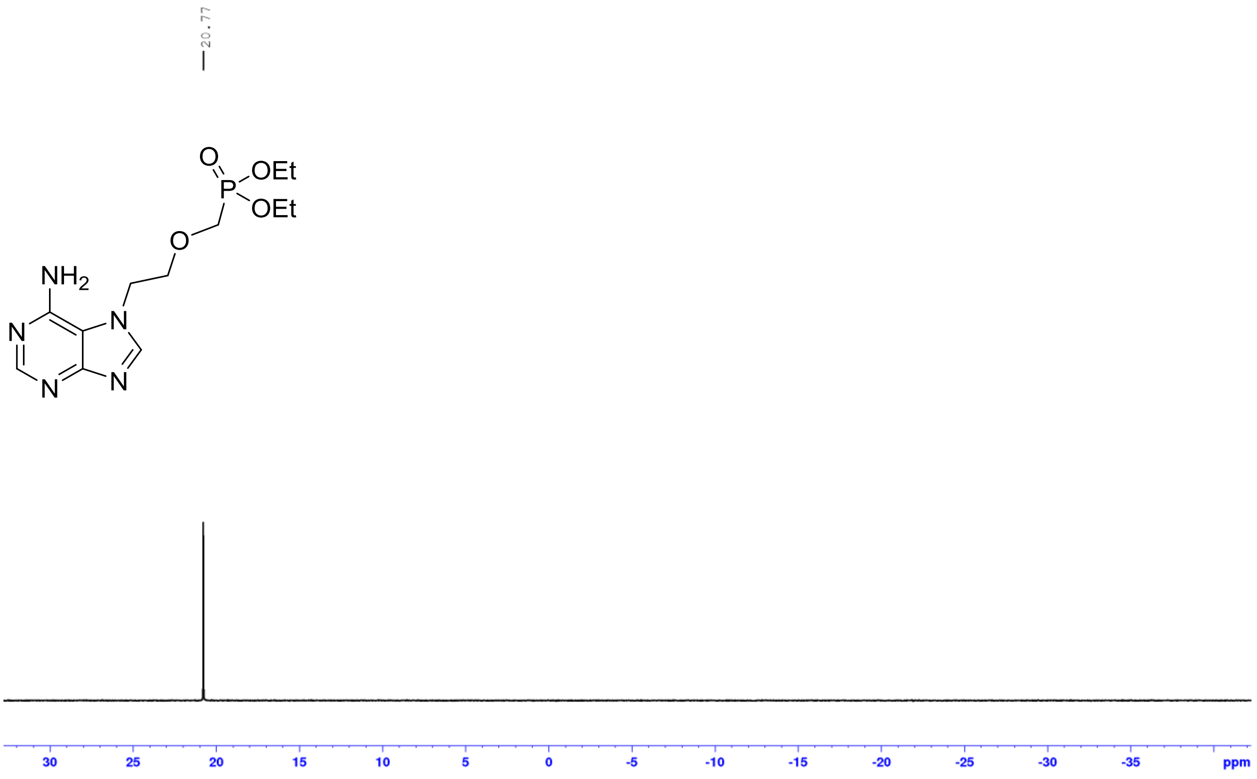

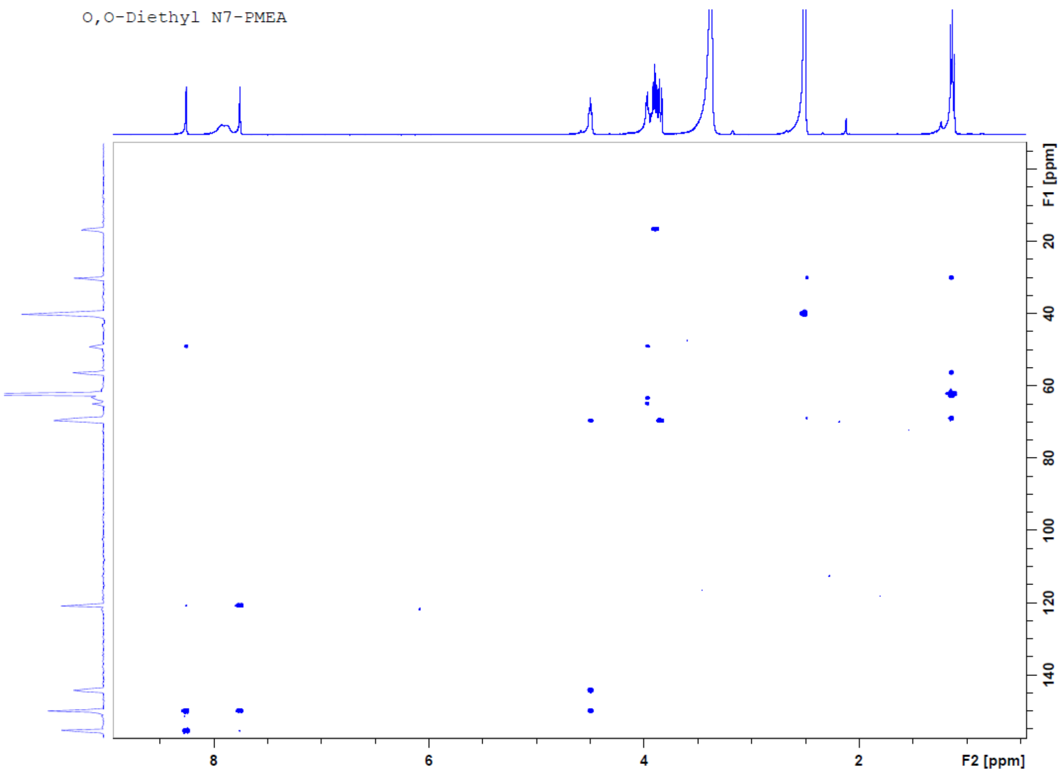

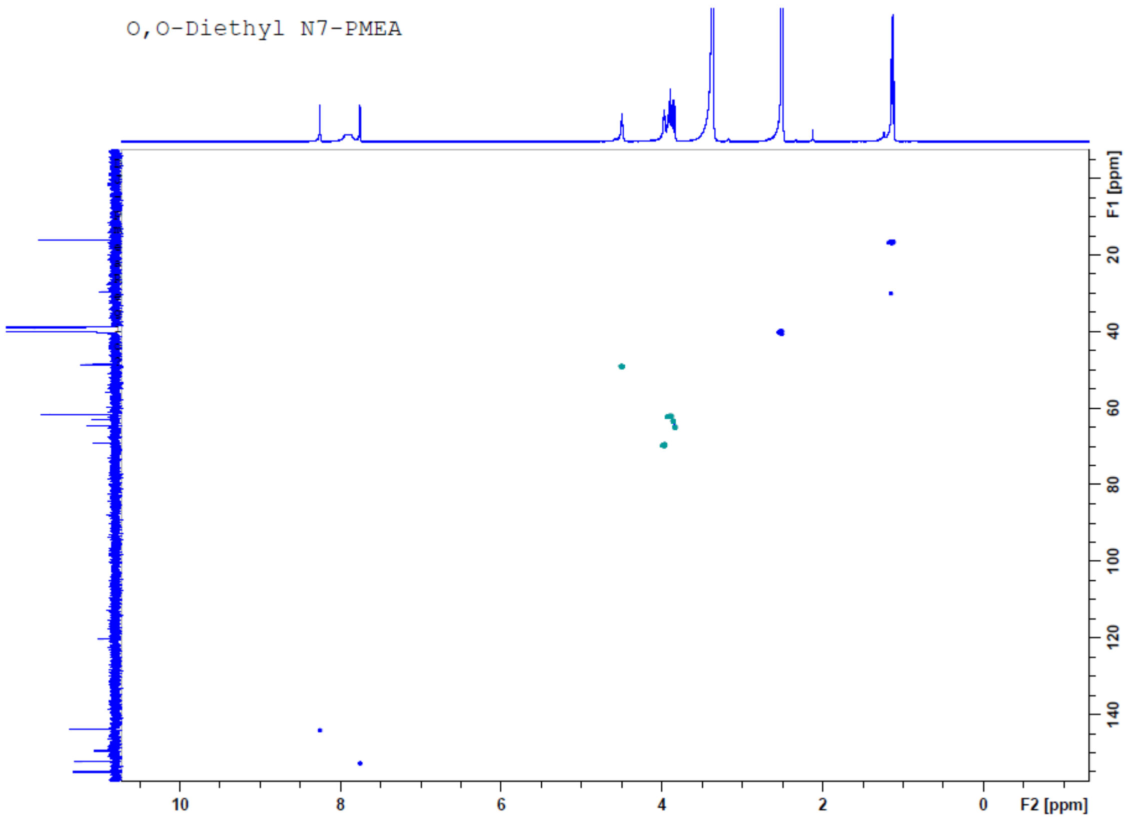
O,O-Diethyl N7-PMEA
F1 [ppm]
20
40
60
80
100
120
140
10
8
6
4
2
0
F2 [ppm]

O,O-diethyl ((2-(6-chloro-9H-purin-9-yl)ethoxy)methyl)phosphonate

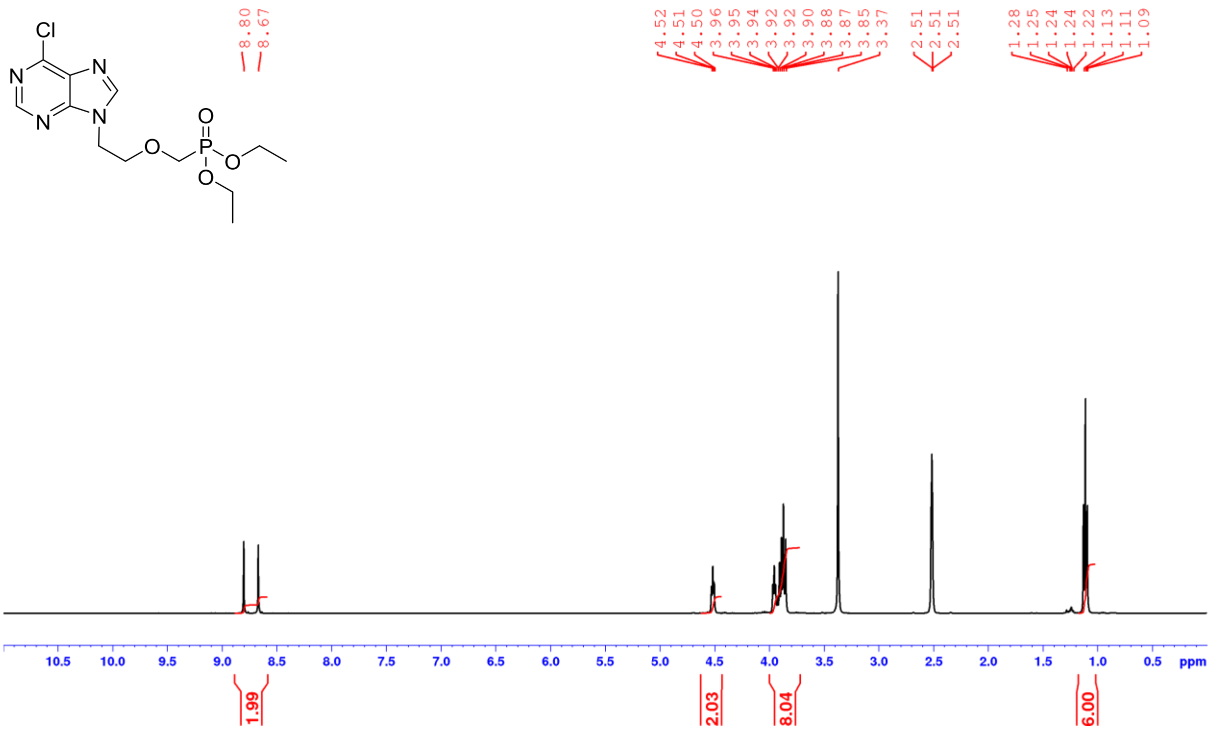

O,O-diethyl ((2-(6-chloro-9H-purin-9-yl)ethoxy)methyl)phosphonate

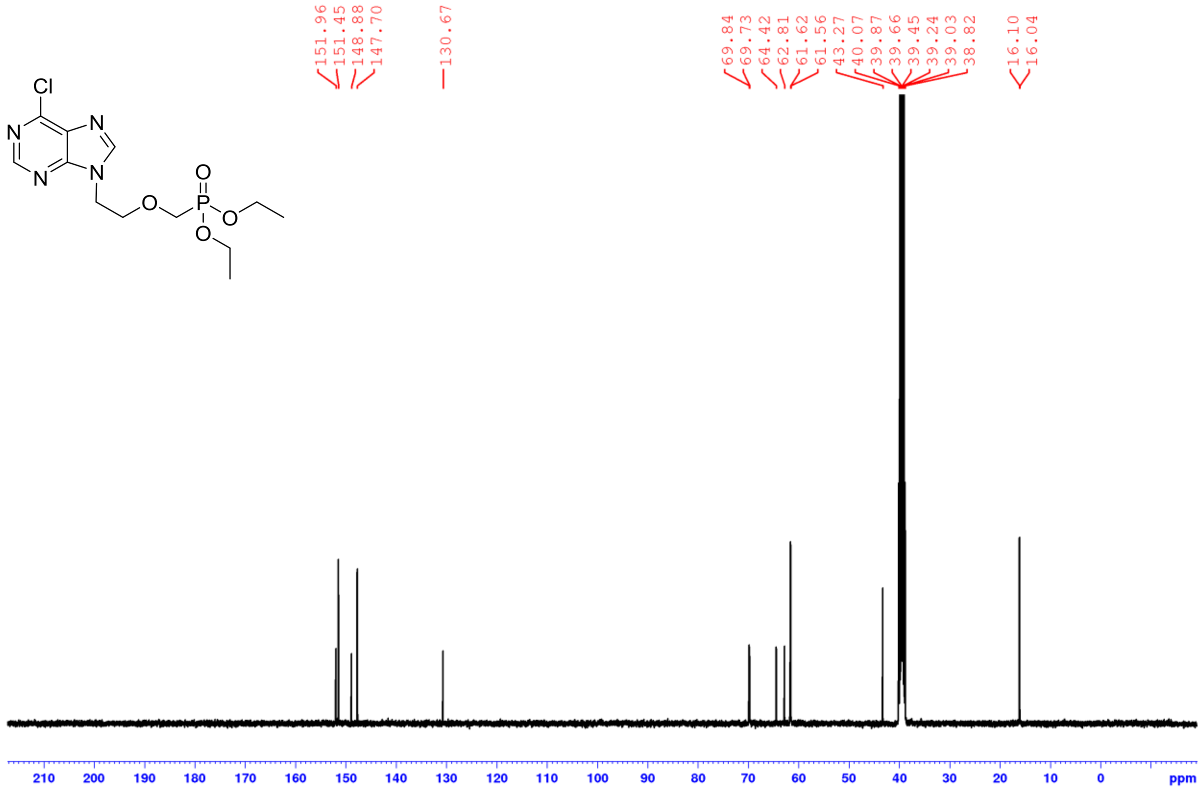

O,O-diethyl (2-(6-Chloro-9H-purin-9-yl)ethoxy)methyl)phosphonate

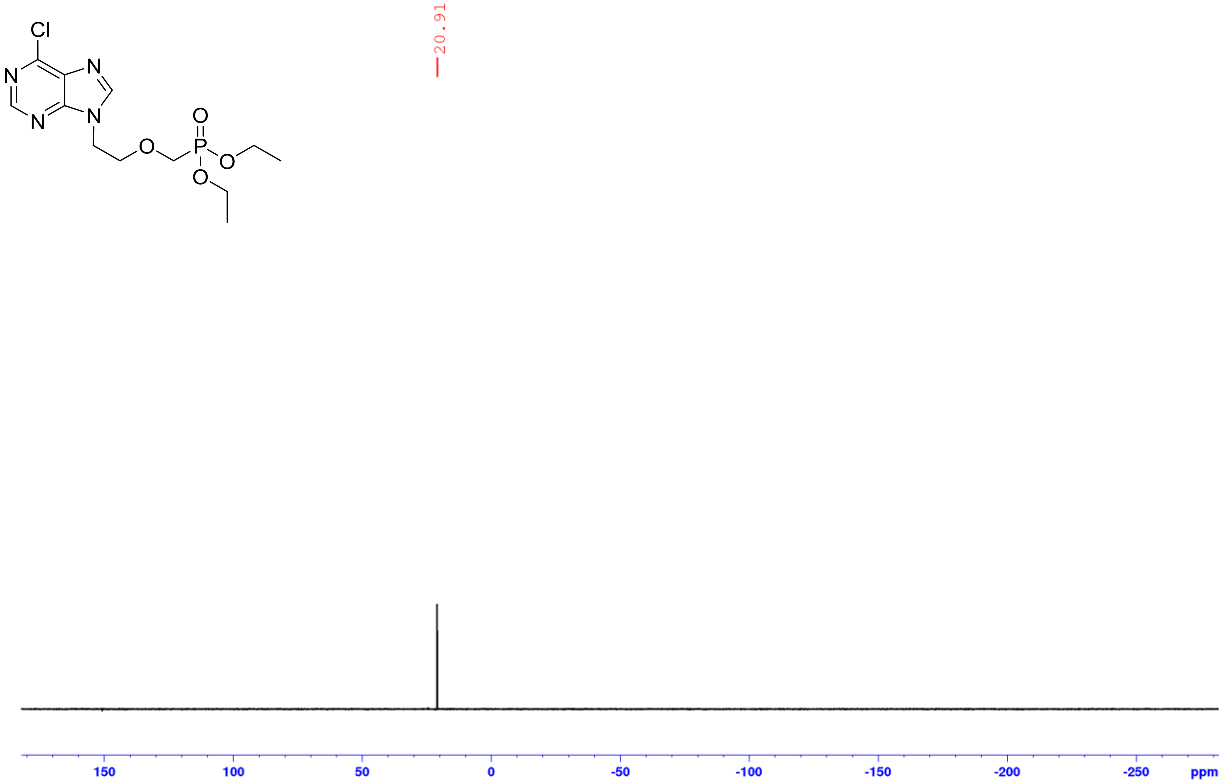

DJ - 6-chloropurine alkylation
Minor (N7) isomer

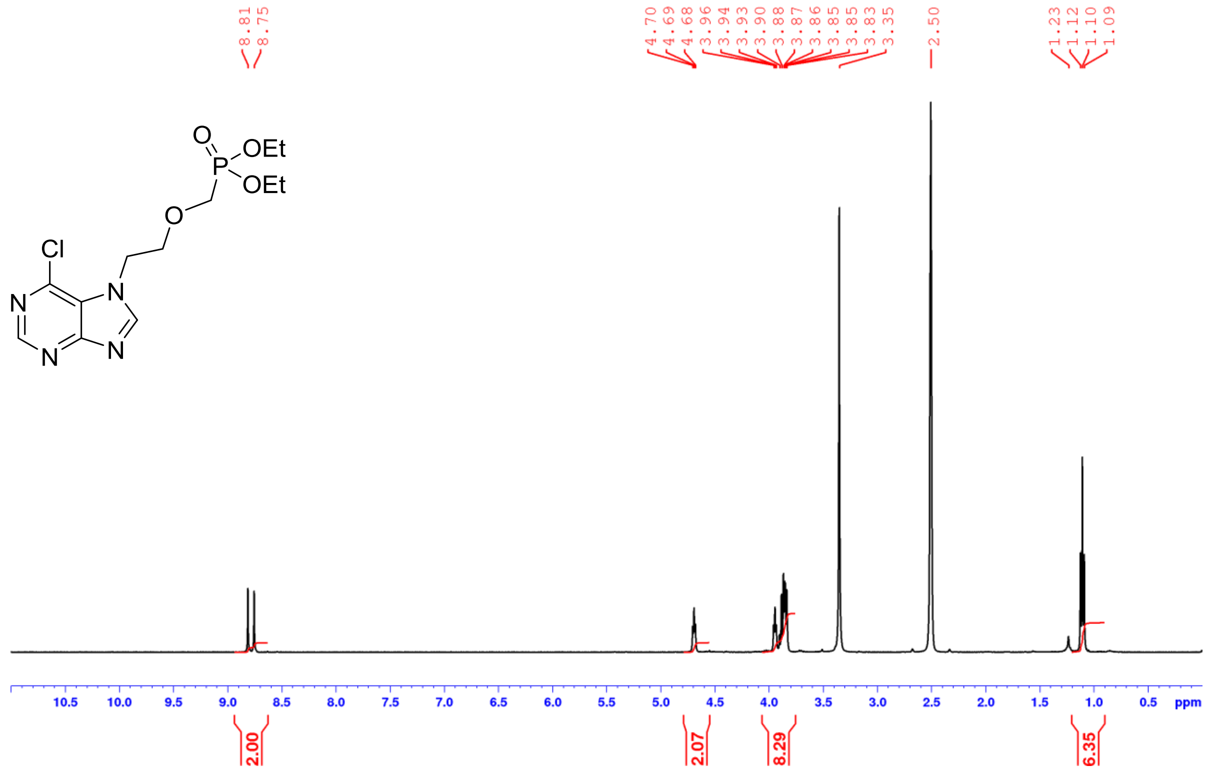

O,O-diethyl (2-(6-chloro-7H-purin-7-yl)ethoxy)methyl)phosphonate

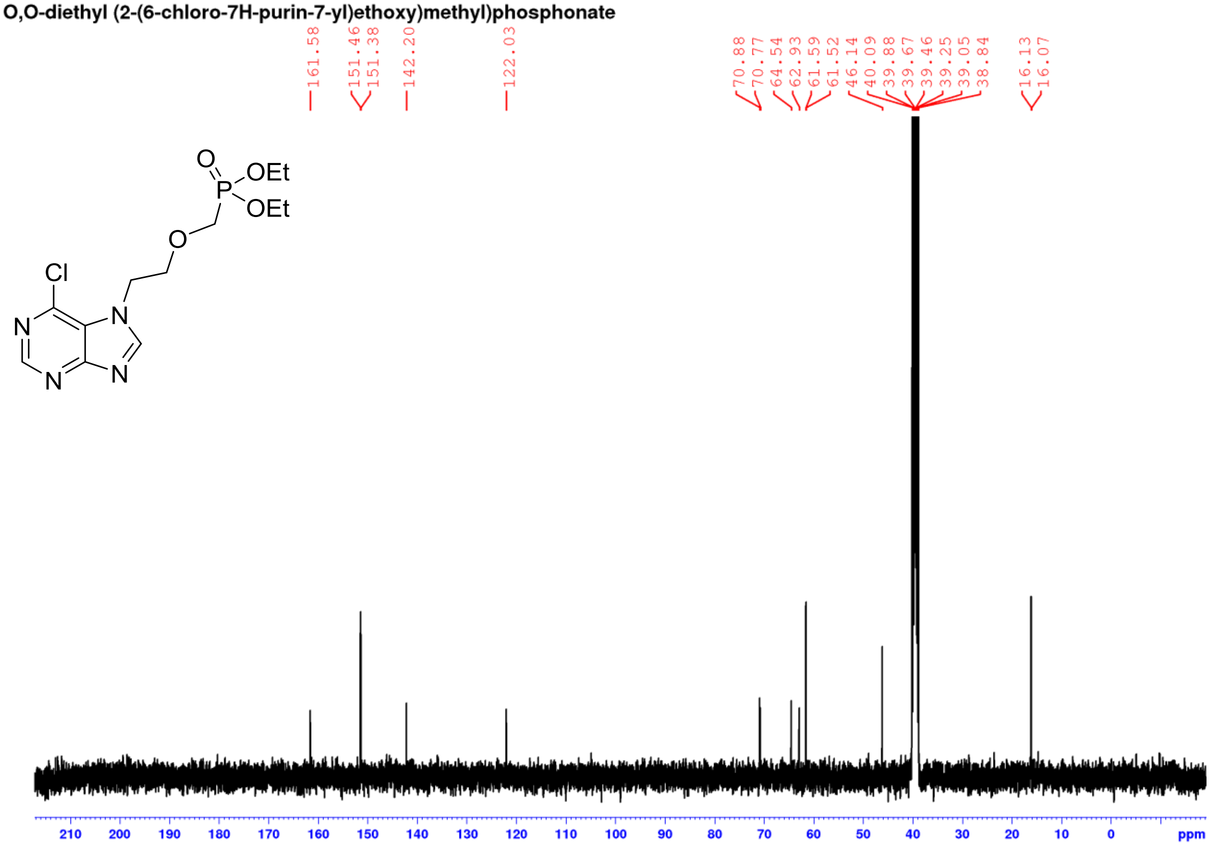

O,O-diethyl (2-((6-chloro-7H-purin-7-yl)ethoxy)methyl)phosphonate

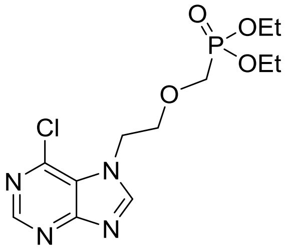

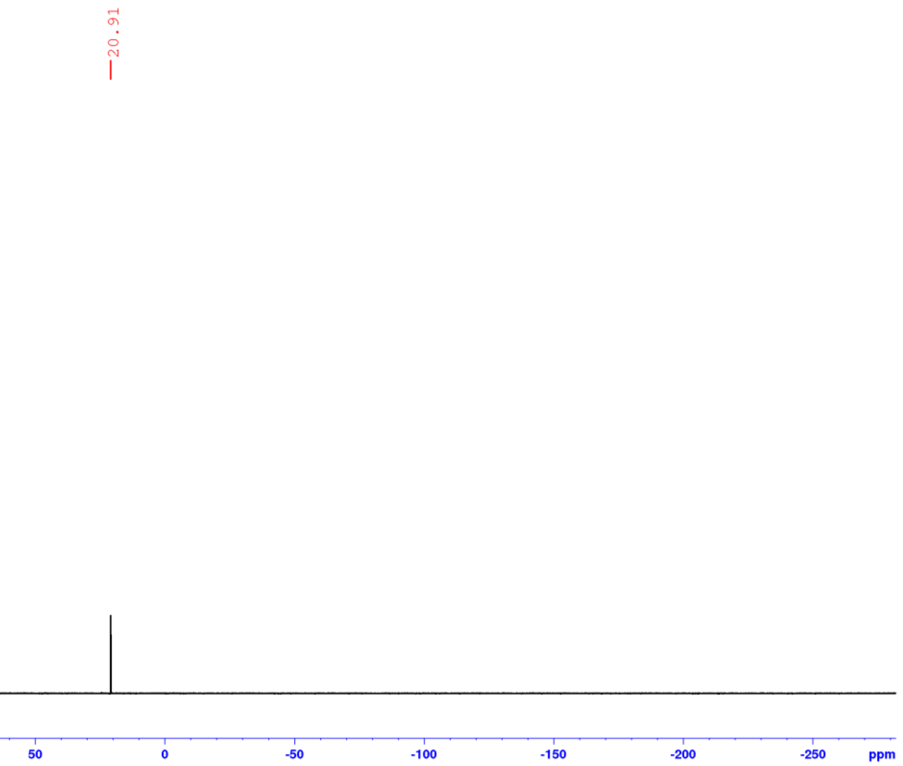

O,O-diethoxy ((2-(6-benzylamino)-9H-purin-9-yl)ethoxy)methyl)phosphonate

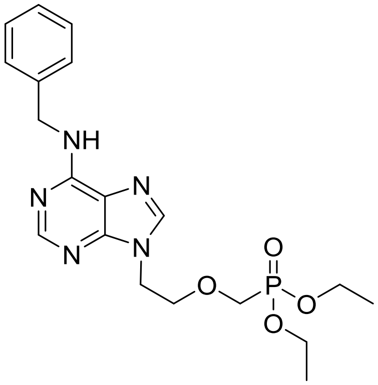

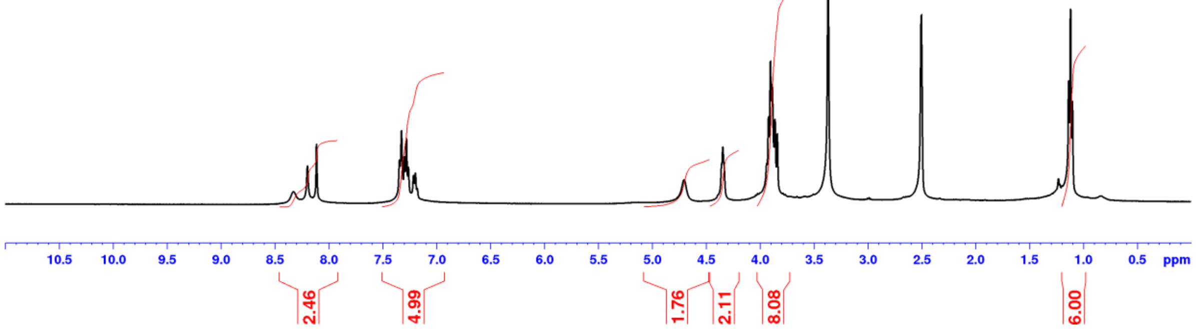

O,O-diethyl ((2-(6-benzylamino)-9H-purin-9-yl)ethoxy)methyl)phosphonate

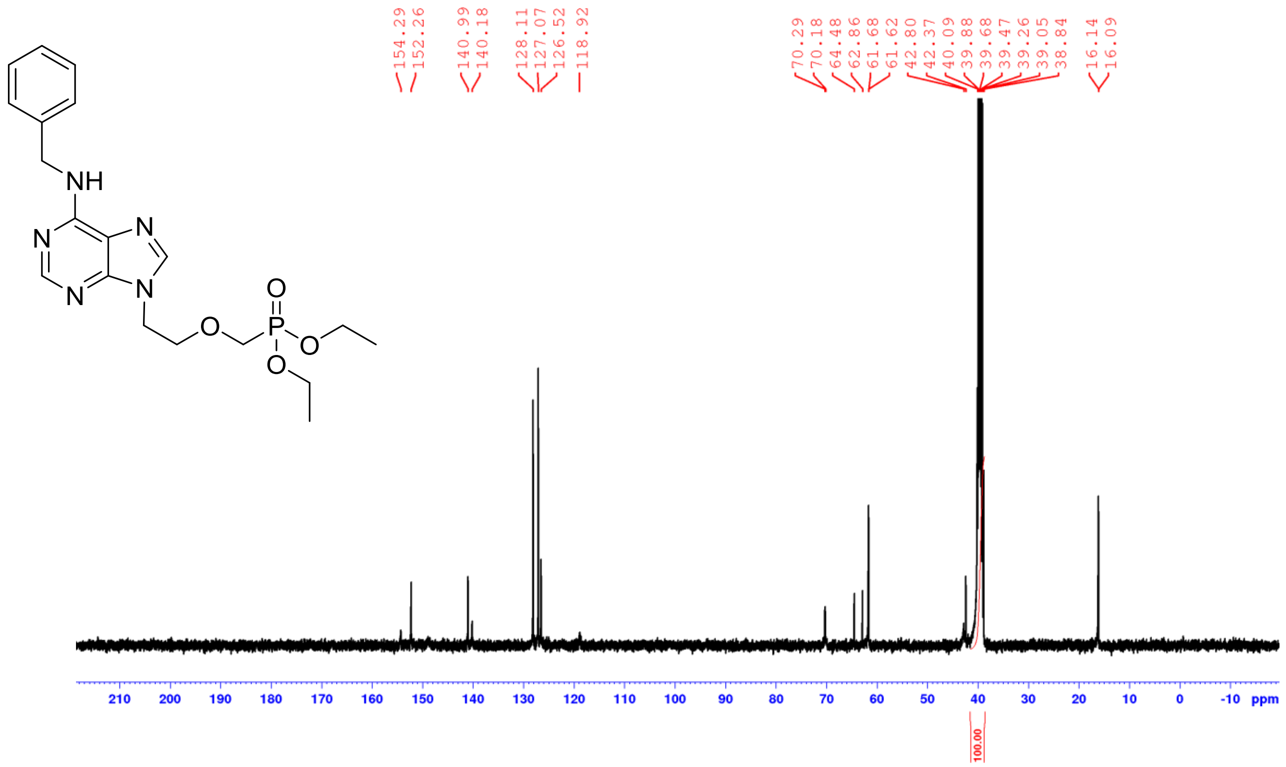

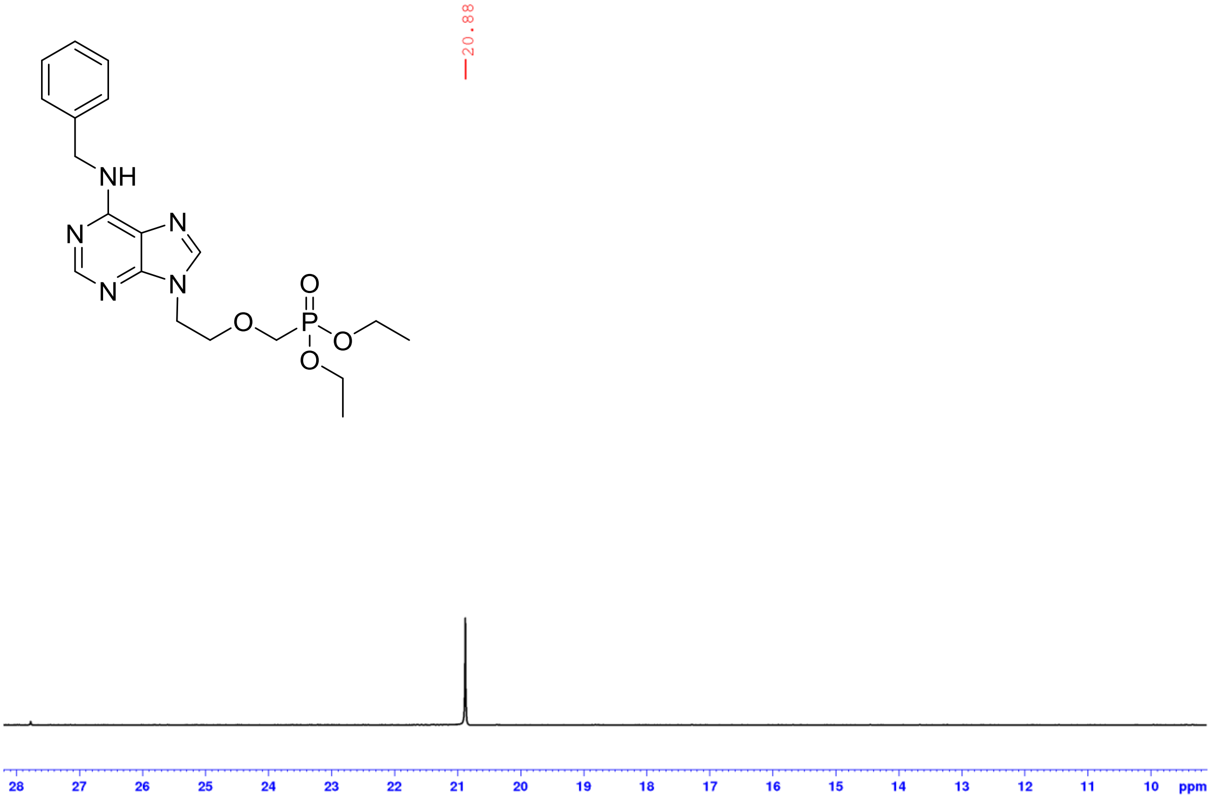

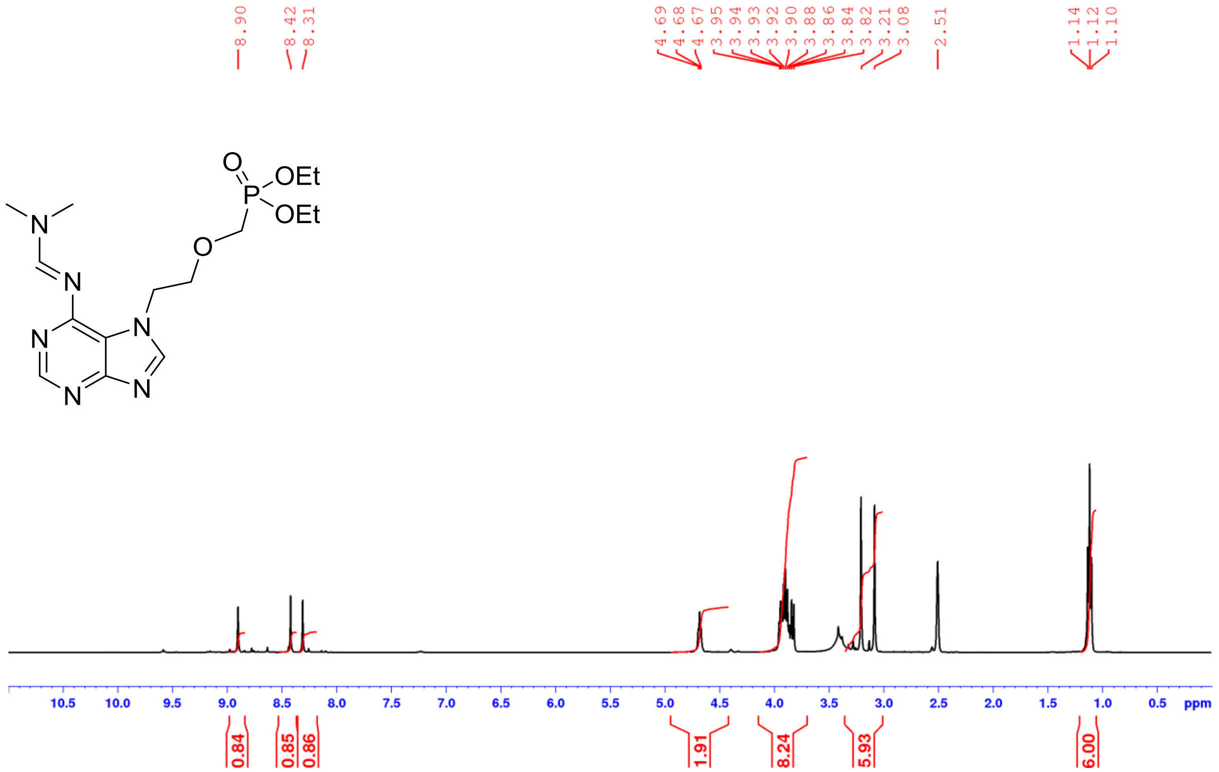
8.90
8.42
8.31
4.69
4.68
4.67
3.95
3.94
3.93
3.92
3.90
3.88
3.86
3.84
3.82
3.21
3.08
2.51
1.14
1.12
1.10
O
P
OEt
OEt
O
N
N
N
N
N
N
0.84
0.85
0.86
1.91
8.24
5.93
6.00
10.5
10.0
9.5
9.0
8.5
8.0
7.5
7.0
6.5
6.0
5.5
5.0
4.5
4.0
3.5
3.0
2.5
2.0
1.5
1.0
0.5
ppm

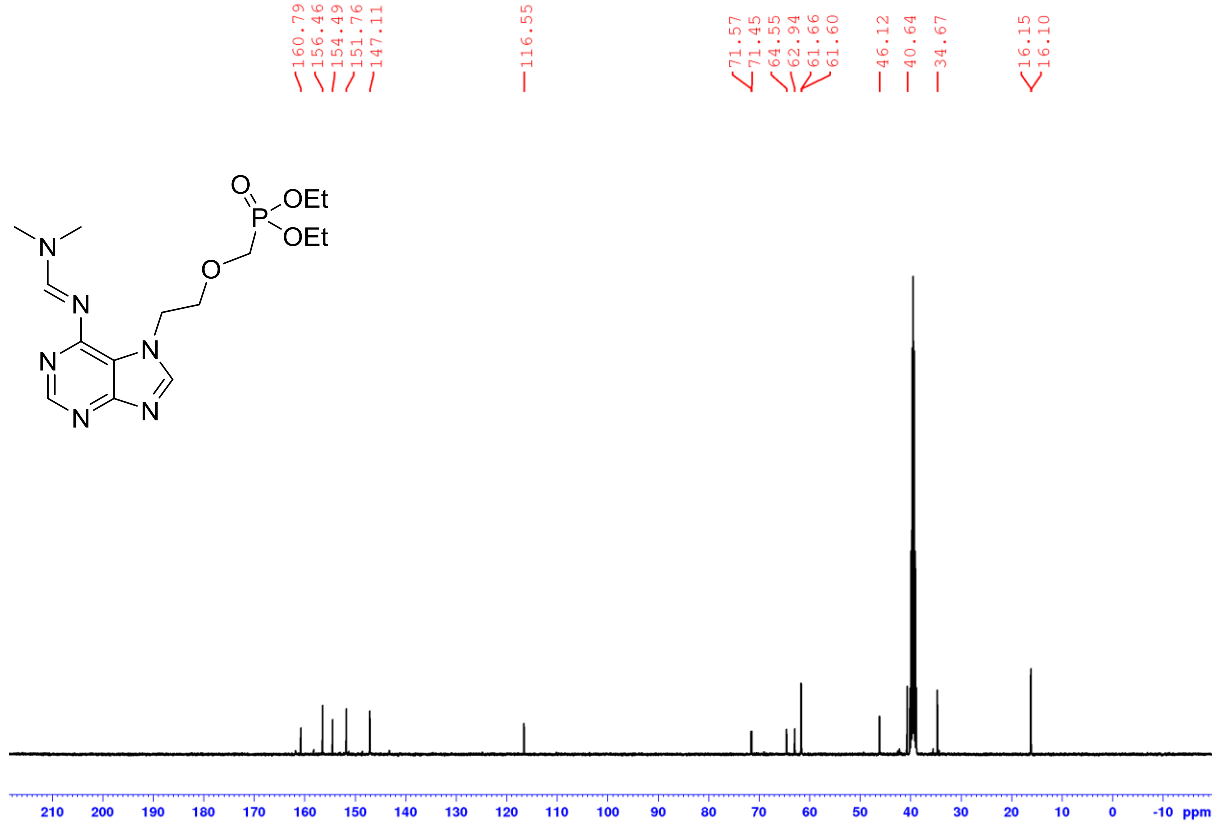
160.79
156.46
154.49
151.76
147.11
116.55
71.57
71.45
64.55
62.94
61.66
61.60
46.12
40.64
34.67
16.15
16.10
O
P
OEt
OEt
O
N
N
N
N
N
N
210
200
190
180
170
160
150
140
130
120
110
100
90
80
70
60
50
40
30
20
10
0
-10
ppm

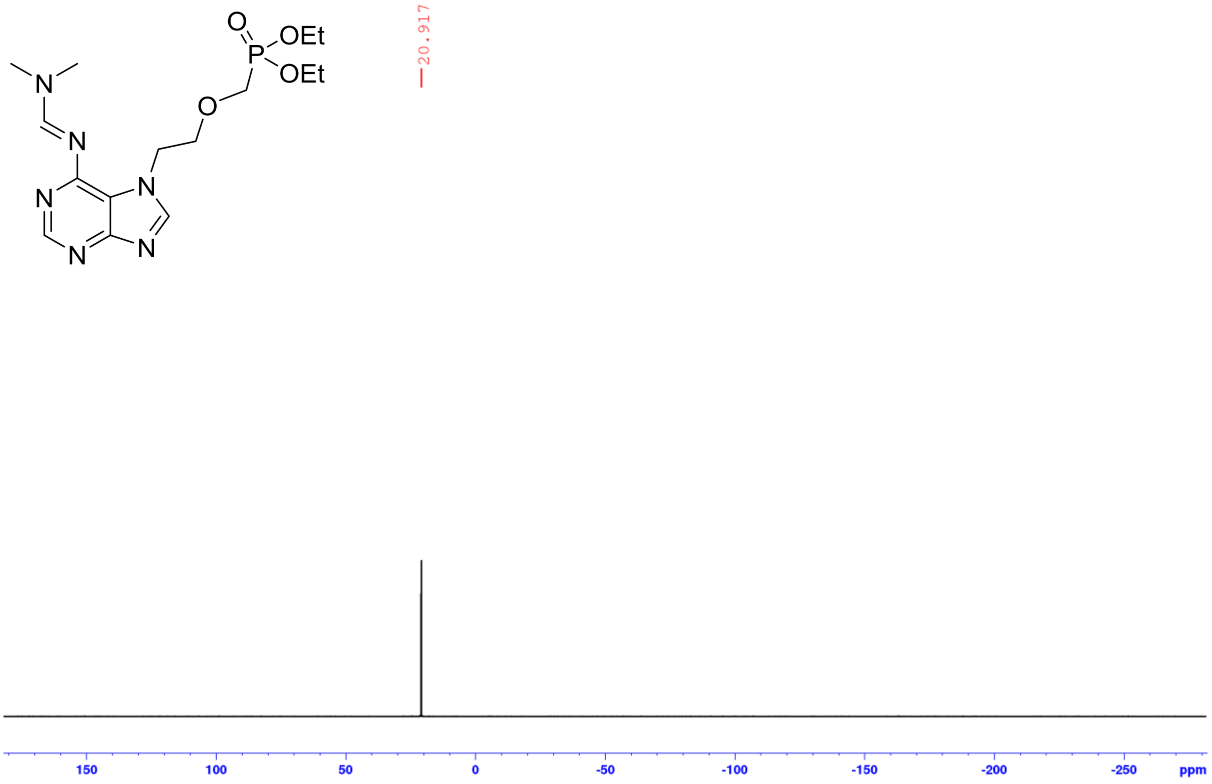

O
P
OEt
OEt
O
N
N
N
N
N
N
20.917
150
100
50
0
-50
-100
-150
-200
-250
ppm

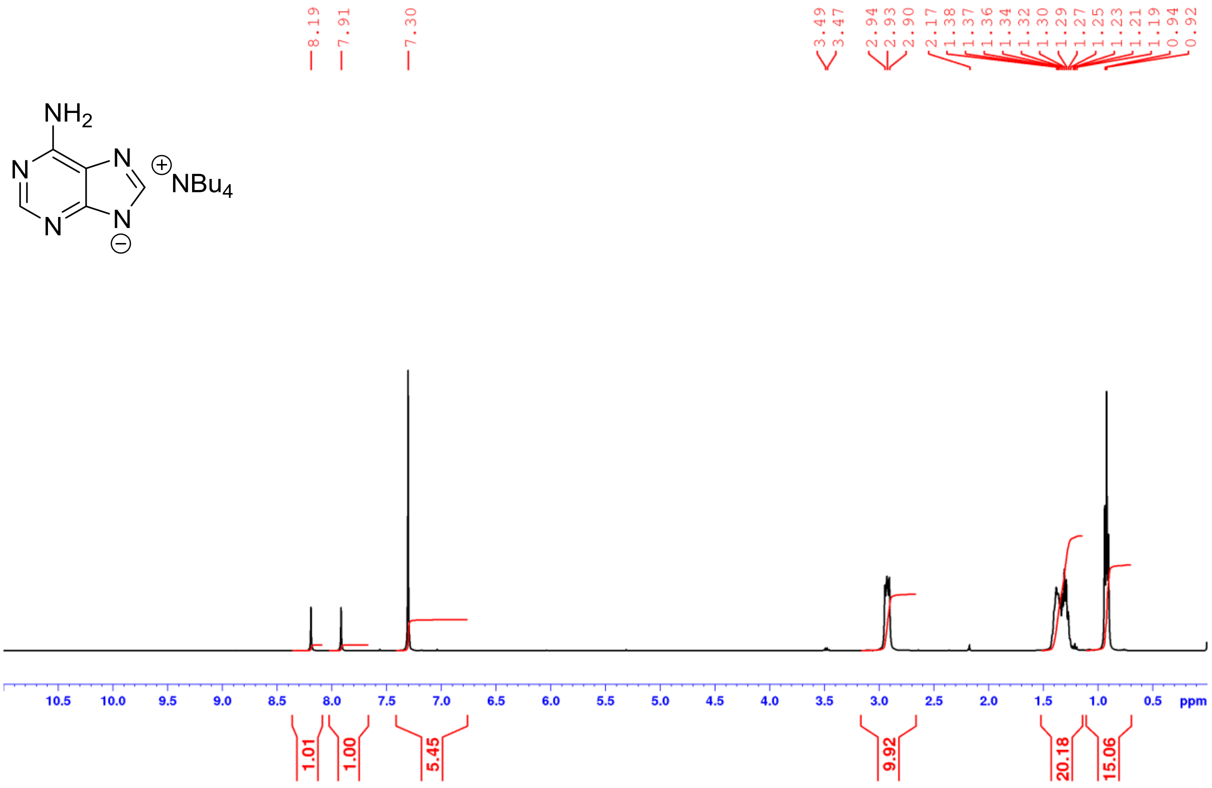

NH_2
N
N
N
N
NBu_4
8.19
7.91
7.30
3.49
3.47
2.94
2.93
2.90
2.17
1.38
1.37
1.36
1.34
1.32
1.30
1.29
1.27
1.25
1.23
1.21
1.19
0.94
0.92
10.5
10.0
9.5
9.0
8.5
8.0
7.5
7.0
6.5
6.0
5.5
5.0
4.5
4.0
3.5
3.0
2.5
2.0
1.5
1.0
0.5
ppm
1.01
1.00
5.45
9.92
20.18
15.06

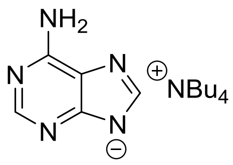

NH2
N
N
N
N
NBu4

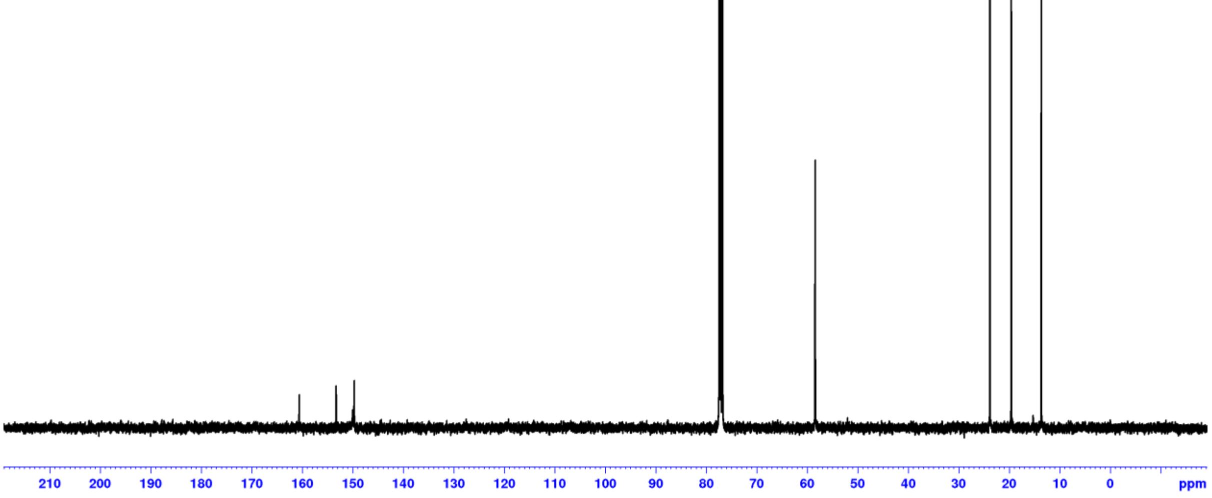

160.57
153.27
149.98
149.67
58.40
23.80
19.57
13.61
210
200
190
180
170
160
150
140
130
120
110
100
90
80
70
60
50
40
30
20
10
0
ppm

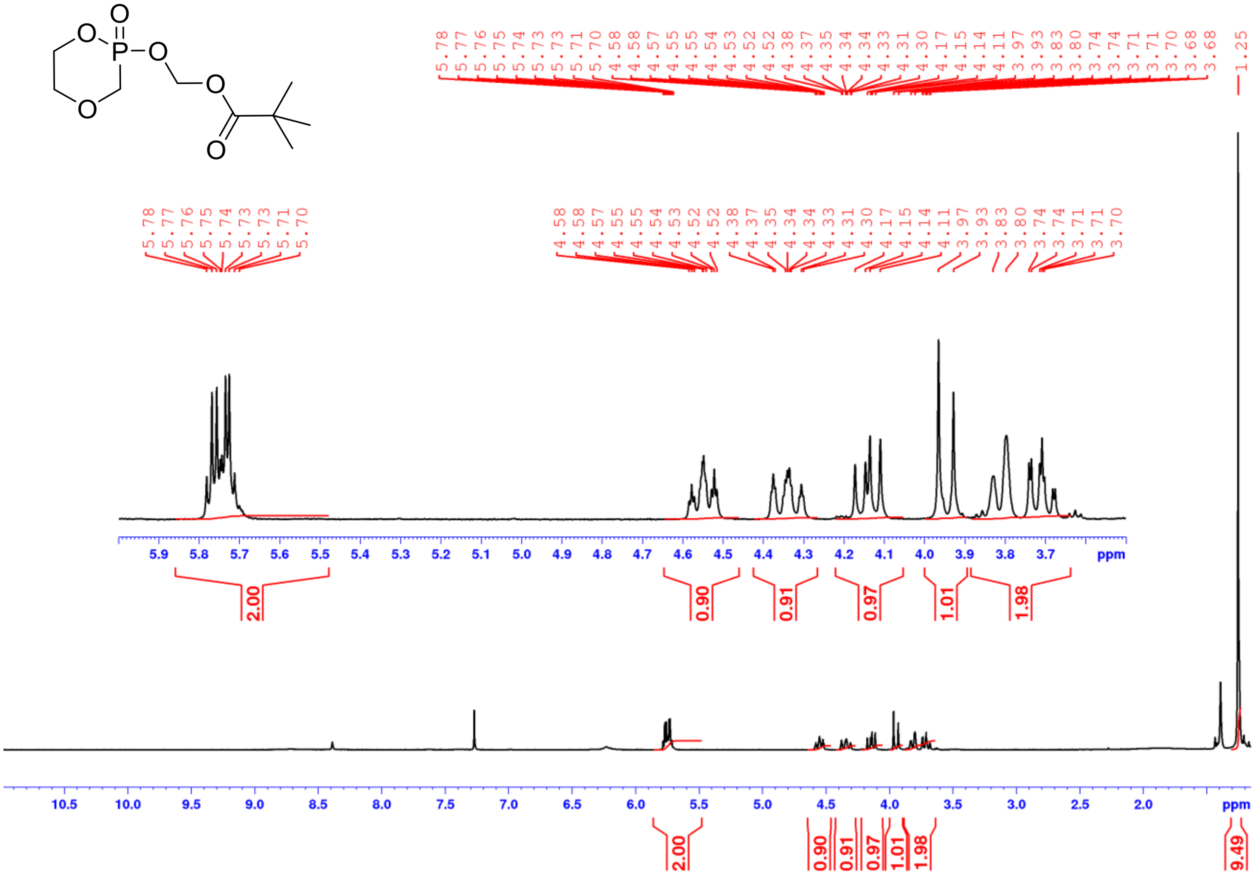

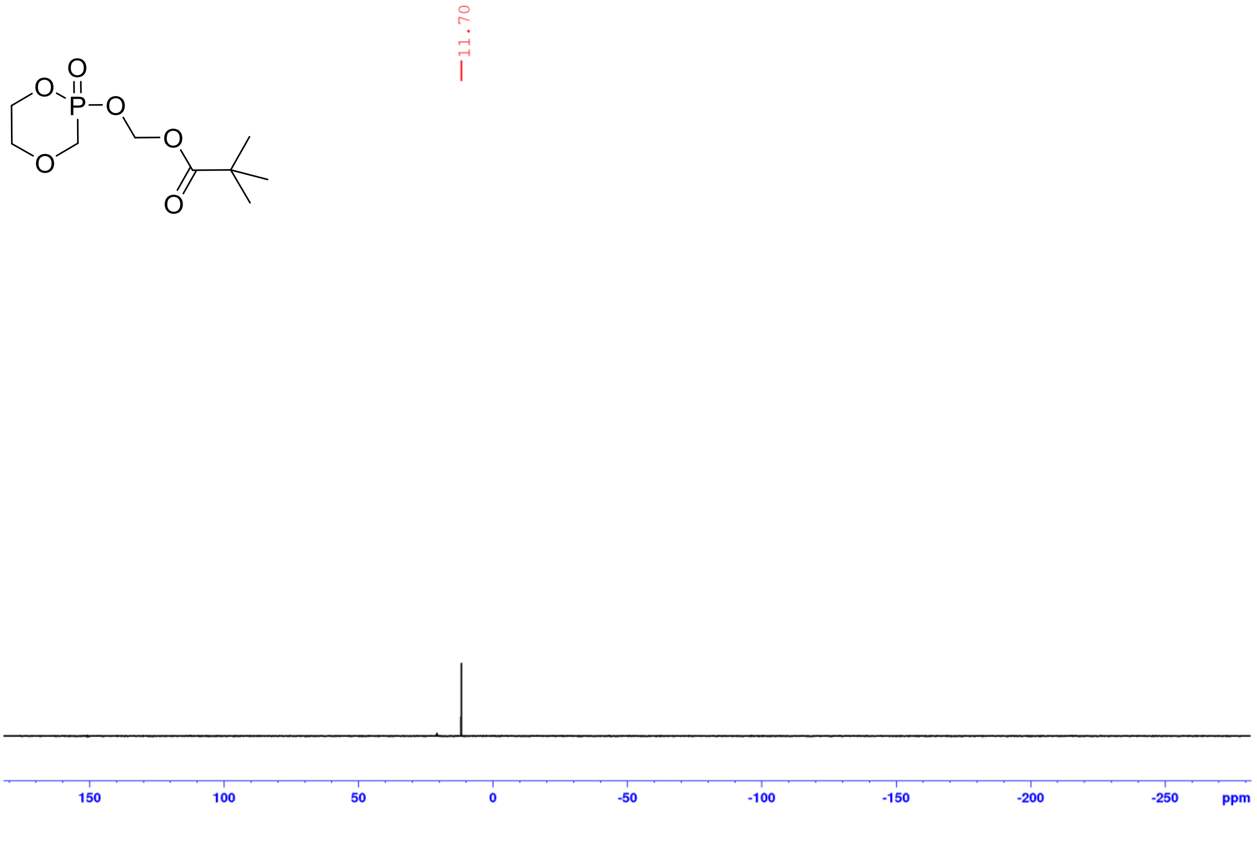

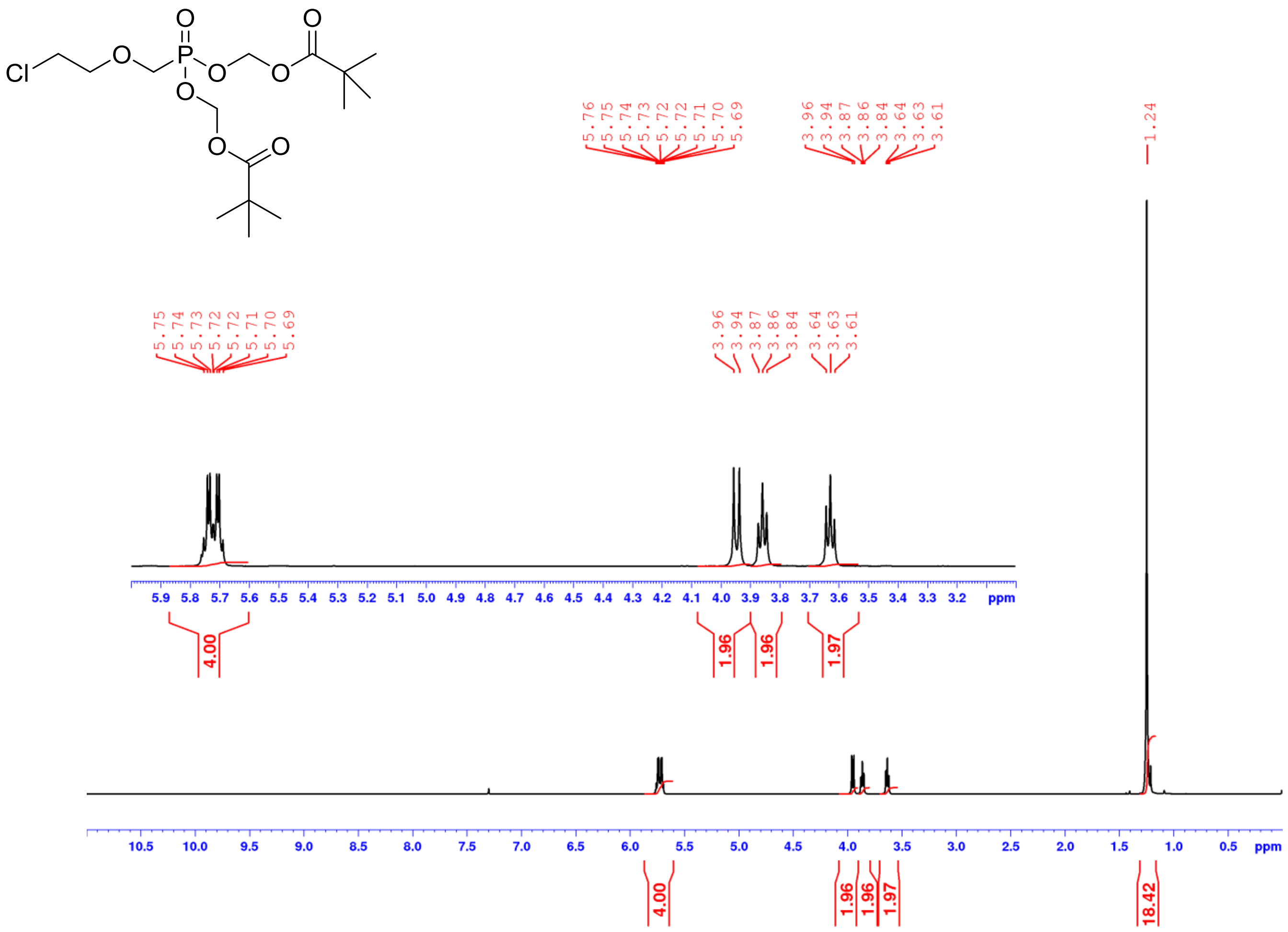

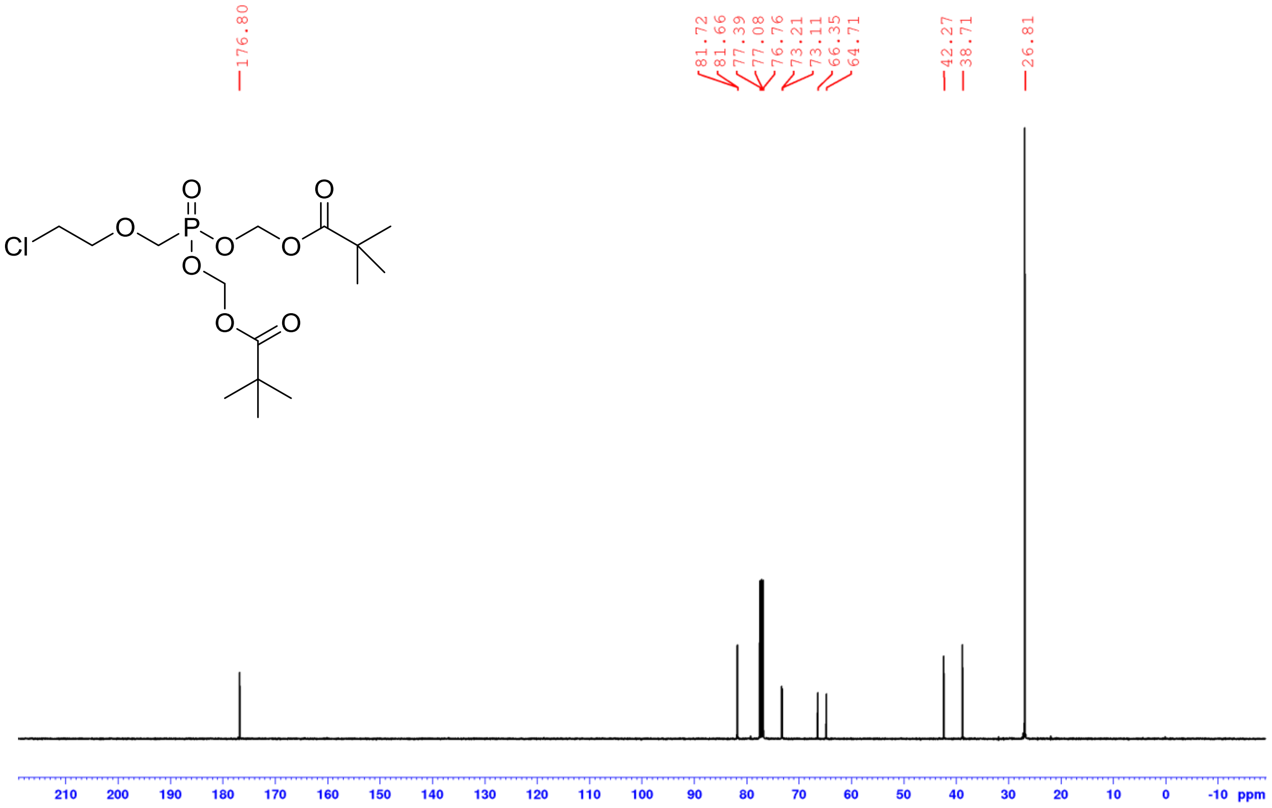

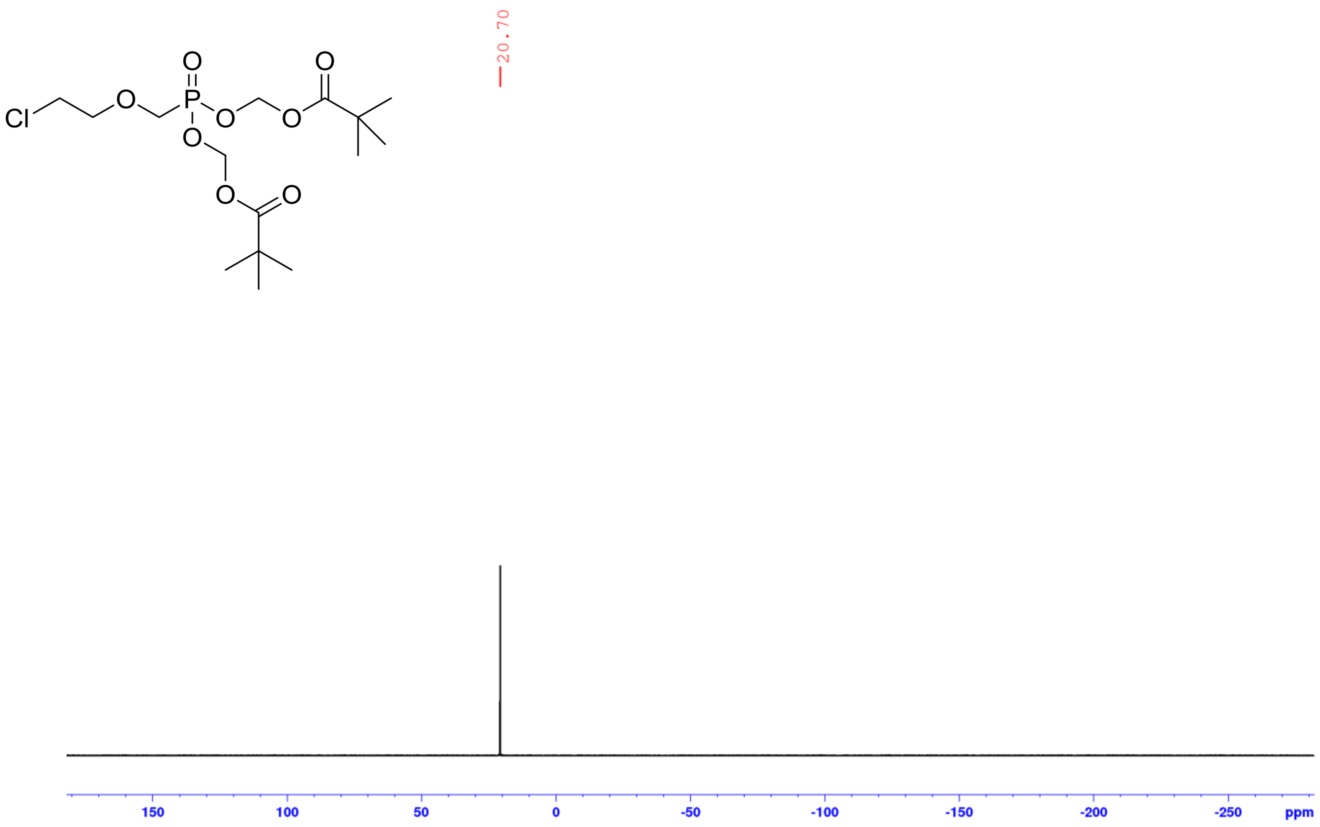

References

1. Bouhadir, K.; Abramian, L.; Ezzeddine, A.; Usher, K.; Vladimirov, N. *Molecules* **2012,** *17*, 13290.
2. Phillion, D. P.; Andrew, S. S. *Tetrahedron Lett.* **1986,** *27*, 1477-1480.
3. Parrish, J.; Tong, L.; Wang, M.; Chen, X.; Lansdon, E. B.; Cannizzaro, C.; Zheng, X.; Desai, M. C.; Xu, L. *Bioorg. Med. Chem. Lett.* **2013,** *23*, 1493-1497.
4. Roux, L.; Priet, S.; Payrot, N.; Weck, C.; Fournier, M.; Zoulim, F.; Balzarini, J.; Canard, B.; Alvarez, K. *Eur. J. Med. Chem.* **2013,** *63*, 869-881.
5. Fu, X.-Z.; Jiang, F.-J.; Ou, Y.; Fu, S.; Cha, Y.-F.; Zhang, S.; Liu, Z.-Y.; Zhou, W.; Wang, A.-M.; Wang, Y.-L. *Chin. Chem. Lett.* **2014,** *25*, 115-118.
6. Huang, L.-K.; Cherng, Y.-C.; Cheng, Y.-R.; Jang, J.-P.; Chao, Y.-L.; Cherng, Y.-J. *Tetrahedron* **2007,** *63*, 5323-5327.
7. Nocentini, A.; Bua, S.; Lomelino, C. L.; McKenna, R.; Menicatti, M.; Bartolucci, G.; Tenci, B.; Di Cesare Mannelli, L.; Ghelardini, C.; Gratteri, P.; Supuran, C. T. *ACS Med. Chem. Lett.* **2017,** *8*, 1314-1319.
8. Maiti, M.; Persoons, L.; Andrei, G.; Snoeck, R.; Balzarini, J.; Herdewijn, P. *ChemMedChem* **2013,** *8*, 985-993.
